# A phase III clinical trial of PXT3003 in Chinese patients with Charcot-Marie-Tooth disease type 1A

**DOI:** 10.1016/j.isci.2026.117284

**Published:** 2026-08-26

**Authors:** Xiaoxuan Liu, Wanqian Cao, Hongli Li, Chen Sun, Jingzheng Hao, Yuwei Da, Baoyu Yuan, Ning Wang, Shan Wu, Jun Liu, Zuneng Lu, Qi Fang, Zhi-Ying Wu, Liying Yuan, Jiaoting Jin, Haishan Jiang, Yaling Liu, Mingming Ma, Ling Wei, Jianglong Tu, Xiaoli Yao, Yuan Sun, Xuefan Yu, Yiming Liu, Di Zhong, Yun Xu, Yuming Xu, Runxiu Zhu, Dongsheng Fan, Ruxu Zhang

**Affiliations:** 1 Department of Neurology, Peking University Third Hospital, Beijing 100191, China; 2 Beijing Key Laboratory of Biomarker and Translational Research in Neurodegenerative Diseases, Beijing 100191, China; 3 Key Laboratory for Neuroscience, National Health Commission/Ministry of Education, Peking University, Beijing 100191, China; 4 Department of Neurology, The Third Xiangya Hospital of Central South University, Changsha 410013, China; 5 Center of Clinical Research, Genenet Pharmaceutical Co., Ltd., Tianjin, China; 6 Neurology Department, Xuanwu Hospital of Capital Medical University, Beijing, China; 7 Department of Neurology, Zhongda Hospital, Southeast University, Nanjing, China; 8 Department of Neurology, The First Affiliated Hospital of Fujian Medical University, Fuzhou, China; 9 Department of Neurology, Affiliated Hospital of Guizhou Medical University, Guiyang, China; 10 Department of Neurology, Ruijin Hospital, Shanghai Jiaotong University School of Medicine, Shanghai, China; 11 Department of Neurology, Renmin Hospital of Wuhan University, Wuhan, China; 12 Department of Neurology, The First Affiliated Hospital of Soochow University, Suzhou, China; 13 Department of Neurology, The Second Affiliated Hospital, Zhejiang University School of Medicine, Hangzhou, China; 14 Department of Neurology, The First Hospital of China Medical University, Shenyang, China; 15 Department of Neurology, The First Affiliated Hospital of Xi’an Jiaotong University, Xi’an, China; 16 Department of Neurology, Nanfang Hospital, Southern Medical University, Guangzhou, China; 17 Department of Neurology, The Second Hospital of Hebei Medical University, Shijiazhuang, China; 18 Department of Neurology, Henan Provincial People’s Hospital, Zhengzhou, China; 19 Department of Neurology, The First Affiliated Hospital of Anhui Medical University, Hefei, China; 20 Department of Neurology, The Second Affiliated Hospital of Nanchang University, Nanchang, China; 21 Department of Neurology, The First Affiliated Hospital of Sun Yat-sen University, Guangzhou, China; 22 Department of Neurology, Qilu Hospital, Cheeloo College of Medicine, Shandong University, Qingdao, China; 23 Department of Neurology, First Hospital of Jilin University, Changchun, China; 24 Department of Neurology, Qilu Hospital of Shandong University, Jinan, China; 25 Department of Neurology, The First Affiliated Hospital of Harbin Medical University, Harbin, China; 26 Department of Neurology, Nanjing Drum Tower Hospital, Nanjing, China; 27 Department of Neurology, The First Affiliated Hospital of Zhengzhou University, Zhengzhou, China; 28 Department of Neurology, Inner Mongolia People’s Hospital, Hohhot, China

**Keywords:** Charcot-Marie-Tooth disease type 1A, PXT3003, peripheral neuropathy, PMP22, phase 3 clinical trial, functional disability

## Abstract

Charcot-Marie-Tooth disease type 1A (CMT1A) is the most common inherited peripheral neuropathy and currently lacks disease-modifying therapy. PXT3003, a low-dose oral combination targeting PMP22 overexpression, has shown efficacy in two trials, while one recent confirmatory trial failed to meet its primary efficacy endpoints. In this trial, eligible participants aged 16 to 65 years with genetically confirmed mild-to-moderate CMT1A were randomly assigned to receive oral PXT3003 or placebo twice daily for 15 months. The primary endpoint was the change in the overall neuropathy limitations scale (ONLS) total score from baseline to month 15. At month 15, mean ONLS change from baseline was −0.268 (SD: 0.82) in the PXT3003 group versus 0.013 (SD: 0.65) in the placebo group, with a between-group difference of −0.249 (95% confidence interval [CI]: −0.467 to −0.030; *p* = 0.0257). Significant improvements were observed in the ONLS leg subscore and ankle-dorsiflexion strength. These findings support PXT3003 as a promising therapeutic option for alleviating limb symptoms in patients with CMT1A.

## Highlights

• PXT3003 addresses unmet medical needs as therapy for CMT1A, a rare hereditary neuropathy
• This trial shows robust ONLS gains and steady functional benefits across secondary endpoints
• PXT3003 shows favorable safety comparable to placebo with no treatment-related serious AEs

## Graphical abstract

**Figure:**
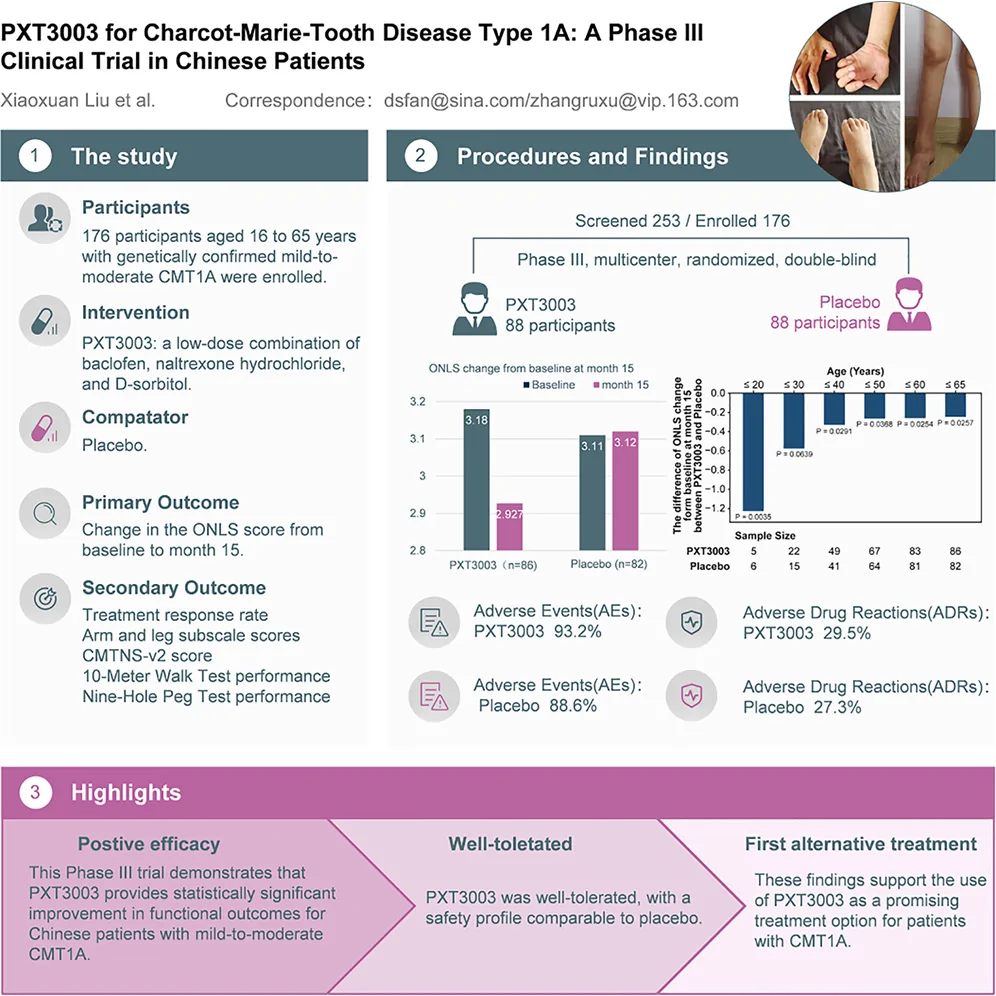

## Teaser

Health sciences; Internal medicine; Medical specialty; Medicine; Neurology

## Introduction

Charcot-Marie-Tooth (CMT) disease is the most common inherited disorder affecting the peripheral nervous system, with a prevalence of approximately 1 in 2,500 individuals.^1^ Its primary clinical manifestations include distal muscle weakness and atrophy; distal sensory loss; and, in some patients, limb deformity. CMT type 1A (CMT1A), the most prevalent subtype, accounts for 40%–50% of all CMT cases.^2^ It is caused by duplication of the 1.4-Mb region on chromosome 17p11.2, leading to overexpression of the *PMP22* gene. Patients with CMT1A typically have the classic CMT phenotype, with symptoms often developing in the first two decades of life.^3^ Currently, there is no curative treatment for CMT1A, and management is primarily focused on supportive therapies, such as rehabilitation and orthopedic interventions.^4^

PXT3003, a low-dose combination of baclofen, naltrexone hydrochloride, and d-sorbitol, was formulated using network pharmacology to regulate PMP22 expression via the screening of marketed drugs. The agents bind to GABA, opioid, and muscarinic acetylcholine receptors, respectively, working synergistically to decrease intracellular cyclic AMP (cAMP) levels, which in turn downregulate *PMP22* gene expression in Schwann cells and neurons. Preclinical studies have demonstrated that PXT3003 improves nerve conduction velocity and ameliorates motor activity in transgenic CMT1A rat models with three extra copies of the PMP22 gene.^5^^,^^6^^,^^7^

Three clinical trials have been completed to assess PXT3003 in patients with CMT1A. A 1-year phase II French clinical trial (NCT01401257) and a phase III multicenter study (NCT02579759) with an extended observation period of 15 months showed that a higher PXT3003 dose (containing baclofen 12 mg, naltrexone hydrochloride 1.4 mg, and d-sorbitol 420 mg/day) benefited patients with mild-to-moderate CMT1A.^8^^,^^9^ However, a recently completed confirmatory phase III clinical trial (NCT04762758) failed to meet its primary efficacy endpoints.

PXT3003 was generally well tolerated, with a favorable safety profile. Nevertheless, a notable rate of participant dropout attributed to drug crystallization (NCT02579759) warrants further investigation.

## Results

**Figure 1:**
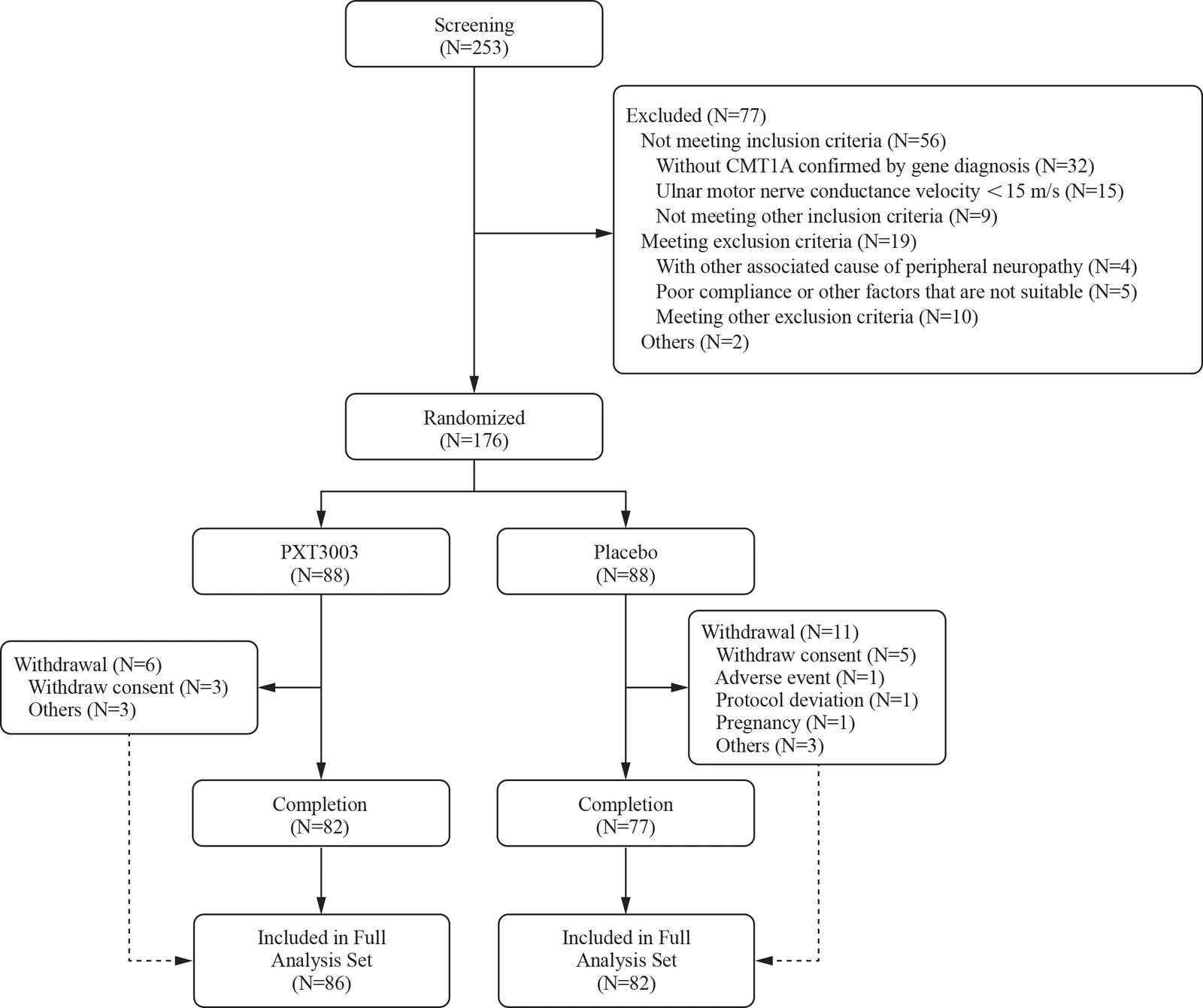
Randomization and treatment of patients

From September 26, 2021, to March 18, 2024, a total of 253 potential participants were screened, of whom 176 met the eligibility criteria and were randomized in a 1:1 ratio to receive either PXT3003 or a placebo. All 176 participants subsequently received their assigned treatment. The study completion rate was high, with 159 participants (90.3%) completing the study and only 17 (9.7%) withdrawing prematurely. The screening process is summarized in Figure 1. The primary analysis included 86 participants in the PXT3003 group and 82 in the placebo group. Baseline demographic and clinical characteristics were well-balanced between the two groups (Table 1). Interim analysis results indicated that the *p* value for the primary outcome was greater than 0.003. Consequently, per the prespecified protocol, the sample size remained unchanged given a conditional power (CP) of ≥80%.

**Table 1:** Demographic and clinical characteristics at baseline

	Placebo group (*N* = 82)	PXT3003 group (*N* = 86)
Age (years)	40.6 (11.41)	39.5 (12.13)

**Sex (*n*, %)**		

Male	46 (56.1%)	50 (58.1%)
Female	36 (43.9%)	36 (41.9%)
Height (cm)	165.53 (7.33)	166.85 (9.26)
Weight (kg)	64.34 (11.79)	65.08 (13.71)
BMI (kg/m^2^)	23.41 (3.58)	23.21 (3.55)
ONLS score	3.11 (0.93)	3.19 (0.89)
ONLS srm sub-score	1.27 (0.77)	1.33 (0.76)
ONLS leg sub-score	1.84 (0.46)	1.86 (0.41)
CMTNS-v2 total score	12.9 (3.12)	13.0 (2.89)
10MWT (s)	8.52 (1. 81)	8.33 (2.66)
9HPT (s)	27.45 (8. 58)	26.10 (6.50)
Dorsiflexion strength (N)	16.23 (15. 77)	15.32 (17.26)
Hand grip strength (kg)	22.88 (9. 77)	22.96 (9.74)

**Electrophysiological Parameters**		

Ulnar CMAP (mV)	3.14 (2.15)	3.42 (2.75)
Radial SNAP (μV)	0.67 (1.70)	0.80 (2.93)
Ulnar MCV (m/s)	20.08 (4.39)	20.44 (5.34)
Radial DML (ms)	7.64 (2.61)	7.80 (3.01)

**Quality-of-life scores**		

EQ-5D total score	7.8 (2.52)	7.6 (2.·21)
EQ-VAS	78.9 (17.20)	80.1 (15.48)

The number of precipitants in the PXT3003 group for height, weight, and BMI statistics is 85. CMTNS-V2, Charcot-Marie-Tooth neuropathy score, second version; MCV, motor conduction velocity; 10MWT, time 10m walk test; 9HPT, nine-hole peg test; N, Newtons; CMAP, compound motor action potential.

### Efficacy

**Figure 2:**
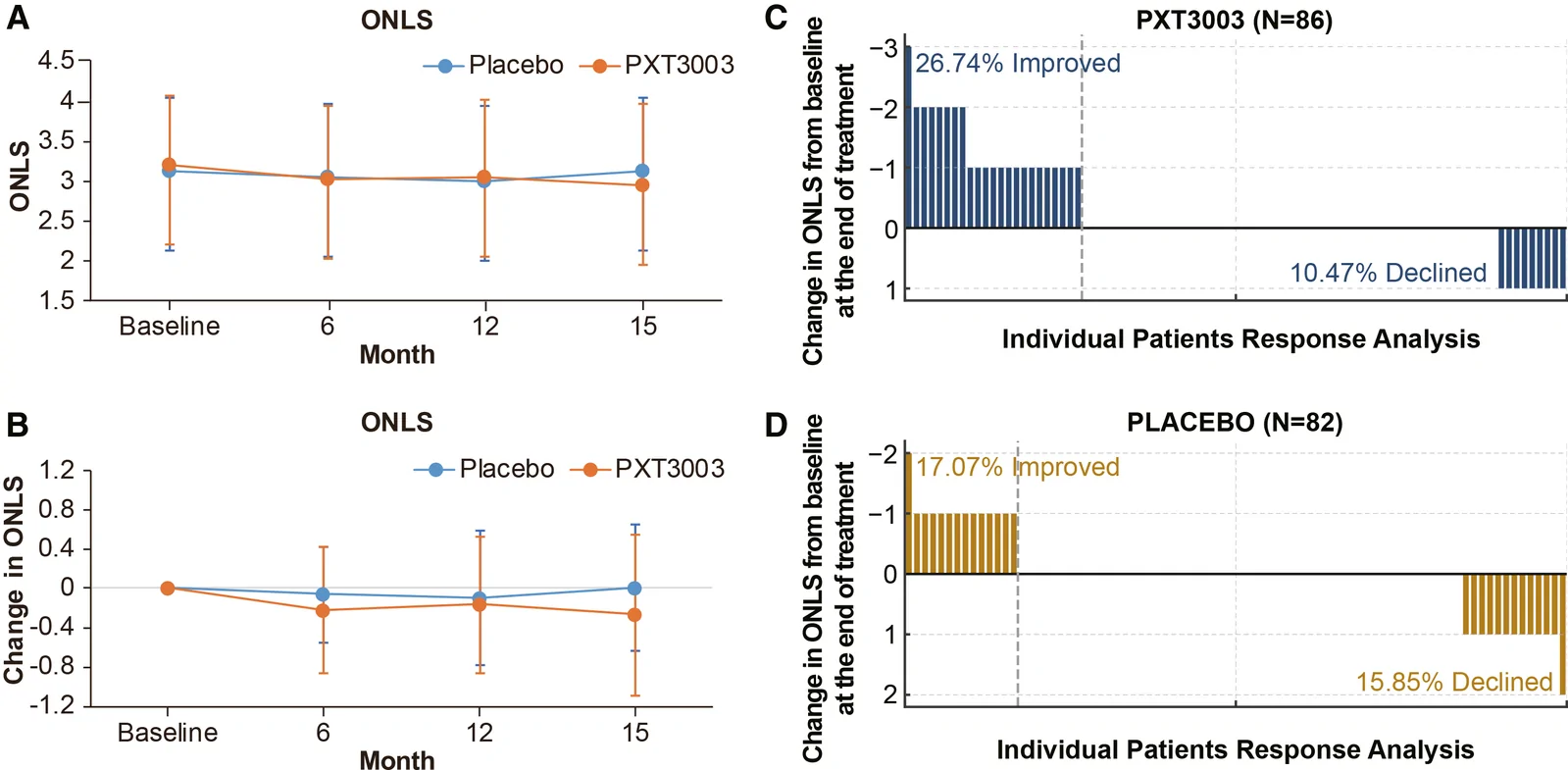
Efficacy assessment based on ONLS over 15 months (A) Trend of ONLS score changes. (B) Trend of ONLS score changes relative to baseline. Data in (A) and (B) are presented as mean ± SD, with error bars representing the standard deviation from the mean. (C and D) Distribution of changes in ONLS scores from baseline among patients.

For the primary endpoint, the mean overall neuropathy limitations scale (ONLS) score in the PXT3003 group decreased from a baseline of 3.186 (SD: 0.89) to 2.927 (SD: 1.04) at month 15. Conversely, the score in the placebo group remained stable, increasing slightly from 3.110 (SD: 0.93) to 3.120 (SD: 0.93). The changes from baseline were −0.268 for the PXT3003 group and 0.013 for the placebo group. A notable divergence between the two groups was observed between months 12 and 15, with the PXT3003 group showing a more pronounced improvement (Figure 2). The primary analysis revealed a between-group difference of −0.249 (95% CI: −0.467 to −0.030, *p* = 0.0257) in the change from baseline at month 15. Both prespecified and post hoc sensitivity analyses supported the robustness of the primary analysis (Table S1). We also compared the responsive rate and found that a higher proportion of patients in the PXT3003 group achieved improvement (defined as a ≥1-point decrease in ONLS) compared with the placebo group (26.74% vs. 17.07%, Figure 2).

At month 15, the PXT3003 group showed a statistically significant improvement in the ONLS leg sub-score compared with the placebo group (intergroup difference: −0.142, 95% CI: −0.248 to −0.037, *p* = 0.0083). Similarly, the PXT3003 group exhibited a significantly greater improvement in bilateral average dorsiflexion strength by standard dynamometer (N) (intergroup difference: 4.426, 95% CI: 0.051 to 8.800, *p* = 0.0474) (Table 2).

**Table 2:** Patient outcomes (fall analysis set)

	Placebo (*N* = 82)	PXT3003 (*N* = 86)	Groups difference	*p* value
**Primary efficacy outcome**				

**ONLS**	0.013 (0.65)	−0.268 (0.82)	−0.249 (−0.467, −0.030)	00257

**Secondary efficacy endpoints**				

ONLS arm subscore	0.000 (0.64)	−0.122 (0.64)	−0.091 (−0.269, 0.087)	0.3153
ONLS leg subscore	0.013 (0.20)	−0.146 (0.45)	−0.142 (−0.248, −0.037)	0.0083∗
Response rate 1^a^ (n, %)	14 (17.1%)	23 (26.7%)	9.7% (−2.9%, 21.8%)	0.1410
Response rate 2^b^ (n, %)	69 (84.1%)	77 (89.5%)	5.4% (−5.0%, 16.0%)	0.3632
**CMTNS-v2 total score**	−0.6 (2.82)	−1.0 (2.78)	−0.307 (−1.159, 0.545)	0.4804
**10MWT** (**S**)	0.047 (3.6125)	−0.139 (2.1050)	−0.257 (−1.153, 0.639)	0.5739
**9HPT** (**S**)	−0.346 (4.0059)	−0.015 (4.4379)	0.125 (−1.136, 1.387)	0.8459
**Dorsiflexion strength** (**N**)	1.062 (11.8778)	5.697 (16.4263)	4.426 (0.051, 8.800)	0.0474
**Grip strength (kg)**	0.0258 (8.9938)	1.9110 (9.2511)	1.758 (−0.945, 4.461)	0.2024

**Electrophysiological parameters**				

Ulnar CMAP (mV)	0.3216 (2.2491)	0.2909 (1.6711)	0.032 (−0.435, 0.499)	0.8927
Radial SNAP (μV)	0.3019 (1.9306)	−0.2084 (3. 2094)	−0.346 (−0.879, 0.188)	0.2043
Ulnar DML (ms)	−0.402 (1.7214)	0.056 (2.0762)	0.201 (−0.297, 0.699)	0.4285
Radial DML (ms)	−0.146 (2.2750)	−0.567 (3.5035)	−0.292 (−1.022, 0.438)	0.4327

**Quality of life**				

EQ-5D total score	−0.2 (2.33)	−0.5 (2.01)	−0.363 (−0.962, 0.236)	0.2346
EQ-VAS	3.6 (16.63)	4.3 (14.45)	1.707 (−1.749, 5.163)	0.3331

Group difference in the table represents least square means and 95% CI.

a Response rate 1 is defined as the proportion of patients with ONLS improvement (at least 1 point) from baseline at the end of treatment.

b Response rate 2 is defined as the proportion of patients with no worsening of ONLS from baseline at the end of treatment.

The remaining secondary efficacy endpoints consistently yielded numerically higher point estimates for the PXT3003 group versus the placebo group (Table 2; Figure S1). These measures included the ONLS arm sub score, CMTNS-v2 total score, time 10m walk test (10MWT), hand grip strength, and EuroQol visual analogue scale (EQ-VAS) score, though none of these changes attained statistical significance. For instance, the mean change in the CMTNS-v2 total score was −1.0 in the PXT3003 group, compared to −0.6 in the placebo group. Sub-item changes for the CMTNS-v2 are listed in Table S2. No significant disparity was found in the four electrophysiological parameters between PXT3003 and placebo group (ulnar nerve compound motor action potential [CMAP], radial nerve sensory nerve action potential [SNAP], ulnar nerve distal motor latency [DML], and radial nerve DML).

### Post hoc outcomes

**Figure 3:**
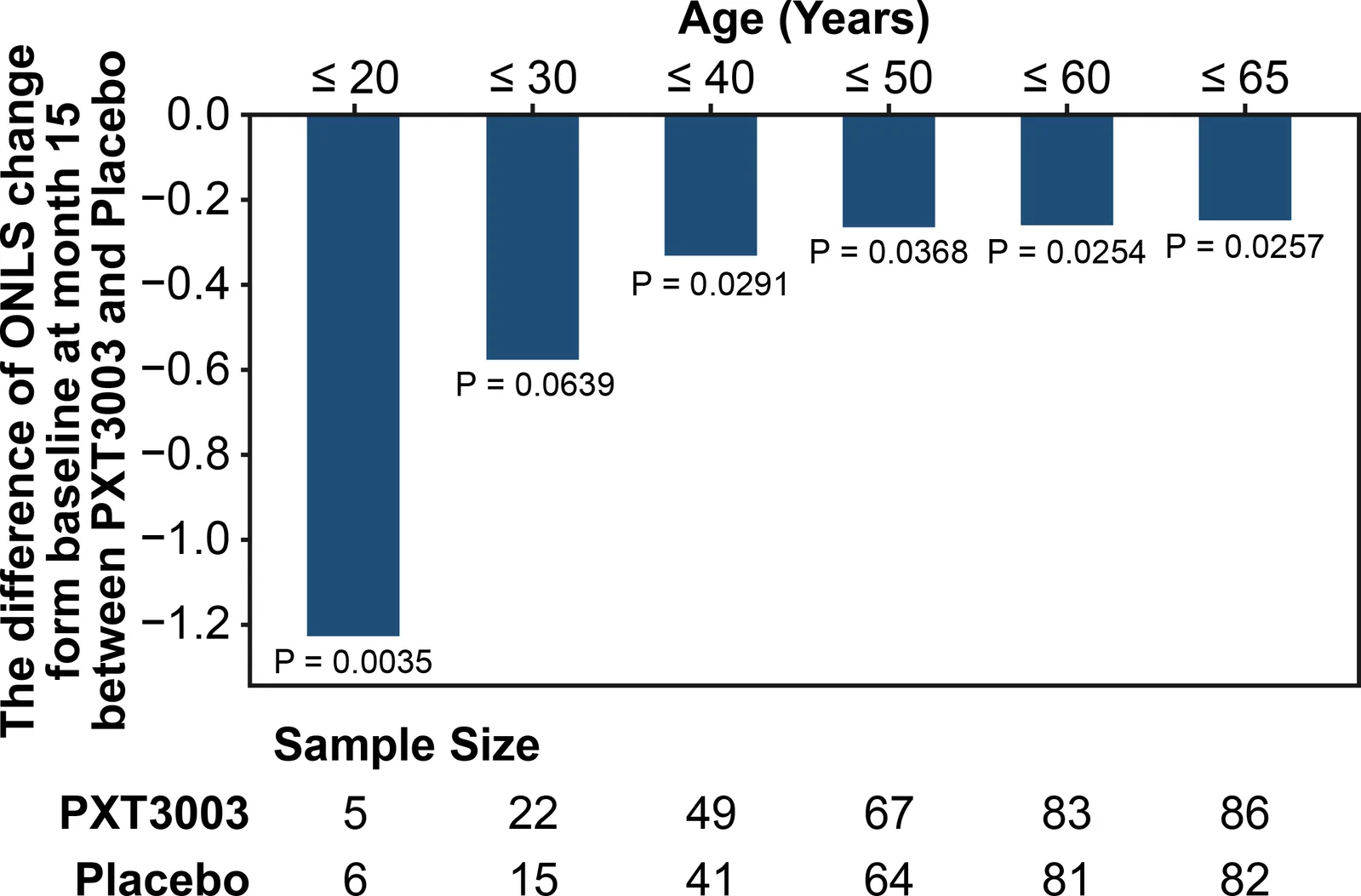
Changes in ONLS difference between PXT3003 and placebo groups (in different age ranges) The *p* values for between‑group comparisons were derived from analysis of covariance (ANCOVA) with baseline ONLS score as a covariate.

Therapeutic efficacy is moderately correlated with age. The younger the patients, the greater the between-group difference in efficacy between PXT3003 and the placebo group, indicating that earlier intervention with PXT3003 yields better therapeutic outcomes (Figure 3). Patients with moderate severity (CMTNS-V2 > 10) showed significant improvement, while the intergroup difference in ONLS was greater (−0.297 vs. −0.127, Table S3). All subgroup analyses were post hoc and exploratory, intended for hypothesis generation rather than confirmatory testing. Therefore, no adjustment for multiple comparisons was applied.

### Safety

Adverse events (AEs), all of which were mild (grade 1 or 2), were reported in 29.5% and 27.3% of patients in the PXT3003 and placebo groups, respectively. There were no serious AEs (SAEs) related to the study drug. Ten SAEs occurred, with five cases (5.7%) in each group (Table S4). The most frequent AEs in the PXT3003 group were dizziness (11.4%) and nausea (5.7%) (Table S5). No clinically significant changes were observed in hematology, blood biochemistry, urinalysis, 12-lead electrocardiography, or vital signs.

## Discussion

PXT3003 was assessed for efficacy and safety in Chinese patients with CMT1A via this 15-month randomized controlled clinical trial. The primary analysis revealed a significantly greater improvement in ONLS scores from baseline to month 15 for the PXT3003 group, showing a between-group difference of −0.249 points. The observed improvement was more pronounced in the lower extremities, evidenced by a significant change in the ONLS leg sub-score and dorsiflexion strength, by standard dynamometer (N). The ONLS measures the perceived ability of the patient to move and enjoy a normal life, and was recommended as a core disability scale for peripheral nerve studies in the 136^th^ European Neuromuscular Committee meeting.^10^^,^^11^ The changes in ONLS from baseline were −0.268 for the PXT3003 group and 0.013 for the placebo group, whereas a more pronounced treatment effect in the PXT3003 group emerged after 12 months—potentially due to drug accumulation—supporting the need for an extended treatment and follow-up period. Natural history studies and placebo-controlled trials indicate that ONLS progression in mild-to-moderate CMT1A is remarkably slow, with annual changes ranging from 0.01 to 0.2 points.^12^ Although a validated minimal clinically important difference (MCID) for ONLS in CMT1A has not been established, this 0.249-point difference over 15 months—which represents arrest of progression and modest functional gain—carries greater clinical significance. In this slowly progressive disease with no approved therapy, halting functional decline is as meaningful as achieving improvement. PXT3003 remains the only positive phase III investigational drug for CMT1A to date. Moreover, higher rates of improved/stable ONLS scores and significant lower limb functional gains suggest reduced fall risk—a key outcome for patient safety and quality of life.

The PXT3003 group also reported a numerically higher response rate (≥1-point ONLS improvement: 26.7% vs. 17.1%, *p* = 0.141), although it did not reach statistical significance. Several factors explain this finding. First, the study was powered for the continuous primary endpoint (ONLS change) and not for dichotomous responder rates. Detecting an approximate 10% absolute difference in response rates would require a substantially larger sample size. Second, the ≥1-point threshold may be relatively stringent for a 15-month trial in mild-to-moderate CMT1A, given the annual progression rate of only 0.1–0.2 points. Third, the significant continuous analysis indicates a modest but consistent favorable shift across the entire ONLS distribution, rather than an effect confined to a small subset. Notably, when response is redefined as improvement or stabilization (change ≥0), 89.5% of patients in group PXT3003 experienced no disease worsening, reflecting that preventing functional decline—a clinically meaningful outcome in an incurable progressive disease—may be the primary benefit of PXT3003.

While the effect size on ONLS change in our trial is comparable to that reported in the phase II (NCT01401257) and the pivotal phase III (NCT02579759) studies of PXT3003,^8^^,^^9^ it is worth noting that the recently completed confirmatory phase III trial (NCT04762758) did not achieve its primary efficacy endpoint. Several factors may explain this discrepancy. Previous positive trials, including ours, enrolled populations with relatively homogeneous genetic backgrounds, whereas NCT04762758 was a large multinational study (∼350 subjects across ∼52 global sites). Differences in genetic background (e.g., PMP22 duplication size, modifier genes), disease severity distribution, and environmental factors may influence treatment response and contribute to divergent outcomes. It is also more difficult to conduct quality control across 52 global sites versus 25 unified Chinese sites; and drug formulation—our use of an optimized liquid formulation to avoid crystallization issues—may play a role. Individual-patient-data meta-analyses are needed to clarify which subgroups benefit from PXT3003; unfortunately, these data are not currently accessible.

The subgroup analysis of this study reveals a significant and clinically insightful trend: younger patients exhibited a more pronounced therapeutic response to PXT3003 (Figure 3). This finding aligns closely with the natural history of CMT1A as a chronic, progressive neurodegenerative disorder. In the early stages of the disease, dysmyelination and impaired myelin stability are the primary pathophysiological events,^7^ whereas axonal loss and irreversible neuromuscular structural changes are relatively less severe. PXT3003, a drug designed to reduce the overexpression of the PMP22 gene and thereby promote healthier myelin development and stability, appears to have a critical therapeutic window. The nervous system in younger patients retains greater plasticity and regenerative potential. The drug may more effectively correct the ongoing pathological process during development and even create an opportunity for more normal myelination, leading to greater functional clinical benefits. In contrast, in advanced disease stages, extensive axonal degeneration and subsequent muscle atrophy likely become dominant. While intervention targeting myelin remains valuable at this point, the window for reversing structural damage is considerably narrower. This result strongly supports a clinical strategy of “early diagnosis and early intervention” for patients with CMT1A. Future research should focus on further validating the long-term efficacy and safety of PXT3003 in pediatric and adolescent populations and exploring the potential value of moving the treatment window forward to the presymptomatic or earliest clinical stage of the disease.

Additionally, the remaining secondary outcome measures, including the CMTNS-v2 score, 10MWT, and quantitative muscle testing (QMT) results, consistently showed a positive trend of improvement, although the change did not reach statistical significance. A previous longitudinal study reported an estimated annual increase of 0.686 points in the CMTNS total score, reflecting disease progression.^12^ In contrast, our study showed mean changes in the CMTNS-v2 total score of −0.665 in the placebo group and −0.972 in the PXT3003 group. The unexpected decrease in the CMTNS-v2 score in the placebo group likely influenced the statistical results; however, this is not an isolated issue. By reviewing of other placebo groups in clinical trials, we found that annual mean changes in the CMTNS-v2 total score ranged from −0.46 to 0.5 (Figure S2).^13^^,^^14^^,^^15^^,^^16^ Specifically, Pareyson et al. reported a worsening of 0.23 points per year in their placebo group (slower than the 0.5 points per year observed in the French trial^17^), while a US-based trial reported a placebo group improvement of −0.46 points per year.^15^ These inconsistent findings suggest that CMTNS-v2 change in placebo groups does not reliably reflect the true natural history of CMT1A. The slowly progressive nature of CMT1A and other factors, such as spontaneous fluctuations in disease progression or placebo effects, learning effects, and the limited sensitivity of the CMTNS-v2 over short trial durations, may contribute to these variations. These limitations suggest that the CMTNS-v2 may not be an optimal primary endpoint for 15-month CMT1A trials. Newer tools, such as the CMT functional outcome measure (CMT-FOM)^18^ and the CMT Health Index,^19^ may offer greater sensitivity for future studies. Similarly, electrophysiological studies are also not very sensitive in CMT1A. Motor nerve conduction velocity (MCV) has shown no correlation with age or severity, even in young children during the initial stages of the disease. Although a high baseline MCV value may serve as an indicator of a favorable prognosis and enhanced response to treatment,^17^ the severity of CMT1A correlated more strongly with axonal degeneration secondary to demyelination. In our study, a small, non-significant increase in CMAP amplitude was observed, potentially indicating remyelination and an increased number of axons.^7^ The lack of statistical significance in most secondary endpoints is more likely attributable to insufficient statistical power, the limited sensitivity of the measures, and the short treatment duration relative to the slowly progressive nature of CMT1A, rather than an inadequate dose.

It is hypothesized that PXT3003 could be effective because each component drug acts via the cAMP-dependent silencer element to negatively regulate *PMP22* transcription in Schwann cells, ultimately exhibiting synergistic effects.^5^^,^^6^ On the molecular level of the sciatic nerve in rat models of CMT1A, PXT3003 reduced *PMP22* mRNA overexpression and improved the misbalanced downstream PI3K-AKT/MEK-ERK signaling pathway.^7^ A reduction of nearly 30%–50% in PMP22 mRNA levels of PXT3003 is considered optimal because excessive downregulation of PMP22 mRNA may lead to another disease: hereditary neuropathy with liability to pressure palsies (HNPP).^5^ Several preclinical gene therapies aimed at downregulating PMP22 mRNA are currently ongoing; however, determining the optimal percentage of downregulation and a better delivery system to peripheral nerves require further investigation.^19^ Nevertheless, PXT3003, the first small-molecule therapy for CMT1A, marginally improved ONLS scores and showed promising trends in secondary outcome measures in a large, multi-center, placebo-controlled phase III study.^9^ These inspiring findings warrant further investigation in clinical trials.

This study further confirmed the safety and tolerability of PXT3003. In the previous phase II^8^ and phase III^9^ clinical studies of PXT3003, a good dose-response relationship was observed, which helped determine the dosing regimen for this study. In the earlier phase III study, stability issues leading to crystal formation were encountered with the high-dose formulation although the safety of the drug was not affected. To address this issue, a low-concentration formulation at double the volume was used in the current study, which ensured both adequate drug exposure and formulation stability. To further ensure the tolerability of the participants, the dosing regimen was designed such that a 5-mL dose per administration was used in the first 2 weeks, which was then increased to 10 mL per administration.

PXT3003 significantly improved ONLS scores, leg function, and dorsiflexion strength over a period of 15 months, potentially leading to substantial quality-of-life enhancements for the patients. These findings highlight the promising therapeutic effects of PXT3003 against CMT1A, offering a potential solution to address the critical treatment gap for the most common genotype of CMT disease.

### Limitations of the study

Several mechanistic limitations should be acknowledged. First, although PXT3003 is designed to downregulate PMP22 overexpression via the cAMP-dependent silencer element, we did not measure PMP22 expression levels in this trial. Direct target-engagement biomarkers—such as PMP22 mRNA levels in skin biopsy-derived Schwann cells or sural nerve tissue—were not assessed. Second, our study lacks sensitive molecular or imaging endpoints that might detect early or subclinical treatment effects. Similarly, molecular biomarkers (e.g., neurofilament light chain as a marker of axonal damage) were not measured. Future proof-of-concept or longitudinal studies should incorporate more sensitive functional tools, such as the CMT-FOM^18^ and self-reported CMT health index,^19^ and more objective biomarkers, such as intramuscular fat fraction of calf muscle, mRNA from skin biopsies using nanostring,^20^^,^^21^^,^^22^^,^^23^ and fluid biomarkers to better characterize the relationship between target engagement and structural preservation.

Our current findings demonstrate **s**hort-term functional improvement and stabilization over 15 months, but confirmation of a genuine disease-modifying effect will require longer term follow-up. In the subsequent stage, we may also launch a continuous open-label extension study to further elucidate the long-term sustainability of the clinical benefits identified in this research.

## Resource availability

### Lead contact

All inquiries regarding trial resources, study materials, raw clinical datasets, and supplementary supporting documents shall be addressed to and fulfilled by the corresponding lead contact, Dongsheng Fan (dsfan@sina.com).

### Materials availability

No novel proprietary laboratory reagents or unique biological specimens were generated in this multicenter phase III clinical trial of PXT3003 for CMT1A.

### Data and code availability

• All raw and processed clinical datasets generated in this study have been deposited in ScienceDB with a persistent dataset https://doi.org/10.57760/sciencedb.43310.
• All original code has been deposited at ScienceDB and is publicly available at https://doi.org/10.57760/sciencedb.43310 as of the date of publication.
• Any additional information required to reanalyze the data reported in this paper is available from the lead contact upon request.

## Acknowledgments

This trial was supported by grants from the Genenet Pharmaceutical Co., Ltd; the 10.13039/501100001809National Natural Science Foundation of China, (grant nos. 81873784, 82071426, and 82171172); the Beijing Municipal Natural Science Foundation (grant no. 7242170); the Clinical Cohort Construction Program of Peking University Third Hospital (grant nos. BYSYDL2019002, BYSYDL2024008, BYSYZD2021004, and BYSYZD2022009); and the Hunan Province Key Research and Development Program (grant no. 2024JK2116). We gratefully acknowledge the participation of all patients and investigators in this study.

## Author contributions

D.F. and R. Zhang led the study. D.F., X.L., and R. Zhang contributed to the study design. X.L., W.C., Y.D., B.Y., N.W., S.W., J.L., Z.L., Q.F., Z.-Y.W., L.Y., J.J., H.J., Y.L. Liu, M.M., L.W., J.T., X. Yao, Y.S., X. Yu, Y.M. Liu, D.Z., Y. Xu, Y.M. Xu, and R. Zhu participated in data acquisition. D.F., X.L., and R. Zhang analyzed data. X.L. wrote the manuscript. C.S., J.H., and H.L. verified the data. All authors had full access to all data in the study and had final responsibility for the decision to submit the manuscript for publication.

## Declaration of interests

The authors declare no competing interests.

## STAR★Methods

### Key resources table

**Table:** 

REAGENT or RESOURCE	SOURCE	IDENTIFIER
**Other**		

Raw clinical trial dataset	ScienceDB	https://doi.org/10.57760/sciencedb.43310
Statistical analysis software (SAS)	SAS Institute	SAS 9.4
Sample size calculation software (PASS)	NCSS, LLC (Kaysville, UT, USA)	Version 15.0
Randomization system	central Interactive Web Response System(IWRS)	N/A
Independent Data Monitoring Committee (IDMC)	Independent third-party experts	N/A
Clinical trial registration	ClinicalTrials.gov	NCT05092841
Ethics approval	the ethics committees of the Peking University Third Hospital	Approval No. (2021) 094-02; Approval Date: June.22,2021
Ethics approval	the Third Xiangya Hospital of Central South University	Approval No. 21096; Approval Date: June 7, 2021

The study was conducted in accordance with the International Council on Harmonization Good Clinical Practice guidelines and the Declaration of Helsinki.

### Experimental model and study participant details

This multicenter, randomized, double-blind, placebo-controlled phase III clinical trial was conducted at 25 sites in China. This trial enrolled patients aged 16 to 65 years with mild-to-moderate CMT1A, defined as a the Charcot-Marie-Tooth Neuropathy Score version 2(CMTNS-v2) score between 2 and 18, and a confirmed genetic diagnosis of *PMP22* duplication determined by multiplex ligation-dependent probe amplification(MLPA). In addition, patients were required to have at least dorsiflexor muscle weakness and an ulnar nerve motor conduction velocity exceeding 15 m/s. Key exclusion criteria encompassed allergies or contradindications to the study drug, other neuropathies, neurological conditions that might interfere with treatment evaluation, an Overall Neuropathy Limitations Scale (ONLS) score of 0, and recent or planned limb surgery within 6 months of randomization. All participants were required to use effective contraception. Complete eligibility criteria are provided in the study protocol (Data S2).

The study protocol was approved by the ethics committees of the Peking University Third Hospital (2021.06.22), the Third Xiangya Hospital of Central South University(June 7, 2021), and independent ethics committees at each study site. All participants provided written informed consent before screening. This trial is registered with ClinicalTrials.gov, NCT05092841.

The overall mean (±SD) treatment adherence rate was 92.35% (±13.731%) for the PXT3003 group and 92.04% (±15.733%) for the placebo group.

Subgroup analyses stratified by sex and gender were not pre-specified in the trial statistical analysis plan; thus, we cannot quantitatively assess the independent influence of sex or gender on treatment-related functional outcomes. This represents a recognized limitation of the present study.

### Method details

The trial comprised two phases: a 30-day screening period and a 450-day treatment period. Following the provision of informed consent, participants entered the screening phase, which commencedwith the confirmation of PMP22 gene duplication for CMT1A diagnosis.

Comprehensive subsequent assessments encompassed demographics; vital signs; physical examination findings; lifestyle; medical history; and laboratory testss. Participants were also assessed with the ONLS, the CMTNS-v2, and electrophysiological tests. ONLS has been derived from the Overall Disability Sum Score (ODSS) to measure limitations of activities in the upper limbs (0–5 points) and the lower limbs (0–7 points).^9^ The total score ranges from 0 (no disability) to 12 (maximum disability). A second, refined version of the Charcot-Marie-Tooth Neuropathy Score (CMTNS) scale, the CMTNS-v2, was introduced by Reilly et al.^10^ This 36-point assessment tool evaluates nine domains, categorized into symptoms (sensory symptoms, motor symptoms of the arms, and motor symptoms of the legs), signs (pinprick sensitivity, vibration sense, and muscle strength in the upper and lower extremities), and electrophysiological parameters (ulnar compound muscle action potential [CMAP] and radial sensory action potential). Higher scores indicate worsening function. Additionally, the time 10-m walk test (10MWT), 9-hole peg test (9HPT), quantitative muscle testing (QMT, grip strength and foot dorsiflexion), electrophysiological studies, and quality-of-life assessments (5-Level EuroQol [EQ-5D] and EuroQol Visual Analogue Scale [EQ-VAS]) were also conducted.

Following successful screening, the participants were randomized to receive either PXT3003 or placebo for 15 months via the IWRS system. The study medication was administered orally twice daily, with breakfast and dinner. The initial dose was 5 mL per dose for 2 weeks, followed by an increase to 10 mL per dose. Five on-site visits occurred every 3 months during the treatment phase. Assessments at months 6, 12, and 15 included the ONLS, CMTNS-v2, 10MWT, 9HPT, QMT, electrophysiological studies, and EQ-5D evaluation. Vital signs, physical examinations, laboratory tests, adverse events (AEs), and concomitant medications were recorded at each visit.

#### Sample size

A 1:1 randomization ratio was employed. The difference in ONLS score improvement from baseline between the treatment and the placebo group was assumed to be 0.3, with a standard deviation of 0.6. By controlling for a two-sided type I error rate of 0.05, the study provided at least 80% power to detect the superiority of the treatment group compared with the placebo group. Considering a dropout rate of 20%, each group needed 88 participants, for a total sample size of 176. Sample size calculations were performed using PASS software (version 15.0).

#### Randomization and masking

Eligible participants were randomly assigned in a 1:1 ratio to receive PXT3003 or placebo. A central Interactive Web Response System (IWRS) was used for randomization. Randomization lists for participants and medications were generated using SAS software by personnel not involved in the clinical operations and statistical analysis. Blinded records were created by individuals with access to randomization codes, which were securely maintained along with the corresponding SAS programs. All outcome assessors, including personnel responsible for ONLS scoring, remained blinded to treatment allocation throughout the entire trial.

#### Outcomes

The primary endpoint was the difference in the ONLS score at the baseline and that at 15 months. Eight secondary outcomes were evaluated at the 15 months, including the treatment response rate to PXT3003, the ONLS arm and leg subscale scores, CMTNS-v2 score, 10MWT, 9HPT, QMT, and electrophysiological study results, and EQ-5D and EQ-VAS scores. The safety assessment focused on the incidence of AEs, changes in vital signs, laboratory test results, and electrocardiography readings.

### Quantification and statistical analysis

The full analysis set, comprising all randomized participants with at least one post-baseline ONLS score, was used for efficacy analysis. Safety evaluations were based on the safety analysis set, including all randomized participants who received at least one dose of the study drug.

The primary efficacy and continuous secondary endpoints were analyzed using an analysis of covariance (ANCOVA) model, with the baseline value as a covariate and the treatment group as a fixed effect. Treatment group effects, along with the associated 95% confidence intervals (CIs) and *p*-values, were reported. For secondary endpoints involving proportions, 95% CIs for response rates were calculated using the Clopper-Pearson method, with group comparisons performed using the chi-squared test. Descriptive statistics were applied to summarize EQ-5D index scores, EQ-VAS scores, and the changes in these scores at each visit from baseline scores. Safety analysis descriptively reported the proportion of participants experiencing at least one AE.

Missing values for primary efficacy endpoints were imputed using predicted values from a linear model based on placebo group data. Final estimates were derived from multiple imputation.

An independent data monitoring committee would oversee the study and conduct an interim analysis. This analysis would be conducted after 88 participants complete the 15-month ONLS assessment to determine if the trial should continue. A Lan-Demets algorithm with an O’Brien-Fleming alpha-spending function would be used within a group sequential design to maintain an overall type I error rate of 0.05. If the interim analysis *p*-value is ≤ 0.003, the trial would be stopped early owing to significant efficacy. Otherwise, the trial would proceed, and the sample size would be reassessed using the promising zone method based on conditional power. The final analysis would be conducted upon the completion of all participant assessments, with a significance level of 0.049.

Sensitivity and supplementary analyses of the primary endpoint included using a regression-based approach for handling missing data, employing different strategies to handle intercurrent events, performing the primary analysis in the population without major protocol deviations affecting the primary efficacy endpoint, and applying a mixed-effect model for repeated measures (MMRM) to the unimputed data without considering the impact of intercurrent events (Data S1).

All statistical analyses were performed using SAS software (version 9.4 or later).

#### Added value of this study

This multicentre, randomised, double-blind, placebo-controlled phase 3 trial provides the first robust evidence for the efficacy and safety of PXT3003 in a large Chinese CMT1A population. By using the Overall Neuropathy Limitations Scale (ONLS), a validated disability-focused outcome recommended for peripheral neuropathy trials, this study demonstrates a statistically significant and clinically relevant improvement in functional disability over 15 months. The trial also confirms treatment benefits in lower-limb function and ankle dorsiflexion strength, while showing good tolerability with no treatment-related serious adverse events. Subgroup analyses suggest that younger patients and those with moderate disease severity may derive greater benefit, offering insight into optimal treatment timing.

#### Implications of all the available evidence

The findings of this study reinforce and extend the evidence that PXT3003 is a safe and effective disease-modifying treatment for CMT1A. The significant improvement in functional disability, particularly in lower limb strength, suggests that PXT3003 can alleviate disease burden. The correlation between younger age and better therapeutic outcomes supports a clinical strategy of early diagnosis and intervention to maximize myelin plasticity and prevent irreversible axonal damage. Together with previous international data, these results support the potential of PXT3003 to fill the unmet medical need for CMT1A treatment and highlight the necessity for future studies in pediatric populations.

### Additional resources

Clinical trial registration page for this phase III clinical study: https://clinicaltrials.gov/study/NCT05092841.

Public repository hosting raw and processed clinical datasets from this trial: https://doi.org/10.57760/sciencedb.43310.

## Supplemental information

Document S1. Figures S1and S2 and Tables S1–S5

Data S1. Detailed statistical analysis plan of the phase III clinical trial

Data S2. Full clinical trial protocol of PXT3003 in CMT1A patients

Data S3. CONSORT 2010 checklist for phase III clinical trial

## References

[1] Skre H. (1974). Genetic and clinical aspects of Charcot-Marie-Tooth's disease. Clin. Genet..

[2] Wise C.A., Garcia C.A., Davis S.N., Heju Z., Pentao L., Patel P.I., Lupski J.R. (1993). Molecular analyses of unrelated Charcot-Marie-Tooth (CMT) disease patients suggest a high frequency of the CMT1A duplication. Am. J. Hum. Genet..

[3] Pareyson D., Marchesi C. (2009). Diagnosis, natural history, and management of Charcot-Marie-Tooth disease. Lancet Neurol..

[4] Beloribi-Djefaflia S., Attarian S. (2023). Treatment of Charcot-Marie-Tooth neuropathies. Rev. Neurol. (Paris).

[5] Chumakov I., Milet A., Cholet N., Primas G., Boucard A., Pereira Y., Graudens E., Mandel J., Laffaire J., Foucquier J. (2014). Polytherapy with a combination of three repurposed drugs (PXT3003) down-regulates Pmp22 over-expression and improves myelination, axonal and functional parameters in models of CMT1A neuropathy. Orphanet J. Rare Dis..

[6] Hajj R., Prukop T., Wernick S., Ewers D., Bureau A., Cholet N., Laffaire J., Nave K.-A., Cohen D., Sereda M. (2019). Baclofen, naltrexone and sorbitol all contribute to the efficacy of PXT3003 in CMT1A rats. EMJ Neurol..

[7] Prukop T., Stenzel J., Wernick S., Kungl T., Mroczek M., Adam J., Ewers D., Nabirotchkin S., Nave K.-A., Hajj R. (2019). Early short-term PXT3003 combinational therapy delays disease onset in a transgenic rat model of Charcot-Marie-Tooth disease 1A (CMT1A). PLoS One.

[8] Attarian S., Vallat J.-M., Magy L., Funalot B., Gonnaud P.-M., Lacour A., Péréon Y., Dubourg O., Pouget J., Micallef J. (2014). An exploratory randomised double-blind and placebo-controlled phase 2 study of a combination of baclofen, naltrexone and sorbitol (PXT3003) in patients with Charcot-Marie-Tooth disease type 1A. Orphanet J. Rare Dis..

[9] Attarian S., Young P., Brannagan T.H., Adams D., Van Damme P., Thomas F.P., Casanovas C., Kafaie J., Tard C., Walter M.C. (2021). A double-blind, placebo-controlled, randomized trial of PXT3003 for the treatment of Charcot-Marie-Tooth type 1A. Orphanet J. Rare Dis..

[10] Graham R.C., Hughes R.A.C. (2006). A modified peripheral neuropathy scale: the Overall Neuropathy Limitations Scale. J. Neurol. Neurosurg. Psychiatry.

[11] Reilly M.M., de Jonghe P., Pareyson D. (2006). 136th ENMC International Workshop: Charcot-Marie-Tooth disease type 1A (CMT1A) 8–10 April 2005, Naarden, The Netherlands. Neuromuscul. Disord..

[12] Mandel J., Bertrand V., Lehert P., Attarian S., Magy L., Micallef J., Chumakov I., Scart-Grès C., Guedj M., Cohen D. (2015). A meta-analysis of randomized double-blind clinical trials in CMT1A to assess the change from baseline in CMTNS and ONLS scales after one year of treatment. Orphanet J. Rare Dis..

[13] Solari A., Laurà M., Salsano E., Radice D., Pareyson D., CMT-TRIAAL Study Group (2008). Reliability of clinical outcome measures in Charcot-Marie-Tooth disease. Neuromuscul. Disord..

[14] Shy M.E., Chen L., Swan E.R., Taube R., Krajewski K.M., Herrmann D., Lewis R.A., McDermott M.P. (2008). Neuropathy progression in Charcot-Marie-Tooth disease type 1A. Neurology.

[15] Pareyson D., Reilly M.M., Schenone A., Fabrizi G.M., Cavallaro T., Santoro L., Vita G., Quattrone A., Padua L., Gemignani F. (2011). Ascorbic acid in Charcot-Marie-Tooth disease type 1A (CMT-TRIAAL and CMT-TRAUK): a double-blind randomised trial. Lancet Neurol..

[16] Lewis R.A., McDermott M.P., Herrmann D.N., Hoke A., Clawson L.L., Siskind C., Feely S.M.E., Miller L.J., Barohn R.J., Smith P. (2013). High-dosage ascorbic acid treatment in Charcot-Marie-Tooth disease type 1A: results of a randomized, double-masked, controlled trial. JAMA Neurol..

[17] Burns J., Ouvrier R.A., Yiu E.M., Joseph P.D., Kornberg A.J., Fahey M.C., Ryan M.M. (2009). Ascorbic acid for Charcot-Marie-Tooth disease type 1A in children: a randomised, double-blind, placebo-controlled, safety and efficacy trial. Lancet Neurol..

[18] Eichinger K., Burns J., Cornett K., Bacon C., Shepherd M.L., Mountain J., Sowden J., Shy R., Shy M.E., Herrmann D.N. (2018). The Charcot-Marie-Tooth Functional Outcome Measure (CMT-FOM). Neurology.

[19] Johnson N.E., Heatwole C., Creigh P., McDermott M.P., Dilek N., Hung M., Bounsanga J., Tang W., Shy M.E., Herrmann D.N. (2018). The Charcot-Marie-Tooth Health Index: Evaluation of a Patient-Reported Outcome. Ann. Neurol..

[20] Svaren J., Moran J.J., Wu X., Zuccarino R., Bacon C., Bai Y., Ramesh R., Gutmann L., Anderson D.M., Pavelec D., Shy M.E. (2019). Schwann cell transcript biomarkers for hereditary neuropathy skin biopsies. Ann. Neurol..

[21] O'Donnell L.F., Pipis M., Thornton J.S., Kanber B., Wastling S., McDowell A., Zafeiropoulos N., Laura M., Skorupinska M., Record C.J. (2024). Quantitative MRI outcome measures in CMT1A using automated lower limb muscle segmentation. J. Neurol. Neurosurg. Psychiatry.

[22] Sun X., Liu X., Zhao Q., Zhang L., Yuan H. (2023). Quantified fat fraction as biomarker assessing disease severity in rare Charcot-Marie-Tooth subtypes. Front. Neurol..

[23] Stavrou M., Kleopa K.A. (2023). CMT1A current gene therapy approaches and promising biomarkers. Neural Regen. Res..

