# Supplementary material for: A phase III clinical trial of PXT3003 in Chinese patients with Charcot-Marie-Tooth disease type 1A
Source: iScience. 2026 Aug 26;29(9):117284. doi: 10.1016/j.isci.2026.117284 (PMC13544322; doi:10.1016/j.isci.2026.117284)
Supplement: Supplementary file 1 [file mmc1.pdf]

iScience, Volume *29*

Supplemental information

A phase III clinical trial of PXT3003 in Chinese patients with Charcot-Marie-Tooth disease type 1A

Xiaoxuan Liu, Wanqian Cao, Hongli Li, Chen Sun, Jingzheng Hao, Yuwei Da, Baoyu Yuan, Ning Wang, Shan Wu, Jun Liu, Zuneng Lu, Qi Fang, Zhi-Ying Wu, Liying Yuan, Jiaoting Jin, Haishan Jiang, Yaling Liu, Mingming Ma, Ling Wei, Jianglong Tu, Xiaoli Yao, Yuan Sun, Xuefan Yu, Yiming Liu, Di Zhong, Yun Xu, Yuming Xu, Runxiu Zhu, Dongsheng Fan, and Ruxu Zhang

Supplementary Figure S1. Summary of the efficacy analysis: PXT3003 versus placebo

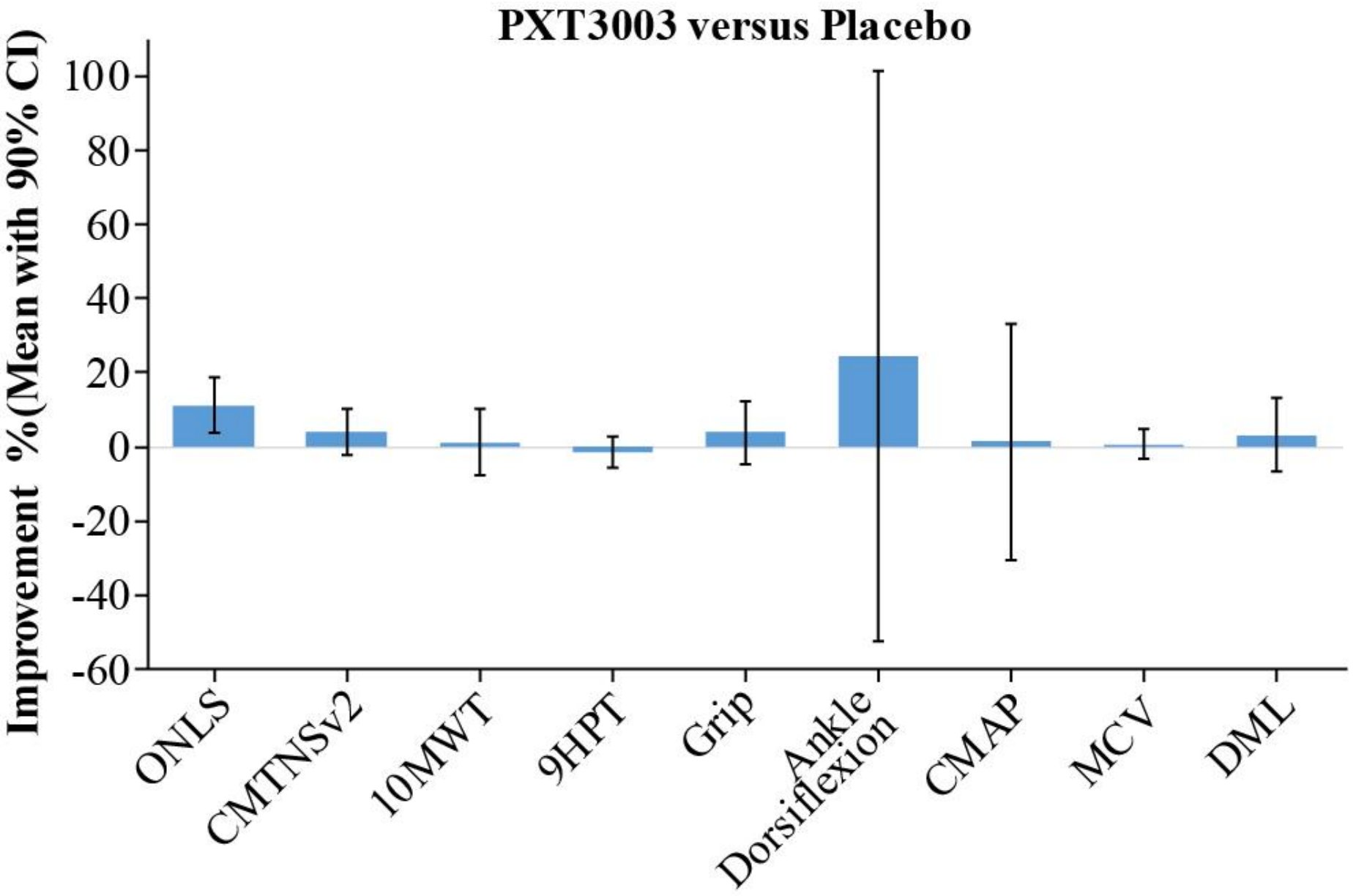

Least squares mean percentages of relative improvement after 15 months along with 90% CI in all efficacy outcomes after 15 months for PXT3003 groups versus Placebo.

ONLS, Overall Neuropathy Limitations Scale; CMTNSv2, Charcot-Marie-Tooth Neuropathy Score version 2; 10MWT, 10-Meter Walk Test; 9HPT, 9-Hole Peg Test; CMAP, Compound Motor Action Potential; MCV, Motor Conduction Velocity; DML, Distal Motor Latency.

Supplementary Figure S2. Changes in the CMTNS-v2 score

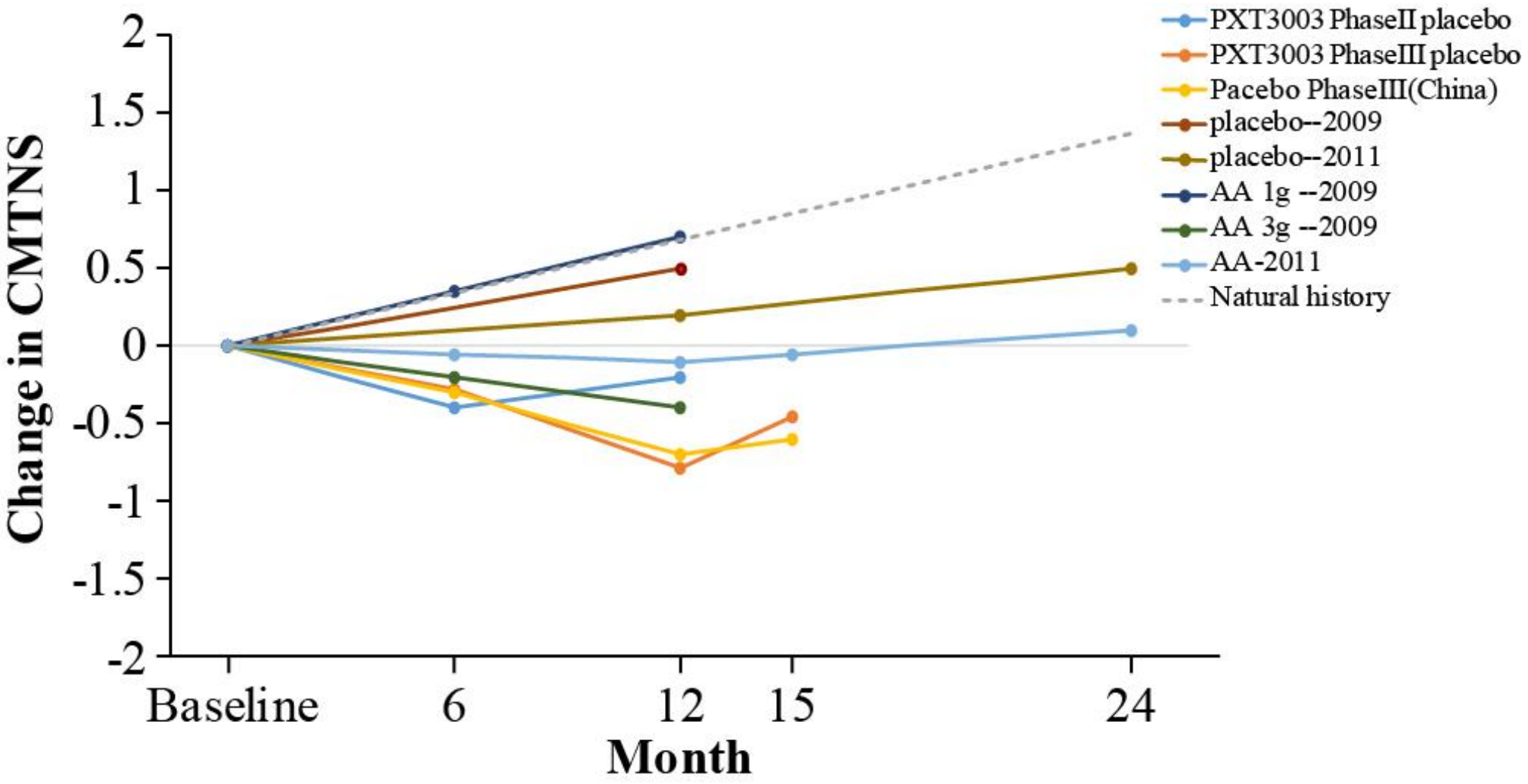

Changes in the Charcot-Marie-Tooth Neuropathy Score (CMTNS) over the 2-year study.
Adjusted mean changes in the CMTNS over 2 years are shown for the ascorbic acid (AA) group and placebo group. Placebo-2009/2011 and all AA groups are derived from two independent clinical trials of ascorbic acid for CMT1A.
CMTNS was used for Natural history, AA, and phase II PXT3003 trial, and placebo; CMTNS-v2 was used for the phase III PXT3003 trial.

Supplementary Table S1. Analysis of the ONLS Score Relative from Baseline to 15 months

Analysis	PXT3003 Group	Placebo Group	Difference in Least Squares Means and 95% CI	P value
Sensitivity Analysis	−0·272 (−0·425, −0·119)	−0·020 (−0·178, 0·137)	−0·252 (−0·472, −0·032)	0·0247
Supplementary Analysis 1	−0·227 (−0·385, −0·068)	0·007 (−0·159, 0·174)	−0·234 (−0·462, −0·005)	0·0448
Supplementary Analysis 2	−0·278 (−0·439, −0·118)	0·010 (−0·157, 0·177)	−0·289 (−0·521, −0·056)	0·0152
Supplementary Analysis 3	−0·262 (−0·416, −0·108)	−0·018 (−0·177, 0·141)	−0·244 (−0·466, −0·023)	0·0305
Supplementary Analysis 4	−0·271(−0·422, −0·119)	−0·020(−0·177, 0·136)	−0·250(−0·468, −0·032)	0·0243

Least Squares Mean and 95% CI
The sensitivity analysis included all randomized participants for whom at least one post-baseline ONLS score was available. Under the missing-at-random(MAR) assumption with a monotone missing pattern, missing data were imputed using a regression-based method, with multiple imputation and ANCOVA.
Supplementary analysis 1 used a hypothetical strategy, assuming that the efficacy in participants with concomitant events was similar to the observed data from the control group. The ONLS score at month 15 would be imputed based on a copy reference method.
Supplementary analysis 2 included all participants for whom there were no marked protocol deviations that could impact efficacy throughout the treatment period.
Supplementary analysis 3 was performed using a mixed-effects model for repeated measures under the MAR assumption, without considering the impact of concomitant events.
Supplementary analysis 4 was used to evaluate the robustness of the primary analysis results: A composite variable strategy was adopted. Data following the use of prohibited treatments that could affect efficacy assessment were handled using the Baseline Observation Carried Forward (BOCF) approach.

Supplementary Table S2. Change in CMTNS-v2 and CMTNS-v2 subscore from baseline to 15 months

	Placebo (N=82)	PXT3003 (N=86)	P value
**CMTNS-v2 total**			
Baseline	12·9 (3·12)	13·0 (2·89)	
15 months	12·3 (3·72)	11·9 (3·67)	
Change	−0·665 (−1·285, −0·046)	−0·972 (−1·564, −0·380)	0·4804
**CMTES**			
Baseline	7·2 (2·63)	7·2 (2·48)	
15 months	6·9 (3·08)	6·5 (2·89)	
Change	−0·365 (−0·905, 0·176)	**−0·737 (−1·259, −0·215)**	0·3283
**CMTNS-v2 subscores**			
Sensory symptoms			
Baseline	0·7 (0·94)	0·9 (1·10)	
15 months	0·6 (0·89)	0·5 (0·88)	
Change	−0·129 (−0·303, 0·046)	**−0·301 (−0·470, −0·132)**	0·1642
Motor symptoms,legs			
Baseline	1·0 (0·51)	1·0 (0·54)	
15 months	1·0 (0·60)	1·0 (0·66)	
Change	−0·069 (−0·161, 0·022)	−0·059 (−0·148, 0·029)	0·8821
Motor symptoms,arms			
Baseline	0·4 (0·57)	0·4 (0·52)	
15 months	0·5 (0·66)	0·3 (0·50)	
Change	0·052 (−0·057, 0·161)	**−0·072 (−0·177, 0·034)**	0·1077
Pin sensibility			
Baseline	1·2 (0·88)	1·0 (0·94)	
15 months	1·0 (0·88)	0·9 (0·76)	
Change	−0·136 (−0·304, 0·032)	**−0·192 (−0·353, −0·030)**	0·6428
Vibrations			
Baseline	1·1 (1·08)	1·1 (0·99)	

15 months	1·0 (1·05)	1·1 (0·99)	
Change	−0·064 (−0·263, 0·134)	0·026 (−0·166, 0·217)	0·5225
Strength,legs			
Baseline	1·8 (0·84)	1·8 (0·87)	
15 months	1·8 (0·87)	1·7 (1·01)	
Change	0·000 (−0·139, 0·139)	−0·054 (−0·188, 0·079)	0·5803
Strength,arms			
Baseline	0·9 (0·68)	0·9 (0·65)	
15 months	1·0 (0·58)	0·9 (0·66)	
Change	0·117 (0·008, 0·225)	−0·031 (−0·136, 0·073)	0·0542
Ulnar CMAP			
Baseline	1·9 (0·98)	1·9 (1·00)	
15 months	1·8 (1·10)	1·7 (1·14)	
Change	−0·153 (−0·327, 0·022)	−0·232 (−0·401, −0·064)	0·5170
Radial SNAP			
Baseline	3·8 (0·53)	3·8 (0·58)	
15 months	3·7 (0·60)	3·8 (0·52)	
Change	−0·128 (−0·243, −0·013)	−0·008 (−0·118, 0·103)	0·1396

CMTNSv2, Charcot-Marie-Tooth Neuropathy Score version 2.
CMAP, Compound Motor Action Potential; SNAP, Sensory Nerve Action Potential.

Supplementary Table S3. Changes in ONLS Across Baseline Severity

	PXT3003 (N)	Placebo (N)	Difference	P value
CMTNS-V2 ≤10	−0·127 (19)	−0·027 (18)	−0·099	0·6280
CMTNS-V2 >10	−0·297 (64)	−0·034 (67)	−0·263	0·0459

CMTNSv2, Charcot-Marie-Tooth Neuropathy Score version 2.

Change from baseline in ONLS at Month 15, stratified by baseline disease severity (CMTNS-V2 ≤10 and >10).

Supplementary Table S4. Summary of Adverse Events Post-Treatment

	Placebo Group (N=88)		PXT3003 (N=88)		
	Case	Number of Cases (%)	Case	Number of Cases (%)	P value
AEs	290	78 (88·6%)	357	82 (93·2%)	0·4325
AEs Related to Study Drug	46	24 (27·3%)	97	26 (29·5%)	0·8674
SAEs	5	5 (5·7%)	6	5 (5·7%)	>0·9999
SAEs Related to Study Drug	0	0	0	0	NA
Grade ≥3 TEAEs	6	5 (5·7%)	6	5 (5·7%)	>0·9999
Grade ≥3 TEAEs Related to Study Drug	0	0	0	0	NA
AEs Leading to Permanent Discontinuation	1	1 (1·1%)	0	0	>0·9999
AEs Related to Study Drug Leading to Permanent Discontinuation	0	0	0	0	NA
AEs Related to Study Drug Leading to Withdrawal from Study	0	0	0	0	NA
AEs Leading to Death	0	0	0	0	NA

AE, Adverse Event; TEAE, Treatment-Emergent Adverse Event. P-values were generated using Fisher's exact test.

Supplementary Table S5. TEAEs Related to PXT3003 in ≥2% of Patients

Preferred Term	Placebo Group (N=88)	PXT3003 Group (N=88)
Dizziness	2 (2·3%)	10 (11·4%)
Nausea	3 (3·4%)	5 (5·7%)
Hyperuricemia	0	4 (4·5%)
Diarrhea	1 (1·1%)	3 (3·4%)
Headache	0	3 (3·4%)
Back Pain	1 (1·1%)	3 (3·4%)
Abnormal Liver Function	5 (5·7%)	3 (3·4%)
Fatigue	2 (2·3%)	3 (3·4%)
Abdominal Discomfort	3 (3·4%)	2 (2·3%)
Upper Abdominal Pain	0	2 (2·3%)
Flatulence	0	2 (2·3%)
Somnolence	1 (1·1%)	2 (2·3%)
Elevated Alanine Aminotransferase	1 (1·1%)	2 (2·3%)
Elevated Aspartate Aminotransferase	1 (1·1%)	2 (2·3%)
Elevated γ-Glutamyl Transferase	0	2 (2·3%)
Palpitations	1 (1·1%)	2 (2··3%)
Urinary Tract Infection	0	2 (2·3%)
Insomnia	0	2 (2·3%)
Constipation	2 (2·3%)	1 (1·1%)
Limb Pain	2 (2·3%)	1 (1·1%)
Tremor	2 (2·3%)	0
