# Supplementary material for: A phase III clinical trial of PXT3003 in Chinese patients with Charcot-Marie-Tooth disease type 1A
Source: iScience. 2026 Aug 26;29(9):117284. doi: 10.1016/j.isci.2026.117284 (PMC13544322; doi:10.1016/j.isci.2026.117284)
Supplement: Supplementary file 2 [file mmc2.pdf]

Statistical Analysis Plan

Protocol No.: TSL-CM-PXT3003-III

Version No.: V1.0

Version Date: April 26, 2024

A Multicenter, Randomized, Double-blind, Placebo-controlled Phase III Clinical Trial to Assess the Efficacy and Safety of PXT3003 in Patients with Charcot-Marie-Tooth Disease Type 1A for 15 Months

Statistical Analysis Unit: Nanjing Aikeman Medical Information & Technology Co., Ltd

Sponsor: Genenet Pharmaceutical Co., Ltd

This document contains trade secrets and confidential business information. Disclosure is prohibited without prior authorization from Genenet Pharmaceutical Co., Ltd. and Nanjing Aikeman Medical Information & Technology Co., Ltd.

Signature Page

Prepared by:

DocuSigned by Shuang Hou
shuang.hou
I am the author of this document
April 26, 2024 | 4:55:03 PM CST
76E290E7712A426CBE958285B8F1C9BF

4-26-2024

Shuang Hou Biostatistician Date

Nanjing Aikeman Medical Information & Technology Co., Ltd.

Reviewed by:

DocuSigned by Juan Li
Juan Li
I have reviewed this document
April 26, 2024 | 4:59:13 PM CST
85344814E5A84935B82095F2BE94A5F3

4-26-2024

Juan Li Statistics Director Date

Nanjing Aikeman Medical Information & Technology Co., Ltd .

Signature Page

Reviewed by:

DocuSigned by Chen Sun
Chen Sun
I have reviewed this document
April 26, 2024 | 4:15:54 PM CST
3B017EOD612C43B8A469AF935230D89A

4-26-2024

Chen Sun Statistics Manager Date

Genenet Pharmaceutical Co., Ltd .

DocuSigned by Hongli Li
Hongli Li
I have reviewed this document
April 26, 2024 | 5:15:58 PM CST
AC9764A88B334443BA42AD240497B752

4-26-2024

Hongli Li Medical Manager Date

Genenet Pharmaceutical Co., Ltd .

Approved by:

DocuSigned by Rui Liu
Rui Liu
I approve this document
April 26, 2024 | 5:15:40 PM CST
F79639FC08534678887391696B866287

4-28-2024

Rui Liu Clinical Medical Director Date

Genenet Pharmaceutical Co., Ltd .

Table of Contents

TITLE PAGE 1
Signature Page 2
Signature Page 3
1. Introduction 8
2. Study Design 8
2.1 Study Objectives 8
2.1.1 Primary Objective 8
2.1.2 Secondary Objective 8
2.2 Overall Study Design 8
2.3 Randomization and Blinding 8
2.4 Hypothesis Test and Sample Size 8
2.4.1 Hypothesis Test 8
2.4.2 Sample Size 9
3. Evaluation Endpoints 9
3.1 Demographic and Other Baseline Indicators 9
3.1.1 Demographic Indicators 9
3.1.2 CMT1A Diagnosis 9
3.1.3 Other Baseline Indicators 9
3.2 Efficacy Endpoints 9
3.2.1 Primary Efficacy Endpoint 9
3.2.2 Secondary Efficacy Endpoints 9
3.2.3 Summary of Efficacy Endpoints 10
3.3 Safety Endpoints 11
3.3.1 Adverse Events 11
3.3.2 Laboratory Tests 11
3.3.3 Vital Signs 12
3.3.4 Physical Examination 12
3.3.5 12-lead ECG 12
4. Analysis Population 12
4.1 Definition of Analysis Population 12
4.2 Protocol Deviation 13
5. Statistical Analysis Methods 13
5.1 General Principles 13
5.1.1 Handling of Missing Data 13
5.1.2 Definition of Baseline 15
5.1.3 Analysis Time Window 15
5.2 Statistical Analysis Methods 16
5.2.1 Study Population 16
5.2.1.1 Subject Disposition 16
5.2.1.2 Major Protocol Deviations 17
5.2.2 Analysis of Demographics, CMT1A Diagnosis, and Other Baseline Characteristics 17
5.2.3 Prior/Concomitant Medications (not for CMT1A) 18
5.2.4 Prior/Concomitant Non-drug Treatment 19
5.2.5 Analysis of Investigational Drug Exposure and Compliance 19
5.2.6 Definition and Analysis of Estimand for Primary Efficacy Endpoint 19
5.2.7 Analysis of Secondary Efficacy Endpoints 21

5.2.7.1 Difference in ONLS Scores from Baseline at Months 6 and 12 21
5.2.7.2 Difference in ONLS Subscale Scores from Baseline at Month 15 22
5.2.7.3 ONLS Response Rate at the End of Treatment.. 22
5.2.7.4 Difference in CMTNS-v2 Total Scores and Subscale Scores from Baseline at Month 1522
5.2.7.5 Differences in 10MWT and 9HPT Scores from Baseline at Month 15 22
5.2.7.6 Difference in Mean Bilateral Ankle Dorsiflexion and Hand Grip Strength from Baseline at Month 15.. 23
5.2.7.7 Difference in Electrophysiological Parameters from Baseline at Month 15..................... 23
5.2.7.8 Differences of EQ-5D Utility Index and EQ-VAS Scores from Baseline at Month 15.... 23
5.2.8 Analysis of Safety Endpoints.. 24
6. Interim Analysis 25
7. Templates for Statistical Analysis Tables 27
8. Modifications to the Analysis Plan in the Protocol 27
9. References.. 27
10. SAS Code Reference.. 27

List of Abbreviations and Definition of Terms

Abbreviations	Definitions
μV	Microvolt
10MWT	10 meter Walking Test
9HPT	Nine Hole Peg Test
AE	Adverse Event
ANCOVA	Analysis of Covariance
ATC	Anatomical Therapeutic Chemical
BMI	Body Mass Index
cm	Centimeter
CMAP	Compound Muscle Action Potential
CMT1A	Charcot-Marie-Tooth Disease type 1A
CMTNS	Charcot-Marie-Tooth Neuropathy Score
CP	Conditional Power
CR	Copy Reference
CT	Computed Tomography
CTCAE	Common Terminology Criteria for Adverse Event
DML	Distant Motor Limit
EQ-5D	EuroQol-5 Dimension
FAS	Full Analysis Set
IDMC	Independent Data Monitoring Committee
IWRS	Interactive Web Response System
kg	kilogram
kg/m2	kilogram per Square Meter
m/s	Meter per Second
MAR	Missing at Random
MedDRA	Medical Dictionary for Regulatory Activities
ml	Milliliter
MMRM	Mixed Model Repeated Measure
ms	Millisecond
mV	Millivolt
N	Newton
NCV	Nerve Conduction Velocity
ONLS	Overall Neuropathy Limitation Scale
PASS	Power Analysis and Sample Size
PMP22	Peripheral Myelin Protein 22
PN	Preferred Name
PPS	Per Protocol Set
PT	Preferred Term
PXT3003	Fixed dose combination of baclofen, naltrexone and sorbitol
QMT	Quantified Muscular Testing
SAE	Serious Adverse Event
SAP	Statistical Analysis Plan
SAS	Statistical Analysis System
SNAP	Sensory Nerve Action Potential
SOC	System Organ Class
SS	Safety Set
TEAE	Treatment Emergent Adverse Event

Abbreviations	Definitions
TESAE	Treatment Emergent Serious Adverse Event
VAS	Visual Analogue Scale
WHODrug	World Health Organization Drug Dictionary

1. Introduction

This statistical analysis plan is developed based on the protocol for "A Multicenter, Randomized, Double-blind, Placebo-controlled Phase III Clinical Trial to Assess the Efficacy and Safety of PXT3003 in Patients with Charcot-Marie-Tooth Disease Type 1A for 15 Months" (Protocol No.: TSL-CM-PXT3003-III, Version No.: V1.0, Version Date: March 18, 2021). The statistical analysis methods, data processing principles, and result presentation methods will be provided.

2. Study Design

2.1 Study Objectives

2.1.1 Primary Objective

To assess the efficacy of PXT3003 on Charcot-Marie-Tooth Disease type 1A measured by the Overall Neuropathy Limitation Scale (ONLS) scores in patients treated for 15 months.

2.1.2 Secondary Objective

To assess the efficacy of PXT3003 on functional tests, electrophysiological parameters, and quality of life measures, and to assess the safety of PXT3003.

2.2 Overall Study Design

This is a multicenter, randomized, double-blind, placebo-controlled Phase III study to evaluate the efficacy and safety of PXT3003 in patients with Charcot-Marie-Tooth disease type 1A for 15 months.

2.3 Randomization and Blinding

After screening, the investigator will randomly assign the eligible subjects to the investigational drug group or the placebo group through an interactive web response system **(IWRS).** The subject randomization list of **IWRS** was generated in a 1:1 ratio using the block randomization method through the SAS@9.4 version of **PROC PLAN.**

This is a double-blind study. The investigational products (including placebo and investigational drug) will be classified, numbered, labeled, and blinded by personnel not involved in this study according to the generated drug randomization list. The drug randomization list of IWRS was generated in a 1:1 ratio using the block randomization method through the SAS@9.4 version of PROC PLAN. The IWRS will be used to maintain the integrity of blinding.

2.4 Hypothesis Test and Sample Size

2.4.1 Hypothesis Test

The two-sidedtest will be adopted for this study. The null hypothesis for this test is H_0: $\delta=0$, indicating that the investigational drug group shows the same improvement in ONLS scores as the placebo group. The alternative hypothesis is H_1: $\delta\neq0$, suggesting that the investigational drug group differs from the placebo group in the improvement of ONLS scores. In the formula, δ = the difference in ONLS scores from baseline at Month 15 in the investigational drug group - the difference in ONLS scores from baseline at Month 15 in the placebo group. (Note: The lower the ONLS score, the better)

2.4.2 Sample Size

The randomization ratio for this study is 1:1. Assuming that the difference in the improvement in ONLS scores from baseline between the investigational drug group and the placebo group is 0.3 and the standard deviation is 0.6, type I error is controlled at 0.05 (two-sided), and at least 80% power is given to test the superiority of the investigational drug group over the placebo group, 70 evaluable subjects are required for each group. Based on a 20% dropout rate, 88 subjects are required to be enrolled in each group, and thus, the total sample size is 176.

The sample size is calculated on PASS (version 15.0).

3. Evaluation Endpoints

3.1 Demographic and Other Baseline Variables

3.1.1 Demographic Variables

Demographic variables include age (years), gender, baseline height (cm), baseline weight (kg), baseline BMI (kg/m^2), and marital status. The relevant calculation method is as follows:
Baseline BMI (kg/m^2) = baseline weight (kg) / (baseline height (cm)/100)^2, with the result rounded to one decimal place.

3.1.2 CMT1A Diagnosis

Variables related to the diagnosis of CMT1A include whether CMT1A is confirmed, clinical manifestations, family history of genetic disorders, presence of chronic peripheral neuropathy, motor conduction velocity of the ulnar nerve > 15 m/s, and duplication of the PMP22 gene.

3.1.3 Other Baseline Variables

Other baseline variables include personal history (alcohol use, smoking, and allergies), infectious disease history, past/present medical history, history of previous surgeries/procedures, prior/concomitant medications, prior/concomitant non-drug treatment, history of drug treatment for CMT1A, history of non-drug treatment for CMT1A, chest PA X-ray or chest CT scan, abdominal ultrasound, and virological screening.

3.2 Efficacy Endpoints

3.2.1 Primary Efficacy Endpoint

The degree of improvement in the ONLS score. It is defined as the difference in the ONLS score from baseline at Month 15.

3.2.2 Secondary Efficacy Endpoints

(1) Treatment response rate for PXT3003;

- Treatment response rate 1: The proportion of subjects with improvement (at least a 1-point decrease from baseline) in ONLS score at the end of treatment;
- Treatment response rate 2: The proportion of subjects with no increase in ONLS score at the end of treatment;

(2) Arm and leg subscale scores in ONLS;

(3) Improvement in Charcot-Marie-Tooth Neuropathy Score second version (CMTNS-V2) total scores and subscale scores;

(4) Time of 10-meter walking test (10 MWT);

(5) Nine hole peg test (9HPT) with the non-dominant hand;

(6) Quantified muscular testing (QMT) for grip strength and foot dorsiflexion;

(7) Electrophysiological parameters of sensory and motor nerve conduction of the ulnar and radial nerves (non-dominant side), including:

- Amplitude of ulnar nerve compound muscle action potential
- Amplitude of radial nerve sensory nerve action potential
- Ulnar nerve conduction velocity
- Radial nerve conduction velocity
- Ulnar nerve distant motor limit
- Radial nerve distant motor limit

(8) Quality of life evaluation

- EuroQol-5 dimension (EQ-5D) evaluation
- EQ-VAS score

3.2.3 Summary of Efficacy Endpoints

Endpoints	Description	Improvement Direction
**Primary Efficacy Endpoint**		
ONLS	Overall Neuropathy Limitation Scale	Decrease
**Secondary Efficacy Endpoints**		
ONLS response rate 1	Responders are defined as patients with at least a 1-point decrease in the ONLS score from baseline at the end of treatment	
ONLS response rate 2	Responders are defined as patients with no increase in the ONLS score from baseline at the end of treatment	
ONLS subscale scores	Arm and leg subscale scores in ONLS	Decrease
CMTNS-v2 total score	Charcot-Marie-Tooth Neuropathy Score second version	Decrease
CMTNS-V2 subscale scores	9 subscales of CMTNS-v2	Decrease
10MWT (seconds)	Time to walk 10 meters	Decrease
9HPT (seconds)	Time to complete 9HPT with the non-dominant hand	Decrease
Hand grip strength (kg)	Hand grip strength test performed on both sides	Increase
Ankle dorsiflexion (N)	Ankle dorsiflexion test performed on both sides	Increase
CMAP amplitude of ulnar nerve (mV)	Amplitude of ulnar nerve (non-dominant side) compound muscle action potential	Increase
SNAP amplitude of radial nerve (μV)	Amplitude of radial nerve (non-dominant side) sensory nerve action potential	Increase
NCV of ulnar and radial nerves (m/s)	Nerve conduction velocity of ulnar and radial nerves (non-dominant side)	Increase
DML of ulnar and radial nerves (ms)	Distant motor limit of ulnar and radial nerves (non-dominant side)	Decrease
EQ-5D utility index	Utility index value of European Quality of Life 5 Dimensions	Increase

Endpoints	Description	Improvement Direction
EQ-VAS score	Subjects' subjective rating of their health status	Increase

3.3 Safety Endpoints

Safety endpoints include various adverse events (AEs), vital signs, physical examinations, laboratory tests, and 12-lead ECG. The specific descriptions are as follows:

3.3.1 Adverse Events

Adverse Event (AE): An untoward medical event that occurs after subjects receive the investigational products until the end of the trial, regardless of causality with the investigational drug. Symptoms/signs that the subjects show during the screening period of the trial will be recorded as medical history. Any worsening of the symptoms/signs during the trial will be recorded as an AE.

Treatment-related adverse event (TRAE): An AE with a relationship of "definitely related", "probably related", and "possibly related" to the investigational drug.

Treatment emergent adverse event (TEAE): An AE that (i) occurs on the day of the first dose of the investigational drug or afterwards, or (ii) occurs before the first dose of the investigational drug but with an escalation in CTCAE grade postdose.

Serious adverse event (SAE): Any death, life-threatening event, event requiring hospitalization or prolonged hospitalization, leading to permanent or serious disability, loss of function, congenital anomalies or birth defects, and other important medical event after the subject receives the investigational products. If the above situations may occur without treatment, a decision based on medical judgment is required.

Significant adverse event: According to the *Guidelines for the Planning and Reporting of Data Management and Statistical Analysis of Drug Clinical Trials* (National Medical Products Administration (NMPA) Announcement No. 113, 2016), an significant adverse event refers to any adverse event that leads to targeted medical measures (such as drug discontinuation, dose reduction and symptomatic treatment) and obvious abnormalities detected in hematology or other laboratory tests except serious adverse events.

For this study, according to the above definition, a significant adverse event is defined as any adverse event that results in treatment measures being taken for the investigational drug (including dose interruption, dose reduction, dose interruption combined with dose reduction, and permanent discontinuation) in addition to serious adverse events. This also includes adverse events that require concomitant medication or non-drug treatment, or those classified under the system organ class (SOC) of "investigations" after coding using the MedDRA Version 24.0 or higher versions.

3.3.2 Laboratory Tests

The examination items of hematology, blood chemistry, urinalysis, and pregnancy test are shown in the table below.

Examination Items	Indicators
Hematology	White blood cell count
	Red blood cell count
	Hemoglobin
	Platelet count

Examination Items	Indicators
	Neutrophil count
	Lymphocyte count
Urinalysis	Protein urine
	Glucose urine
	White blood cells urine
	Red blood cells urine
Blood chemistry	Alanine aminotransferase
	Aspartate aminotransferase
	Alkaline phosphatase
	Gamma-glutamyl transpeptidase
	Total protein
	Albumin
	Total bilirubin
	Direct bilirubin
	Creatinine
	Urea/Urea nitrogen
	Glucose
	Potassium
	Sodium
	Chloride
	Glycosylated hemoglobin
Serum pregnancy test for females	Blood β-human chorionic gonadotropin

3.3.3 Vital Signs

Vital signs in this study include pulse, sitting systolic and diastolic blood pressure, and body temperature (axillary).

3.3.4 Physical Examination

Physical examinations in this study include skin mucosa, lymph nodes, head and neck (eyes, oropharynx, thyroid), chest (heart, respiratory system), abdomen, musculoskeletal system, and nervous system.

3.3.5 12-lead ECG

Clinical judgment and classification of normal/abnormal 12-lead ECG examination results.

4. Analysis Sets

4.1 Definition of Analysis Sets

The analysis sets will be jointly determined and recorded by the sponsor, principal investigator, and statistician during the data review meeting before the database lock.

Full analysis set (FAS): All randomized subjects who provide baseline and at least one post-baseline ONLS score. The assessment of primary and secondary efficacy will be based on FAS and analyzed according to the grouping at randomization.

Per protocol set (PPS): All subjects in FAS with no major protocol deviations impacting efficacy (defined during the blinded data review meeting) from baseline to the Month 15 visit. The **PPS** will be used for the assessment of primary and secondary efficacy to support FAS-based results.

The protocol deviation under the category of medication compliance classified as a major protocol deviation affecting efficacy, as defined above, refers to cases where the theoretical dose compliance calculated according to Section 5.5.2 is less than 80% or greater than 120%, or falls outside the compliance range standard determined by the data review meeting.

Safety set (SS): All randomized subjects who receive at least 1 dose of the investigational drug. Subjects will be analyzed according to the treatment actually received. All safety analyses will be performed based on the SS.

4.2 Protocol Deviation

Subjects may be excluded from the PPS if they have a major protocol deviation. See "6. Category of Protocol Deviations" in the *Protocol Deviation Plan* for the categories of protocol deviations. The process of identifying, managing, reviewing, and recording protocol deviations is shown in the *Protocol Deviation Plan*. Before the database lock, the subject population division will be discussed at the data review meeting and ultimately approved by the sponsor.

All major protocol deviations defined in the *Protocol Deviation Definition List* (by category of protocol deviations) will be summarized and tabulated.

5. Statistical Analysis Methods

The statistical analysis software will be SAS@9.4 or a later release.

All efficacy and safety endpoints will be summarized descriptively at each visit by treatment groups. Continuous variables will be described using the number of subjects, arithmetic mean, standard deviation, median, quartile, minimum, and maximum. For the maximum and minimum, the same number of decimal places as the raw data (or analysis variables) will be retained; for the arithmetic mean and median, one additional decimal place than the raw data (or analysis variables) will be retained; for the standard deviation, two additional decimal places than the raw data (or analysis variables) will be retained; for descriptive statistics, up to four decimal places will be retained. Categorical variables will be described using the number and percentage of cases. Percentages will be rounded to 1 decimal place. Unless otherwise specified, the denominator of the percentage will be the total number of subjects in the corresponding treatment group of the analysis sets.

For two-sided tests, if the *P* value is less than or equal to 0.05, the difference tested will be considered statistically significant (unless otherwise specified). The P value will be rounded to 4 decimal places and displayed as "<0.0001" if it is <0.0001 and ">0.9999" if it is >0.9999.

The efficacy endpoints will be analyzed based on the FAS and PPS, with the primary results obtained based on the FAS. The analysis of safety endpoints will be conducted based on the SS.

If the reasons for not being examined are not collected, only the results of subjects who have undergone relevant examinations will be presented in the data list.

5.1 General Principles

5.1.1 Handling of Missing Data

- Laboratory values

If laboratory values are recorded as being below (or equal to) or above (or equal to) the test range value (e.g., <x, ≤x, >x, ≥x), they will be processed as the test range value (i.e., =x) when summarizing descriptive statistics. However, they will still be presented as per the CRF records in the data list, i.e., "<x", "≤x", ">x", or "≥x".

- Dates of AE, history of drug treatment for CMT1A, prior/concomitant medications, or prior/concomitant non-drug treatment

For missing dates of AE, history of drug treatment for CMT1A, prior/concomitant medications, or prior/concomitant non-drug treatment, if a comparison with the first dose date is required, unless otherwise specified, fill in the missing date according to the following method before making a judgment:

- The missing dates of AEs will be imputed as follows:
- ○ Missing AE start date
  1) If the year and month are known and the year and month are not equal to the year and month of the first dose, the date will be imputed with the first day of the known month.
  2) If the year and month are known and are equal to those of the first dose date, make the start date of AE equal to the first dose date.
  3) If only the year is known and the year is not equal to that of the first dose date, the date will be imputed with "January 01".
  4) If only the year is known and is equal to that of the first dose date, make the start date of AE equal to the first dose date.
  5) If the year, month, and day are missing, take the date of the first dose as the start date of AE.
- ○ Missing date of AE with the highest CTCAE grade
  1) If the year and month are known and the year and month are not equal to the year and month of the first dose, the date will be imputed with the last day of the known month.
  2) If the year and month are known and are equal to those of the first dose date, make the highest CTCAE grading date equal to the first dose date.
  3) If only the year is known and the year is not equal to that of the first dose date, the date will be imputed with "December 31".
  4) If only the year is known and is equal to that of the first dose date, make the highest CTCAE grading date equal to the first dose date.
  5) If the year, month, and day are missing, take the date of the first dose as the highest CTCAE grading date.
  6) If the imputed highest CTCAE grading date is after the end date, the day before the end date should be taken as the highest CTCAE grading date.
- ○ Missing AE end date
  1) If the year and month are known, the date will be imputed with the last day of the known month.
  2) If only the year is known, the date will be imputed with "December 31".

3) If the imputed start date is after the end date, the end date should be used as the corresponding start date.

- Missing dates for history of drug treatment for CMT1A, prior/concomitant medications, or prior/concomitant non-drug treatment should be imputed as follows:

○ Missing start dates for history of drug treatment for CMT1A, prior/concomitant medications, or prior/concomitant non-drug treatment

1) If the year and month are known and the day is missing, the date will be imputed with the first day (-01) of the known month.

2) If only the year is known and the day and month are missing, the date will be imputed with the first day of January (January 01) of the known year.

3) If the year, month, and day are completely missing, no imputation will be conducted. Only the end date will be used to determine whether it is before or after the first dose date.

○ Missing end dates for history of drug treatment for CMT1A, prior/concomitant medications, or prior/concomitant non-drug treatment

1) If the year and month are known, the date will be imputed with the last day of the known month.

2) If only the year is known, the date will be imputed with "December 31".

3) If the year, month, and day are completely missing and the "Ongoing or Not" column is not filled with "Yes", no imputation will be conducted, and it will be assumed by default that there is concomitant medication/concomitant non-drug treatment.

Missing values of other dates will not be processed.

Note: The data will still be listed according to the collected raw data.

- Missing primary efficacy endpoint

The primary efficacy endpoint may be missing in the FAS. If a subject's primary efficacy endpoint is missing, the missing values will be conservatively imputed using the predicted value (Copy Reference, CR) derived from the linear model established based on the placebo group data, and extrapolated from the results of multiple imputations (number of multiple imputations = 100).

5.1.2 Definition of Baseline

Unless otherwise specified, the baseline of an endpoint in this trial is defined as the last non-missing measurement result obtained for that endpoint before the first dose of the investigational product. If there is information indicating that the examination on the day of the first dose of the investigational product is performed before the first dose, the examination data will be used as baseline data.

5.1.3 Analysis Time Window

End of treatment visit data or unscheduled visit data (if any) for laboratory tests, vital signs, physical examination, and 12-lead ECG will be classified into the corresponding analysis visits according to the following analysis time windows.

Table 1 Analysis Time Window for Laboratory Tests, Vital Signs, Physical Examination, and 12-Lead ECG

Analysis Visit	Target Study Days [1]	Analysis Time Window (Days)

Baseline	1	[-30,1]
Visit 1 (Month 3)	90	[2,134]
Visit 2 (Month 6)	180	[135,224]
Visit 3 (Month 9)	270	[225,314]
Visit 4 (Month 12)	360	[315,404]
Visit 5 (Month 15)	450	[405,460]
Note: [1] Study days is defined as the length of time (days) from the first dose to a target date. If the target date is before the date of first dose, then study days = target date - date of first dose; if the target date is on the same day as the date of first dose or after the date of first dose, then study days = target date - date of first dose + 1.		

If multiple data entries fall within the corresponding analysis time window, the priority order for the data selected for analysis is as follows: (1) Data from scheduled visits; (2) Data from end of treatment visits; (3) Data from unscheduled visits that are closest to the target number of study days (if two unscheduled visits are equidistant from the target number of study days, the data from the unscheduled visit that occurs later than the target number of study days will be selected).

Data from end of treatment visits or unscheduled visits (if any) for ONLS, CMTNS-v2, EQ-5D, electrophysiological test parameters, 10MWT, 9HPT, and QMT (hand grip strength and ankle dorsiflexion) will be classified into the corresponding analysis visits according to the following analysis time windows.

Table 2 Analysis Time Windows for ONLS, CMTNS-v2, EQ-5D, Electrophysiological Test Parameters, 10MWT, 9HPT, and QMT

Analysis Visit	Target Study Days [1]	Analysis Time Window (Days)
Baseline	1	[-30,1]
Visit 2 (Month 6)	180	[2,269]
Visit 4 (Month 12)	360	[270,404]
Visit 5 (Month 15)	450	[405,495]
Note: [1] Study days is defined as the length of time (days) from the first dose to a target date. If the target date is before the date of first dose, then study days = target date - date of first dose; if the target date is on the same day as the date of first dose or after the date of first dose, then study days = target date - date of first dose + 1.		

If multiple data entries fall within the corresponding analysis time window, the priority order for the data selected for analysis is as follows: (1) Data from scheduled visits; (2) Data from end of treatment visits; (3) Data from unscheduled visits that are closest to the target number of study days (if two unscheduled visits are equidistant from the target number of study days, the data from the unscheduled visit that occurs later than the target number of study days will be selected).

5.2 Statistical Analysis Methods

Unless otherwise specified, the baseline data will be summarized and analyzed descriptively by randomization and pooled groups. Efficacy endpoints will be summarized and analyzed descriptively by randomization group (and analysis visit). Safety endpoints will be summarized and analyzed descriptively according to the actual treatment group and the pooled group (and analysis visit).

5.2.1 Study Population

5.2.1.1 Subject Disposition

The number of all screened subjects, screening failures, and the number and percentage of subjects with varying reasons for screening failure will be summarized. The number of enrolled subjects by randomization will be summarized by randomization and pooled groups.

The number and percentage of subjects who complete the study, withdraw early from the study, and the primary reasons for early withdrawal will be summarized by randomization and pooled groups.

The number and percentage of subjects included in each analysis set (FAS, PPS, and SS) will be summarized by randomization and pooled groups.

The reasons for subject screening failure will be listed.

The list will describe the study completion status of each enrolled subject.

The inclusion details of enrolled subjects in each analysis data set and the reasons for being excluded from the efficacy analysis set will be listed.

5.2.1.2 Major Protocol Deviations

The number and percentage of subjects with at least one major protocol deviation under each protocol deviation category will be summarized by randomization and pooled groups.

All protocol deviations for the enrolled subjects will be listed.

5.2.2 Analysis of Demographics, CMT1A Diagnosis, and Other Baseline Characteristics

(1) Demographics

Demographic data of subjects, including age (years), gender, baseline height (cm), baseline weight (kg), baseline BMI (kg/m²), and marital status, will be summarized by randomization and pooled groups.

Demographic data of subjects in the FAS will be listed.

(2) CMT1A Diagnosis

The CMT1A diagnosis of the subjects, including whether CMT1A is confirmed, clinical manifestations, family history of genetic disorders, presence of chronic peripheral neuropathy, motor conduction velocity of the ulnar nerve > 15 m/s, and duplication of the PMP22 gene, will be summarized by randomization and pooled groups.

The CMT1A diagnosis results and PMP22 gene test results of subjects in the FAS will be listed.

(3) Prior and Concomitant Medication for CMT1A

Prior/concomitant medication for CMT1A will be coded using the Chinese version of WHODrug Global B3 format (September 01, 2021) or higher. The data here are derived from the records on the CRF [History of Drug Treatment for CMT1A] and the records for CMT1A on the form [Prior/Concomitant Medications] (the field "Prophylactic Medication or Other Instructions" is filled with "Medication for this Study Disease").

Prior medication for CMT1A is defined as the administration of non-investigational drugs for CMT1A with an end date earlier than the first dose of the investigational drug.

Concomitant medication for CMT1A is defined as the administration of non-investigational drugs for CMT1A with a start date earlier than the first dose of the investigational drug but an end date later than or equal to the first dose of the investigational drug, that is ongoing, or with a start date later than or equal to the first dose of the investigational drug.

The number and percentage of subjects with prior and concomitant medications for CMT1A will be summarized by randomization and pooled groups, treatment subgroup ATC (Anatomical Therapeutic Chemical) level 2, and preferred name (PN).

The drug treatment for CMT1A of subjects in the FAS will be listed.

(4) History of Prior Non-drug Treatment for CMT1A

The history of prior non-drug treatment for CMT1A will be coded using the Chinese version of MedDRA 24.1 or higher.

The number and percentage of subjects with a history of prior non-drug treatment for CMT1A will be summarized by randomization and pooled groups, SOC, and preferred term (PT).

The history of prior non-drug treatment for CMT1A of subjects in the FAS will be listed.

(5) Past/Present Medical History

The past/present medical history will be coded using the Chinese version of MedDRA 24.1 or higher.

The number and percentage of subjects with past/present medical history will be summarized by randomization and pooled groups, SOC, and PT.

The past/present medical history of subjects in the FAS will be listed.

(6) History of Previous Surgeries/Procedures

The history of previous surgeries/procedures will be coded using the Chinese version of MedDRA 24.1 or higher.

The number and percentage of subjects with a history of previous surgeries/procedures will be summarized by randomization and pooled groups, SOC, and PT.

The history of previous surgeries/procedures of subjects in the FAS will be listed.

(7) Infectious Disease History

The infectious disease history will be coded using the Chinese version of MedDRA 24.1 or higher.

The number and percentage of subjects with infectious disease history will be summarized by randomization and pooled groups, SOC, and PT.

The infectious disease history of subjects in the FAS will be listed.

(8) Personal History

The number and percentage of subjects with status of alcohol use, smoking status, drug allergy history, food allergy history, and other allergy histories will be summarized by randomization and pooled groups.

The histories of alcohol use, smoking, and allergies of subjects in the FAS will be listed.

(9) Chest PA X-ray or Chest CT Scan, Abdominal Ultrasound, and Virological Screening During the Screening Period

The number and percentage of subjects for clinical assessment and judgement of the results of chest PA X-ray, chest CT scan, abdominal ultrasound examination, and virological screening will be summarized by randomization and pooled groups.

The chest PA X-ray or chest CT scan, abdominal ultrasound, and virological screening of subjects in the FAS during the screening period will be listed.

5.2.3 Prior/Concomitant Medications (not for CMT1A)

The prior/concomitant medications will be coded using the Chinese version of WHODrug Global B3 format (September 01, 2021) or higher.

Prior medications are defined as the administration of non-investigational drugs with an end date earlier than the first dose of the investigational drug.

Concomitant medication is defined as the administration of non-investigational drugs with a start date earlier than the first dose of the investigational drug but an end date later than or equal to the first dose of the investigational drug, that is ongoing, or with a start date later than or equal to the first dose of the investigational drug.

The number and percentage of subjects with prior and concomitant medications will be summarized by randomization and pooled groups, treatment subgroup ATC level 2, and PN.

Prior and concomitant medications of subjects in the FAS will be listed.

5.2.4 Prior/Concomitant Non-drug Treatment

The prior/concomitant non-drug treatment will be coded using the Chinese version of MedDRA 24.1 or higher.

Prior non-drug treatment is defined as a non-drug treatment with an end date earlier than the first dose date of the investigational drug.

Concomitant non-drug treatment is defined as a non-drug treatment with a start date earlier than the first dose of the investigational drug but an end date later than or equal to the first dose of the investigational drug, that is ongoing, or with a start date later than or equal to the first dose of the investigational drug.

The number and percentage of subjects with prior and concomitant non-drug treatment will be summarized by randomization and pooled groups, SOC, and PT.

Prior and concomitant non-drug treatment for subjects in the FAS will be listed.

5.2.5 Analysis of Investigational Drug Exposure and Compliance

Duration of drug treatment (days) = (Date of the last dose - Date of the first dose + 1).

Actual dose (mL) = ∑ (Actual dose (mL/day) * (End date - start date + 1) + Cumulative overdose (mL) - Cumulative missed dose (mL).

Actual dose intensity (mL/day) = Actual dose (mL)/Duration of drug treatment (days).

If the duration of drug treatment is less than or equal to 14 days, the expected dose (mL) = Duration of drug treatment * 10 mL; if the duration of drug treatment is greater than 14 days, the expected dose (mL) = Duration of drug treatment * 20 mL - 140 mL.

Theoretical dose compliance (%) = Actual dose (mL)/Expected dose (mL) * 100%.

Ordered dose compliance (%) = Actual dose (mL)/Total ordered dose (mL) * 100%. Among them, the total ordered dose (mL) = ∑ (ordered dose(mL/day) * (end date - start date + 1)).

The subjects' investigational products exposure and dose compliance will be summarized by actual treatment group, including the duration of drug treatment, actual dose, actual dose intensity, theoretical dose compliance, and ordered dose compliance. The number and percentage of subjects will be summarized by the actual treatment group regarding whether at least one dose adjustment is performed, the reasons for each dose adjustment, and the classifications of theoretical dose compliance (<80%, 80%–120%, and >120%) and ordered dose compliance (<80%, 80%–120%, and >120%).

The administration, drug distribution and dispensing, exposure to the investigational products, and medication compliance of subjects in the SS will be listed.

5.2.6 Definition and Analysis of Estimand for Primary Efficacy Endpoint

The clinical question corresponding to the estimand for the primary efficacy endpoint is whether the change in ONLS from baseline at Month 15 in the investigational drug group is superior to that in the placebo group among all CMT1A patients who are randomized with baseline and at least one post-baseline ONLS score. The estimand is defined as:

Population	All CMT1A patients who are randomized with baseline and at least one post-baseline ONLS score
Endpoint	Difference in the ONLS score from baseline at Month 15
Treatment	Treatment with PXT3003 oral solution or placebo for 15 months. See Section 6.1 of the protocol for specific dosage and administration
Strategies for Addressing Intercurrent Events	○ Premature termination of treatment for reasons other than poor efficacy as judged by the investigator (hypothetical strategy: assuming that the efficacy of subjects who prematurely terminate treatment for reasons other than poor efficacy as judged by the investigator is similar to the observed subject data, the ONLS score at Month 15 will be imputed using a regression method)<br>○ Premature termination of treatment due to poor efficacy as judged by the investigator (composite strategy: ONLS value at Month 15 for subjects with this intercurrent event will be carried forward using the LOCF method)<br>○ Use of prohibited treatments that affect efficacy assessment [1] (hypothetical strategy: assuming that the efficacy of subjects after using prohibited treatments that affect efficacy assessment is similar to the observed subject data, the ONLS score at Month 15 will be imputed using a regression method)<br>○ Continuous discontinuation lasting more than 1 month or intermittent discontinuation for more than 3 months (Treatment policy strategy)
Population-level summary	Mean difference in the ONLS score change from baseline at Month 15 between the two groups
Note: [1] Drugs and non-drug treatments that may affect efficacy assessment are included.	

Two conditions of missing data should also be considered in the data analysis: 1) Intermediate missing data caused by missed follow-ups or data collection will be imputed into a monotone missing pattern using the MCMC (NIMPUTE=100) method in PROC MI; 2) Missing data at Month 15 resulting from missed follow-ups or data collection will be imputed using the CR method.

Primary analysis of estimands:

The primary analysis will be based on the FAS, which includes all randomized subjects with baseline and at least one post-baseline ONLS score. Multiple complete data sets based on multiple imputation will be analyzed using an analysis of covariance (ANCOVA) model, with group and baseline as covariates. The estimated results for each complete data set will be combined following Rubin's rule to provide the least squares mean (LSM) for each group, the LSM of the treatment difference, the two-sided *P* value, and the 95% confidence interval (CI).

Sensitivity analysis of estimands: For the above primary analyses, the following sensitivity analyses will be performed to assess the robustness of the primary estimator.

**Analysis Type**	**Analysis Population**	**"Missing" mode assumption**	**Statistical Analysis Methods**
Sensitivity analysis 1	FAS	Imputation based on the regression method under the MAR hypothesis and monotone missing pattern	Multiple imputation + ANCOVA

Complementary analysis of estimands:

Descriptive statistics will be used to summarize the ONLS scores and subscale scores at baseline and each post-baseline analysis visit, and the differences in the ONLS scores and subscale scores at each post-baseline analysis visit from baseline by groups. A line chart will be plotted to display the ONLS scores and subscale scores at baseline and each post-baseline analysis visit, as well as the differences from baseline by groups.

For the above primary analyses, the following supplementary analyses will be performed to assess the robustness of the primary estimator.

<table>
<tr><th>Intercurrent Events</th><th>Supplementary Analysis Estimand 1</th><th>Supplementary Analysis Estimand 2</th><th>Supplementary Analysis Estimand 3</th></tr>
<tr><td>Premature termination of treatment for reasons other than poor efficacy as judged by the investigator</td><td rowspan="3">Hypothetical strategy: Assuming that the efficacy of subjects after intercurrent events is similar to the data observed in the control group, the ONLS score at Month 15 will be imputed using a CR-based method.</td><td rowspan="4">Analysis will be conducted on the PPS, using the same method as the primary analysis.</td><td rowspan="4">Without considering the impact of intercurrent events, the missing data will be imputed using a mixed model for repeated measures (MMRM) based on the MAR hypothesis.</td></tr>
<tr><td>Use of prohibited treatments that affect efficacy assessment</td></tr>
<tr><td>Continuous discontinuation lasting more than 1 month or intermittent discontinuation for more than 3 months</td></tr>
<tr><td>Premature termination of treatment due to poor efficacy as judged by the investigator</td><td>Hypothetical strategy: The ONLS score for subjects experiencing this intercurrent event at Month 15 will be carried forward using the LOCF method.</td></tr>
</table>

Supplementary Analysis Estimand 1: The primary efficacy endpoint is defined as the difference in the ONLS score from baseline at Month 15. The primary efficacy endpoint will be analyzed using the ANCOVA model in the FAS, with baseline value as a covariate and group as a fixed effect. "Poor efficacy as judged by the investigator" will be imputed using the last non-missing data post-baseline, while other data will be imputed using the CR method. The LSM and 95% CI of the difference in the ONLS score from baseline at Month 15 between the PXT3003 group and the placebo group will be presented, along with the LSM difference, 95% CI, and corresponding P value for the difference in ONLS score from baseline at Month 15 between the two groups.

Supplementary Analysis Estimand 2: Analysis will be conducted on the PPS, using the same method as the primary analysis.

Supplementary Analysis Estimand 3: The MMRM will be used to analyze the difference in ONLS score from baseline at each post-baseline analysis visit using the raw data without imputation of missing values in the FAS. In this model, group, visit, and the interaction between group and visit will be treated as fixed effects, while subjects will be treated as a random effect, and the baseline value will be included as a covariate. The LSM and 95% CI of the difference in the ONLS score from baseline at Month 15 between the PXT3003 group and the placebo group will be presented, along with the LSM difference, 95% CI, and corresponding P value for the difference in ONLS score from baseline at Month 15 between the two groups.

The ONLS scores of subjects in the FAS will be listed.

5.2.7 Analysis of Secondary Efficacy Endpoints

5.2.7.1 Difference in ONLS Scores from Baseline at Months 6 and 12

The difference in ONLS scores from baseline at Months 6 and 12 will be analyzed using the ANCOVA model, with baseline value as a covariate and group as a fixed effect. Missing values will be imputed using the CR method.

5.2.7.2 Difference in ONLS Subscale Scores from Baseline at Month 15

The difference in ONLS subscale (arm and leg) scores from baseline at Month 15 will be analyzed using the ANCOVA model, with baseline value as a covariate and group as a fixed effect. Missing values will be imputed using the CR method.

5.2.7.3 ONLS Response Rate at the End of Treatment

There are 2 types of ONLS response rates. ONLS response rate 1 is defined as the proportion of subjects with improvement (at least a 1- point decrease from baseline) in ONLS score at the end of treatment, while ONLS response rate 2 is defined as the proportion of subjects with no increase in ONLS score at the end of treatment.

Descriptive statistics of the ONLS response rate 1 will be provided by group, with its 95% CI calculated using the exact (Clopper-Pearson) method, and Fisher's exact test will be used for inter-group comparisons. The difference in ONLS response rate 1 between the PXT3003 group and the placebo group will be calculated, and the Newcombe-Wilson method will be used to calculate the 95% CI of the rate difference.

The ONLS response rate 2 will be analyzed using the same method as above.

5.2.7.4 Difference in CMTNS-v2 Total Scores and Subscale Scores from Baseline at Month 15

Descriptive statistics will be used to summarize the CMTNS-v2 total scores and subscale scores at baseline and each post-baseline analysis visit, and the differences in the CMTNS-v2 total scores and subscale scores from baseline at each post-baseline analysis visit by randomization groups.

A line chart will be plotted to display the CMTNS-v2 total scores and subscale scores at baseline and each post-baseline analysis visit, as well as the differences from baseline by randomization groups.

The difference in CMTNS-v2 total scores and subscale scores from baseline at Month 15 will be analyzed using the ANCOVA model, with baseline value as a covariate and group as a fixed effect. Missing values will be imputed using the CR method. CMTNS-v2 sensory score = sensory symptom score + pinprick sensibility score + vibration score.

CMTNS-v2 examination score = sensory symptom score + motor symptom (legs) score + motor symptom (arms) score + pinprick sensibility score + vibration score + strength (legs) score + strength (arms) score.

The above analysis is also applicable to the CMTNS-v2 sensory score and the CMTNS-v2 examination score.

The CMTNS-v2 scores of subjects in the FAS will be listed.

5.2.7.5 Differences in 10MWT and 9HPT Scores from Baseline at Month 15

Descriptive statistics will be used to summarize the 10MWT and 9HPT scores at baseline and each post-baseline analysis visit, and the differences in 10MWT and 9HPT scores from baseline at each post-baseline analysis visit by randomization groups.

A line chart will be plotted to display the 10MWT and 9HPT scores at baseline and each post-baseline analysis visit, as well as the differences from baseline by randomization groups.

The differences in 10MWT and 9HPT scores from baseline at Month 15 will be analyzed using the ANCOVA model, with baseline value as a covariate and group as a fixed effect. Missing values will

be imputed using the CR method. The 10MWT and 9HPT results of subjects in the FAS will be listed.

5.2.7.6 Difference in Mean Bilateral Ankle Dorsiflexion and Hand Grip Strength from Baseline at Month 15

Descriptive statistics will be used to summarize the mean bilateral ankle dorsiflexion and hand grip strength at baseline and each post-baseline analysis visit, and the differences in mean bilateral ankle dorsiflexion and hand grip strength from baseline at each post-baseline analysis visit by randomization groups.

A line chart will be plotted to display the mean bilateral ankle dorsiflexion and hand grip strength at baseline and each post-baseline analysis visit, as well as the differences from baseline by randomization groups.

The difference in mean bilateral ankle dorsiflexion and hand grip strength from baseline at Month 15 will be analyzed using the ANCOVA model, with baseline value as a covariate and group as a fixed effect. Missing values will be imputed using the CR method. The test results of ankle dorsiflexion and hand grip strength for subjects in the FAS will be listed.

5.2.7.7 Difference in Electrophysiological Parameters from Baseline at Month 15

Descriptive statistics will be used to summarize electrophysiological parameters at baseline and each post-baseline analysis visit, and the differences in electrophysiological parameters from baseline at each post-baseline analysis visit by randomization groups.

A line chart will be plotted to display the electrophysiological parameters at baseline and each post-baseline analysis visit, as well as the differences from baseline by randomization groups.

The difference in electrophysiological parameters from baseline at Month 15 will be analyzed using the ANCOVA model, with baseline value as a covariate and group as a fixed effect. Missing values will be imputed using the CR method.

The test results of each electrophysiological parameter for subjects in the FAS will be listed.

5.2.7.8 Differences of EQ-5D Utility Index and EQ-VAS Scores from Baseline at Month 15

The number and percentage of subjects with different severities in each EQ-5D scale dimension at each analysis visit will be summarized by randomization groups.

Descriptive statistics will be used to summarize the EQ-5D single item, total score, EQ-5D utility index, and EQ-VAS score at baseline and each post-baseline analysis visit, and the differences in EQ-5D single item, total score, EQ-5D utility index, and EQ-VAS score from baseline at each post-baseline analysis visit by randomization groups.

The EQ-5D utility index will be obtained by looking up the table in Annex mmc1.xls of Reference [2]. For example, if a subject's EQ-5D scales "Mobility", "Self-Care", "Usual Activities", "Pain/Discomfort" and "Anxiety/Depression" at a visit have values for "I have slight problems in walking about", "I have no problems washing or dressing myself", "I have no problems doing my usual activities", "I have no pain or discomfort" and "I am not anxious or depressed", then state = 21111. According to the table, the corresponding EQ-5D utility index is 0.933958024.

The EQ-5D single item and total score refer to the severity levels of each item in the health system, with values assigned from 1 to 5 points, ranging from mild to severe. The total score is the sum of the scores for each item.

A line chart will be plotted to display the EQ-5D single item, total score, EQ-5D utility index, and EQ-VAS score at baseline and each post-baseline analysis visit, as well as the differences from baseline by randomization groups.

The difference in EQ-5D single item, total score, EQ-5D utility index, and EQ-VAS score from baseline at Month 15 will be statistically analyzed using the ANCOVA model, with baseline value as a covariate and group as a fixed effect. Missing values will be imputed using the CR method. The results of the EQ-5D scale for subjects in the FAS will be listed.

5.2.8 Analysis of Safety Endpoints

1) Adverse Event

All AEs will be coded using the Chinese version of MedDRA 24.1 or higher.

The counting rules followed when summarizing AEs are as follows:

- In the summary of the number of subjects at each level (overall, SOC, and PT) and corresponding incidences, if a subject has the same AE more than once, the AE with the highest severity grade will be used for calculation. In the summary of the number of events at each level (overall, SOC, PT), the actual number of occurrences will be counted.
- In the summary of the number of subjects with different severities at each level (overall, SOC, and PT) and corresponding incidences, if a subject has the same AE more than once, the AE with the highest severity grade will be used for calculation. In the summary of the number of events by severity at each level (overall, SOC, PT), the actual severity grading will be considered for counting.

The number of events, number and percentage of subjects with all AEs, TEAEs, TRAEs, SAEs, treatment emergent serious adverse events (TESAEs), treatment related serious adverse events (TRSAEs), TEAEs with CTCAE Grade ≥ 3, TRAEs with CTCAE Grade ≥ 3, TEAEs leading to permanent discontinuation, TRAEs leading to permanent discontinuation, TEAEs leading to withdrawal from the trial, TRAEs leading to withdrawal from the trial, TEAEs leading to death, TRAEs leading to death, significant adverse events, treatment emergent significant adverse events, treatment related significant adverse events will be summarized for actual treatment groups and pooled groups by SOC and PT.

The number of events, number and percentage of subjects with TEAEs, TRAEs, TRAEs with an incidence of ≥ 2%, 5% and 10%, TESAEs, TRSAEs, TEAEs with CTCAE Grade ≥ 3, TRAEs with CTCAE Grade ≥3, TEAEs leading to permanent discontinuation, TRAEs leading to permanent discontinuation, TEAEs leading to withdrawal from the trial, TRAEs leading to withdrawal from the trial, TEAEs leading to death, TRAEs leading to death, treatment emergent significant adverse events, and treatment related significant adverse events will be summarized for actual treatment groups and pooled groups by SOC and PT.

The number of events, number and percentage of subjects with TEAEs, TRAEs, TRAEs with an incidence of ≥ 2%, 5%, and 10%, TESAEs, and treatment emergent significant adverse events will be summarized for actual treatment groups and pooled groups by SOC, PT, and highest severity grade.

The occurrence of AEs in subjects in the SS will be listed.

2) Laboratory Tests

The results of laboratory test indicators (hematology, blood chemistry, and urinalysis) at each analysis visit and the changes from baseline in each post-baseline analysis visit will be summarized

according to the actual treatment group and analysis visit. For urinalysis, if it is a quantitative test, the results of each analysis visit and the changes from baseline at each post-baseline analysis visit will be summarized according to the actual treatment group and analysis visit. If it is a qualitative test, the number and percentage of subjects with different results at each analysis visit will be summarized. A shift table will be used to describe the changes in clinical significance of laboratory test results (normal, abnormal without clinical significance, abnormal with clinical significance, and not examined/unknown) from baseline to each post-baseline analysis visit.

The number and percentage of subjects with different blood pregnancy test results at each analysis visit will be summarized by actual treatment group, and the results of blood pregnancy tests will be listed.

All laboratory test results of subjects in the SS and the results of laboratory test items at each visit for subjects in the SS with corresponding abnormal changes will be listed. Abnormal changes refer to the change from normal at baseline to abnormal without clinical significance or with clinical significance post-baseline, or from abnormal without significance at baseline to abnormal with clinical significance post-baseline.

3) 12-lead ECG

A shift table will be used to describe the changes in clinical significance of 12-lead ECG results (normal, abnormal without clinical significance, abnormal with clinical significance, and not examined) from baseline to each post-baseline analysis visit.

The 12-lead ECG results of subjects in the SS and the 12-lead ECG results at each visit for subjects in the SS with abnormal changes will be listed. Abnormal changes refer to the change from normal at baseline to abnormal without clinical significance or with clinical significance post-baseline, or from abnormal without significance at baseline to abnormal with clinical significance post-baseline.

4) Vital Signs

The test results of various vital signs (temperature, pulse, systolic and diastolic blood pressure) at each analysis visit and the changes from baseline in each post-baseline analysis visit will be summarized according to the actual treatment group and analysis visit. A shift table will be used to describe the changes in clinical significance of vital sign results (normal, abnormal without clinical significance, abnormal with clinical significance, and not examined) from baseline to each post-baseline analysis visit.

All vital sign results of subjects in the SS and the vital sign results at each visit for subjects in the SS with abnormal changes will be listed. Abnormal changes refer to the change from normal at baseline to abnormal without clinical significance or with clinical significance post-baseline, or from abnormal without significance at baseline to abnormal with clinical significance post-baseline.

5) Physical Examination

A shift table will be used to describe the changes in clinical significance of physical examination results (normal, abnormal without clinical significance, abnormal with clinical significance, and not examined) from baseline to each post-baseline analysis visit.

The physical examination results of subjects in the SS and the results of physical examination items at each visit for subjects in the SS with corresponding abnormal changes will be listed. Abnormal changes refer to the change from normal at baseline to abnormal without clinical significance or with clinical significance post-baseline, or from abnormal without significance at baseline to abnormal with clinical significance post-baseline.

6. Interim Analysis

An Independent Data Monitoring Committee (IDMC) will be established in this study for the interim analysis. The composition, responsibilities, and implementation details of the IDMC will be specified in the IDMC Charter.

The final analysis of this study is planned to be performed after the last subject completes 15 months of treatment (or withdraws early) after randomization. The interim analysis will be conducted when half of the subjects (i.e., 88 subjects) have completed the 15-month ONLS assessment (or withdraw early) (approximately after 15 months). In the group sequential design, the Lan-Demets algorithm (Lan-Demets, 1983) will be applied with an O'Brien-Fleming α-spending function to control the overall Type I error rate at 0.05:

$$\alpha(t_k) = 2\left(1 - \Phi\left(\Phi^{-1}\left(1-\frac{\alpha}{2}\right)\Big/\sqrt{t_k}\right)\right)$$

Where t_k is the amount of information accumulated during the kth interim analysis, Φ is the standard normal distribution function, and Φ^{-1} is the quantile function of the standard normal distribution function. The cumulative α-spending function $\alpha(t_k)$ for the interim analysis is solely dependent on the amount of information accumulated at that time, ensuring that the significance level (*P*-value boundary) for each interim analysis is solely dependent on this amount of information.

If the *P* value of the interim analysis is ≤ 0.003, the trial can be stopped due to significant superiority.

If the *P* value of the interim analysis is > 0.003, a sample size re-estimation may be performed. The sample size re-estimation will be performed according to the promising zone method. The calculation formula for conditional power (CP) is as follows:

$$\mathrm{CP}(\hat{\delta}_1, z_1, c_g) = 1 - \Phi\left(\frac{c_g\sqrt{n_2} - z_1\sqrt{n_1}}{\sqrt{n_2 - n_1}} - \frac{z_1}{\sqrt{n_1}}\sqrt{n_2 - n_1}\right)$$

Where Z_1 is the Z-test statistic for interim analysis, Φ is the standard normal distribution function, n_2 is the total planned sample size (176 subjects), n_1 is the actual sample size for interim analysis, and $c_g = 1.9686$.

- If the CP is ≥ 80%, continue the trial according to the original sample size;
- If the CP falls within the promising zone range of 37% ≤ CP < 80%, the sample size will be re-estimated using the CP-based sample size re-estimation method, expanding the sample size $n_2^*$to achieve a CP of 80%, andn_2^* meeting the following conditions:

$$80\% = \Phi\left(\frac{z_1}{\sqrt{n_1}}\sqrt{n_2^* - n_1} - \frac{c_g\sqrt{n_2} - z_1\sqrt{n_1}}{\sqrt{n_2 - n_1}}\right)$$

  - If the CP falls into the range of 65%≤CP<80%, a CP value of 65% (equivalent to z_1=1.58467) will be used to calculate the new sample size n_2^*;
  - If the CP falls into the range of 50%≤CP<65%, a CP value of 55% (equivalent to z_1=1.45484) will be used to calculate the new sample size n_2^*;
  - If the CP falls into the range of 37%≤CP<50%, a CP value of 45% (equivalent to z_1=1.32918) will be used to calculate the new sample size n_2^*;
- If the CP is within the range of 20%≤CP<37%, the sponsor will assess whether to continue the trial with the aforementioned formula to re-estimate the sample size.
- If the CP is < 20%, it can be considered to stop the trial due to a lack of efficacy.

The final analysis will be conducted using Fisher's combined probability test once all subjects have completed the primary endpoint assessment, with a two-sided significance level of 0.049. Assuming that the *P* value corresponding to the primary statistical analysis of the estimand at the interim analysis is p_1, and the *P* value corresponding to the primary statistical analysis of the estimand using data from subjects not included in the interim analysis is p_2, the corresponding test statistic is:

$$\chi^2)=-2 * [\ln(p_1) + \ln(p_2)]$$

This statistic follows a chi-square distribution with 4 degrees of freedom.

The actual α-spending and significance level for each analysis will be adjusted for the exact sample size actually observed at the time of analysis.

7. Templates for Statistical Analysis Tables

Templates for statistical analysis tables will be provided separately in another document.

8. Modifications to the Analysis Plan in the Protocol

The following modifications have been made based on the analysis plan of the clinical protocol:

- The primary estimand and corresponding statistical analysis of the primary efficacy endpoint have been retrospectively added.
- The number of multiple imputations has been changed from 20 to 100.

9. References

1. Lan, K. K. G. and DeMets, D. L. (1983) Discrete sequential boundaries for clinical trials. Biometrika,70(3), 659-663.

2. Luo, N., Liu, G., Li, M., Guan, H., Jin, X. and Rand-Hendriksen, K. (2017). Estimating an EQ-5D-5L Value Set for China. Value Health, 20(4), 662-669.

10. SAS Code Reference

(1) Primary Analysis

Assuming that the cross-sectional SAS dataset containing ONLS scores from each analysis visit is DSIN, where USUBJID denotes a unique subject number, TRT01PN indicates randomization (1 = PXT3003 group, 0 = placebo group), and ONLS0-ONLS3 represent ONLS scores at each analysis visit (baseline, Month 6, Month 12, and Month 15), the SAS codes for multiple imputation in the primary analysis of the estimand are as follows:

```
PROC MI DATA=DSINNIMPUTE=100 SEED=8392857 MAXIMUM=12 12 12 12 MINIMUM=.0 0 0 0 MINMAXITER=1000000 OUT=MI_MONOTONE;

MCMC CHAIN=MULTIPLE INITIAL=EM(CONVERGE=0.001 MAXITER=100000)

IMPUTE=MONOTONE;

VAR TRT01PN ONLS0 ONLS1 ONLS2 ONLS3;

RUN;

DATA MI_MONOTONE1 ICE1 ICE2;

   SET MI_MONOTONE;
```

```
IF ICETYPE = "Premature termination of treatment due to poor efficacy as judged by the investigator" THEN OUTPUT ICE1;
ELSE IF ICETYPE IN ("Premature termination of treatment for reasons other than poor efficacy as judged by the investigator", "Use of prohibited treatments that affect efficacy assessment") THEN OUTPUT ICE2;
ELSE OUTPUT MI_MONOTONE1;
RUN;
PROC SORT DATA=MI_MONOTONE1;BY _IMPUTATION_;RUN;
PROC MI DATA=MI_MONOTONE1 NIMPUTE=1 SEED=51343672
MAXIMUM=. 12 12 12 12 MINIMUM=. 0 0 0 0 MINMAXITER=1000000
OUT=MI_MONOTONE2;
BY _IMPUTATION_;
CLASS TRT01PN;
VAR TRT01PN ONLS0 ONLS1 ONLS2 ONLS3;
MONOTONE REG(/DETAILS);
MNAR MODEL(ONLS1 ONLS2 ONLS3/MODELOBS=(TRT01PN='0'));
RUN;
DATA MI_MONOTONE3;
SET MI_MONOTONE2 ICE2;
RUN;
PROC SORT DATA=MI_MONOTONE3;BY _IMPUTATION_;RUN;
PROC MI DATA=MI_MONOTONE3 NIMPUTE=1 SEED=51343672
MAXIMUM=. 12 12 12 12 MINIMUM=. 0 0 0 0
MINMAXITER=1000000 OUT=MI_COMPLETE1;
BY _IMPUTATION_;
CLASS TRT01PN;
VAR TRT01PN ONLS0 ONLS1 ONLS2 ONLS3;
MONOTONE REG(/DETAILS);
RUN;
DATA MI_COMPLETE15;
SET MI_COMPLETE1(IN=A) ICE1(IN=B);
IFATHEN CHG=ONLS3-ONLS0;
ELSE IF B THEN CHG=COALESCE(ONLS3,ONLS2,ONLS1)-ONLS0;
BASE=ONLS0;
KEEP _IMPUTATION_ USUBJID TRT01PN CHG BASE;
RUN;
PROC SORT DATA=MI_COMPLETE15;BY _IMPUTATION_;RUN;
```

(2) Sensitivity analysis 1

```
PROC MI DATA=DSINNIMPUTE=100 SEED=8392857 MAXIMUM=12 12 12 12 MINIMUM=. 0 0 0 0 MINMAXITER=1000000 OUT=MI_MONOTONE;
MCMC CHAIN=MULTIPLE INITIAL=EM(CONVERGE=0.001 MAXITER=100000) IMPUTE=MONOTONE;
VAR TRT01PN ONLS0 ONLS1 ONLS2 ONLS3;
RUN;
DATA MI_MONOTONE1 ICE2;
   SET MI_MONOTONE;
   IF NCOMPLT = "Poor efficacy as judged by the investigator" THEN OUTPUT ICE2;
   ELSE OUTPUT MI_MONOTONE1;
RUN;
PROC SORT DATA=MI_MONOTONE1;BY _IMPUTATION_;RUN;
PROC MI DATA=MI_MONOTONE1 NIMPUTE=1 SEED=51343672
MAXIMUM=. 12 12 12 12 MINIMUM=. 0 0 0 0 MINMAXITER=1000000 OUT=MI_COMPLETE1;
   BY _IMPUTATION_;
   CLASS TRT01PN;
   VAR TRT01PN ONLS0 ONLS1 ONLS2 ONLS3;
   MONOTONE REG(/DETAILS);
RUN;
DATA MI_COMPLETE15;
   SET MI_COMPLETE1(IN=A) ICE2(IN=B);
   IFATHEN CHG=ONLS3-ONLS0;
   ELSE IF B THEN CHG=COALESCE(ONLS3,ONLS2,ONLS1)-ONLS0;
   BASE=ONLS0;
   KEEP _IMPUTATION_ USUBJID TRT01PN CHG BASE;
RUN;
PROC SORT DATA=MI_COMPLETE15;BY _IMPUTATION_;RUN;
```

(3) Supplementary Analysis 1

```
PROC MI DATA=DSINNIMPUTE=100 SEED=8392857 MAXIMUM=12 12 12 12 MINIMUM=. 0 0 0 0 MINMAXITER=1000000 OUT=MI_MONOTONE;
MCMC CHAIN=MULTIPLE INITIAL=EM(CONVERGE=0.001 MAXITER=100000)
IMPUTE=MONOTONE;
VAR TRT01PN ONLS0 ONLS1 ONLS2 ONLS3;
RUN;
DATA MI_MONOTONE1 ICE2;
```

```
    SET MI_MONOTONE;
    IF NCOMPLT = "Poor efficacy as judged by the investigator" THEN OUTPUT ICE2;
    ELSE OUTPUT MI_MONOTONE1;
RUN;
PROC SORT DATA=MI_MONOTONE1;BY _IMPUTATION_;RUN;
PROC MI DATA=MI_MONOTONE1 NIMPUTE=1 SEED=51343672
MAXIMUM=. 12 12 12 12 MINIMUM=. 0 0 0 0 MINMAXITER=1000000
OUT=MI_COMPLETE1;
    BY_IMPUTATION_;
    CLASS TRT01PN;
    VAR ONLS0 ONLS1 ONLS2 ONLS3;
    MONOTONE REG(/DETAILS);
    MNAR MODEL(ONLS1 ONLS2 ONLS3/MODELOBS=(TRT01PN='0'));
RUN;
DATA MI_COMPLETE15;
    SET MI_COMPLETE1(IN=A) ICE2(IN=B);
    IFATHEN CHG=ONLS3-ONLS0;
    ELSE IF B THEN CHG=COALESCE(ONLS3,ONLS2,ONLS1)-ONLS0;
    BASE=ONLS0;
    KEEP _IMPUTATION_ USUBJID TRT01PN CHG BASE;
RUN;
PROC SORT DATA=MI_COMPLETE15;BY _IMPUTATION_;RUN;
```

(4) Mixed Model for Repeated Measures (MMRM)

```
ODS OUTPUT LSMEANS=LSMEANS DIFFS=DIFFS(WHERE=(AVISITN=_AVISITN));
PROC MIXED DATA=ADXXX METHOD=REML;
CLASS USUBJID TRT01PN(REF='0') AVISITN;
MODEL CHG=BASE TRT01PN AVISITN TRT01PN*AVISITN/NOINT SOLUTION CL
ALPHA=0.05 DDFM=KENWARDROGER;
REPEATED AVISITN/SUBJECT=USUBJID TYPE=UN R;
LSMEANS TRT01PN*AVISITN/SLICE=AVISITN DIFF CL ALPHA=0.05;
RUN;
ODS OUTPUT CLOSE;
```

(5) Rate, Rate Difference and Their Two-sided 95% CIs

Assuming that the SAS dataset containing the frequency of ONLS response at the end of treatment for each randomization group is DSIN, where TRT01PN indicates randomization (1 = PXT3003 group, 0 = placebo group), RESPYN represents ONLS response at the end of treatment (1 = response, 2 = no response), and COUNT represents the corresponding frequency, the SAS codes for

obtaining the two-sided 95% CI for the rate based on the Clopper-Pearson method and the two-sided 95% CI for the rate difference using the Newcombe-Wilson method, and Fisher's exact test are as follows:

```
PROC SORT DATA=DSIN;BY TRT01PN;RUN;
ODS SELECT BINOMIALCLS;
PROC FREQ DATA=DSIN;
   BY TRT01PN;
   TABLES RESPYN/ALPHA=0.05 BINOMIAL(CL=CLOPPERPEARSON LEVEL='1');
   WEIGHT COUNT;
RUN;
ODS SELECT ALL;
ODS SELECT FISHERSEXACT PDIFFCLS;
PROC FREQ DATA=DSIN;
   TABLES TRT01PN*RESPYN/ALPHA=0.05 FISHER RISKDIFF(CL=NEWCOMBE);
   WEIGHT COUNT;
RUN;
ODS SELECT ALL;
```
