# Supplementary material for: A phase III clinical trial of PXT3003 in Chinese patients with Charcot-Marie-Tooth disease type 1A
Source: iScience. 2026 Aug 26;29(9):117284. doi: 10.1016/j.isci.2026.117284 (PMC13544322; doi:10.1016/j.isci.2026.117284)
Supplement: Supplementary file 3 [file mmc3.pdf]

A multicenter, randomized, double-blind, placebo-controlled phase III clinical trial protocol to assess the efficacy and safety of PXT3003 in patients with Charcot-Marie-Tooth Disease type 1A for 15 months

**Protocol No.**	TSL-CM-PXT3003-III
**Sponsor**	Genenet Pharmaceutical Co., Ltd
**Leading Site**	Peking University Third Hospital
**Principal Investigator**	Prof. Fan Dongsheng
**Participating Site**	The Third Xiangya Hospital of Central South University
**Co-Principal Investigator**	Zhang Ruxu, Director
**Data Management and Statistical Institute**	Nanjing Aikeman Medical Information & Technology Co., Ltd
**Contract Research Organization**	WuXi Clinical Development Services Co., Ltd.
**Version No.**	1.0
**Version Date**	March 18, 2021

The Sponsor has the copyright of this document. This document shall not be used, divulged, published or disclosed in any other form without permission

Trial Protocol Version and Change History

Version No.	Version Date	Revision/Addition
1.0	March 18, 2021	NA

Clinical Study Project Management Information

**Leading Institution for Clinical Study:**	**Peking University Third Hospital**
Principal Investigator:	Prof. Fan Dongsheng
Address:	No. 49 Huayuan North Road, Haidian District, Beijing
Tel.:	13701023871
**Participating Site:**	**The Third Xiangya Hospital of Central South University**
Co-Principal Investigator:	Zhang Ruxu, Director
Address:	No.138 Tongzipo Road, Yuelu District, Changsha, Hunan Province
Tel.:	18975172668
**Sponsor:**	**Genenet Pharmaceutical Co., Ltd**
Responsible Person:	Liu Rui
Address:	1-1-2108-3, North Zone, Financial and Trade Center, No. 6865, Asia Road, Tianjin Pilot Free Trade Zone (Dongjiang Free Trade Port Zone)
Tel.:	022-86343837
**Institution for Data Management and Statistical Analysis:**	**Nanjing Aikeman Medical Information & Technology Co., Ltd**
Responsible Person:	Chen Gang
Address:	Room 801, Building E, No. 10 Xinghuo Road, High-tech Park, High-tech Development Zone, Pukou District, Nanjing City, Jiangsu Province
Tel.:	17801091198
**Contract Research Organization:**	**WuXi Clinical Development Services Co., Ltd.**
Responsible Person:	Jiao Zhuangzhuang
Address:	19F, Tower A, SOHO Renaissance Plaza, No. 388, Madang Road, Shanghai
Tel.:	17731108660

Signature Page

We have read this clinical trial protocol (Protocol No.: TSL-CM-PXT3003-III; Version No.: 1.0; Version Date: March 18, 2021), confirmed this clinical trial protocol and agreed to conduct the clinical trial according to this protocol.

I will keep this protocol and related contents confidential.

Sponsor: Genenet Pharmaceutical Co., Ltd

Responsible Person (Signature): __________

Date of Signature: ________ (MM/DD/YYYY)

Declaration and Signature Page of Investigator

Investigator’s Statement:

(1) I agree to participate in and guide this clinical study in person.

(2) I have received the Investigator's Brochure (v1.0, March 18, 2021) and am aware of the relevant study of the investigational drug.

(3) I have read the clinical trial protocol and the study will be conducted in accordance with the ethical standards and scientific principles stated in the Declaration of Helsinki and GCP. I agree to conduct this clinical study in accordance with the design and provisions of this protocol, and modify the protocol only after the Sponsor has been notified. The protocol can be implemented only after the consent of the Ethics Committee, unless for the protection of the safety, rights and interests of the participant.

(4) As required by the GCP, I will ensure that all participants sign the Informed Consent Form before being engaged in the study.

(5) I will be responsible for making medical decisions related to clinical trials to ensure timely and appropriate treatment for adverse events during the trials, as well as the recording and reporting of serious adverse events in accordance with relevant national regulations.

(6) I guarantee that the data will be input into the medical record of this study in a true, accurate, complete and timely manner. I will cooperate with monitoring and audit from monitor or auditor designated by the Sponsor, and inspection and visit from drug regulatory authority, so as to ensure the quality of clinical trial.

(7) I promise to keep the subject information and related matters confidential. If I violate my commitment, I am willing to assume the resulting legal responsibility.

(8) I agree to disclose my name and occupation to the Sponsor and agree to disclose any expenses related to the clinical study upon request. I agree to prohibit commercial and economic behaviors related to this trial.

We have read this clinical trial protocol (Protocol No.: TSL-CM-PXT3003-III; Version No.: 1.0; Version Date: March 18, 2021), confirmed this clinical trial protocol and agreed to conduct the clinical trial according to this protocol. I will keep this protocol and related contents confidential.

Leading Institution for Clinical Study:

Principal Investigator (Signature):__________

Date of Signature: ________ (MM/DD/YYYY)

Declaration and Signature Page of Investigator

Investigator’s Statement:

(1) I agree to participate in and guide this clinical study in person.

(2) I have received the Investigator's Brochure (v1.0, March 18, 2021) and am aware of the relevant study of the investigational drug.

(3) I have read the clinical trial protocol and the study will be conducted in accordance with the ethical standards and scientific principles stated in the Declaration of Helsinki and GCP. I agree to conduct this clinical study in accordance with the design and provisions of this protocol, and modify the protocol only after the Sponsor has been notified. The protocol can be implemented only after the consent of the Ethics Committee, unless for the protection of the safety, rights and interests of the participant.

(4) As required by the GCP, I will ensure that all participants sign the Informed Consent Form before being engaged in the study.

(5) I will be responsible for making medical decisions related to clinical trials to ensure timely and appropriate treatment for adverse events during the trials, as well as the recording and reporting of serious adverse events in accordance with relevant national regulations.

(6) I guarantee that the data will be input into the medical record of this study in a true, accurate, complete and timely manner. I will cooperate with monitoring and audit from monitor or auditor designated by the Sponsor, and inspection and visit from drug regulatory authority, so as to ensure the quality of clinical trial.

(7) I promise to keep the subject information and related matters confidential. If I violate my commitment, I am willing to assume the resulting legal responsibility.

(8) I agree to disclose my name and occupation to the Sponsor and agree to disclose any expenses related to the clinical study upon request. I agree to prohibit commercial and economic behaviors related to this trial.

We have read this clinical trial protocol (Protocol No.: TSL-CM-PXT3003-III; Version No.: 1.0; Version Date: March 18, 2021), confirmed this clinical trial protocol and agreed to conduct the clinical trial according to this protocol. I will keep this protocol and related contents confidential.

Clinical Study Site:

Investigator (signature):___________

Date of Signature: ________ (MM/DD/YYYY)

Signature Page

We have read this clinical trial protocol (Protocol No.: TSL-CM-PXT3003-III; Version No.: 1.0; Version Date: March 18, 2021), confirmed this clinical trial protocol and agreed to conduct the clinical trial according to this protocol.

I will keep this protocol and related contents confidential.

Data Management and Statistical Institute:

Responsible Person (Signature) ________________

Date of Signature: ________ (MM/DD/YYYY)

Signature Page

We have read this clinical trial protocol (Protocol No.: TSL-CM-PXT3003-III; Version No.: 1.0; Version Date: March 18, 2021), confirmed this clinical trial protocol and agreed to conduct the clinical trial according to this protocol.

I will keep this protocol and related contents confidential.

Contract Research Organization:

Responsible Person (Signature) ________________

Date of Signature: ________ (MM/DD/YYYY)

Table of Contents

Trial Protocol Version and Change History ... 2
Clinical Study Project Management Information ... 3
Signature Page ... 4
Table of Contents ... 9
Protocol Synopsis ... 11
Trial Flow Chart ... 16
List of Abbreviations ... 19
1. Background ... 21
2. Information on Investigational Drug [8] ... 22
2.1 Mechanism of Action ... 23
2.2 Preclinical Study Information ... 24
3. Study Objectives ... 32
4. Trial Design ... 32
4.1 Overall Design ... 32
4.2 Justification of Trial Design ... 33
4.3 Randomization ... 33
4.4 Blinding ... 33
4.5 Determination of Sample Size ... 33
4.6 Selection of Control Drug ... 34
4.7 Design of Dose Group ... 34
5. Study Population ... 35
5.1 Diagnostic Criteria ... 35
5.2 Inclusion Criteria ... 35
5.3 Exclusion Criteria ... 35
5.4 Dropout/Withdrawal Criteria ... 36
6. Investigational Drug ... 36
6.1 Basic Information of Investigational Drug ... 36
6.2 Package and Label of the Drug ... 37
6.3 Storage, Management and Distribution of Investigational Drug ... 37
6.4 Medication Compliance ... 37
6.5 Provisions for Concomitant Medication ... 37
7. Trial Procedures ... 38
7.1 Screening Visit/Inclusion Visit (D-30 to D-1) ... 38
7.2 Trial Period (D1–D450) ... 39
7.3 Early Withdrawal ... 40
7.4 Unscheduled Visit ... 40
8. Trial Evaluation Indicators ... 40
8.1 Efficacy Indicators ... 40
8.2 Safety Evaluation Indicators ... 43
9. Adverse Events and Serious Adverse Events ... 43

9.1 Definition of Adverse Event......43
9.2 Treatment Emergent Adverse Event......44
9.3 Determination Criteria for Severity of Adverse Events......44
9.4 Judgment Criteria for Relationship between Adverse Events and Investigational Drug......44
9.5 Identification and Follow-up of Adverse Events......45
9.6 Requirements for Reporting Adverse Events......45
9.7 Definition of Serious Adverse Event......45
9.8 Requirements for Reporting Serious Adverse Events......45
9.9 Suspected Unexpected Serious Adverse Reaction......46
9.10 Reporting of Pregnancy......46

10. Risk Management and Risk Assessment......47

10.1 Adverse Reactions of Individual Pharmaceutical Ingredients in PXT3003......47
10.2 Safety Report during Drug Development......48
10.3 Risk Management Plan......51

11. Data Management......52

11.1 Data Collection......52
11.2 Database Management and Quality Control......52
11.3 Data Review and Monitoring of Study Site......52

12. Statistical Analysis......53

12.1 Statistical Hypothesis......53
12.2 Definition of Analysis Population......53
12.3 Statistical Methods......53
12.4 Interim Analysis......54
12.5 Statistical Software......55

13. Ethical Norms and Informed Consent......55

13.1 Ethics Committee......55
13.2 Informed Consent......55
13.3 Subject Confidentiality......55

14. Quality Control and Assurance......56

15. Handling of Special Conditions during the Trial......56

15.1 Termination Criteria......56
15.2 Protocol Deviations or Violations......56

16. Protocol Revision......56

17. Data Archiving......57

18. References......57

Appendices......59

Protocol Synopsis

<table>
<tr><td>Name of Investigational Drug</td><td>PXT3003</td></tr>
<tr><td>Registration Category</td><td>Chemical Drug Class 2.3</td></tr>
<tr><td>Trial Name</td><td>A multicenter, randomized, double-blind, placebo-controlled phase III clinical trial to assess the efficacy and safety of PXT3003 in patients with Charcot-Marie-Tooth Disease type 1A for 15 months</td></tr>
<tr><td>Study Objectives</td><td>Primary Objective:
To assess the efficacy of PXT3003 on Charcot-Marie-Tooth Disease type 1A (CMT1A) measured by the Overall Neuropathy Limitation Scale (ONLS) scores in patients treated for 15 months.
Secondary Objective:
To assess the efficacy of PXT3003 on functional tests, electrophysiological parameters, and quality of life measures, and assess the safety of PXT3003.</td></tr>
<tr><td>Overall Design</td><td>Randomized, double-blind, placebo-controlled, and multicenter</td></tr>
<tr><td>Sample Size</td><td>This study enrolls a total of 176 subjects, 88 subjects in the investigational drug group, and 88 subjects in the placebo group (1:1).
Assuming that the difference in ONLS score improvement from baseline between the investigational drug group and the control group is 0.3 and the standard deviation is 0.6, type I error is controlled at 0.05 (two-sided), and at least 80% power is given to test the superiority of the investigational drug group over the placebo group, 70 evaluable subjects are required for each group. According to the dropout rate of 20%, 88 subjects are planned to be included in each group.</td></tr>
<tr><td>Information of Investigational Drug</td><td>1 Investigational drug: PXT3003 oral solution
Strength: 100 ml/vial, containing (RS)-baclofen 60 mg, naltrexone hydrochloride 7 mg, and D-sorbitol 2100 mg
Storage: packed in brown glass vials, stored at 2°C–8°C.
2 Placebo: PXT3003 oral solution placebo
Strength: 100 ml/vial
Storage: packed in brown glass vials, stored at 2°C–8°C.
The investigational drug and placebo are provided by Genenet Pharmaceutical Co., Ltd</td></tr>
<tr><td>Grouping</td><td>The study is divided into two groups: the investigational drug group and the placebo group. Administration: twice daily, oral with meals (breakfast and dinner), 5 ml/dose in the first 2 weeks (D1–D14), and thereafter 10 ml/dose (D15–D450).
<table><tr><td>Group</td><td>Dose and Frequency</td><td>Course of Treatment</td><td>Number of Subjects</td></tr><tr><td>Investigational drug group</td><td>10 ml/dose BID</td><td>15 months</td><td>88 subjects</td></tr><tr><td>Placebo group</td><td>10 ml/dose BID</td><td>15 months</td><td>88 subjects</td></tr></table></td></tr>
<tr><td>Study Process</td><td>Screening Visit/Inclusion Visit (D-30 to D-1)
Signing the informed consent form.
D-30 to D-2: Screening Visit
PMP22 gene test will be performed first. Other screening examinations will be performed once CMT1A is diagnosed, including demographic data, living habits, medical history, treatment history, allergic history, height, weight, vital signs, physical examination, chest radiography (AP view) or chest CT plain scan, abdominal B-ultrasound, virological screening, hematology, blood chemistry (hepatic and renal function, electrolytes, fasting blood glucose, and glycated hemoglobin), urinalysis, female blood pregnancy test, Overall Neuropathy Limitation Scale (ONLS) score, Charcot-Marie-Tooth Neuropathy Score second version (CMTNS-V2), and electrophysiological examination.
D-1: Inclusion Visit Period
10-meter walking test (10MWT), nine hole peg test (9HPT) with the non-dominant</td></tr>
</table>

	hand, quantified muscular testing (QMT), 12-lead ECG, ONLS score, CMTNS-V2 score, electrophysiological examination and quality of life evaluation (EQ-5D), and laboratory tests (hematology, blood chemistry, urinalysis, female blood pregnancy test) will be performed.<br>If ONLS score, CMTNS-V2 score, and electrophysiological examination are performed within 14 days before randomization, and laboratory tests (hematology, blood chemistry, urinalysis, female blood pregnancy test) are performed within 7 days before randomization, re-evaluation is not required on D-1, and inclusion & exclusion criteria should be re-checked. The subjects who meet all inclusion criteria and none of the exclusion criteria can participate in the study. Those who pass the screening will be randomized, and provided with drugs and diary cards<br>**Trial Period (D1–D450)**<br>D1: The day of the first dose.<br>A total of 11 call visits will be conducted in this study on D13 ± 1, D30 ± 7, D60 ± 7, D120 ± 7, D150 ± 7, D210 ± 7, D240 ± 7, D300 ± 7, D330 ± 7, D390 ± 7 and D420 ± 7, respectively, to collect the information on medication, adverse events and concomitant medications of subjects;<br>D13±1: The investigator should remind the subjects that the dosage is changed from "5 ml/time" to "10 ml/time", and the frequency of administration (twice daily) and oral administration with meals (breakfast and dinner) remain unchanged.<br>Visit 1 (V1, D90 ± 10)<br>(1) Collect remaining drugs and diary cards;<br>(2) Collect adverse events;<br>(3) Vital signs, physical examination, 12-lead ECG hematology, blood chemistry, female blood pregnancy test and urinalysis;<br>(4) Record concomitant medication and treatment;<br>(5) Distribute drugs and diary cards.<br>Visit 2 (V2, D180 ± 10)<br>(1) Collect remaining drugs and diary cards;<br>(2) Collect adverse events;<br>(3) Vital signs, physical examination, 12-lead ECG hematology, blood chemistry, female blood pregnancy test and urinalysis;<br>(4) Record concomitant medication and treatment;<br>(5) Distribute drugs and diary cards;<br>(6) ONLS scores, CMTNS-V2 scores, 9HPT, QMT, 10 MWT, electrophysiological test and EQ-5D.<br>Visit 3 (V3, D270 ± 10)<br>(1) Collect remaining drugs and diary cards;<br>(2) Collect adverse events;<br>(3) Vital signs, physical examination, 12-lead ECG hematology, blood chemistry, female blood pregnancy test and urinalysis;<br>(4) Record concomitant medication and treatment;<br>(5) Distribute drugs and diary cards.<br>Visit 4 (V4, D360 ± 10)<br>(1) Collect remaining drugs and diary cards;<br>(2) Collect adverse events;<br>(3) Vital signs, physical examination, 12-lead ECG hematology, blood chemistry, female blood pregnancy test and urinalysis;<br>(4) Record concomitant medication and treatment;<br>(5) Distribute drugs and diary cards;<br>(6) ONLS scores, CMTNS-V2 scores, 9HPT, QMT, 10 MWT, electrophysiological test and EQ-5D.<br>Visit 5 (V5, D450 ± 10)<br>(1) Collect remaining drugs and diary cards;<br>(2) Collect adverse events;

	(3) Vital signs, physical examination, 12-lead ECG hematology, blood chemistry, female blood pregnancy test and urinalysis;<br>(4) Record concomitant medication and treatment;<br>(5) ONLS scores, CMTNS-V2 scores, 9HPT, QMT, 10 MWT, electrophysiological test and EQ-5D.
**Diagnostic Criteria**	The diagnosis of CMT1A refers to the following criteria:<br>(1) One of the following clinical manifestations: ① slowly progressive muscle weakness and atrophy of the distal extremities; ② peripheral sensory disturbance, diminished or absent tendon reflexes; ③ deformity: high arched feet, crane leg deformity, and scoliosis.<br>(2) Family genetic history: ① clear family history is an important basis for the diagnosis of CMT; ② absence of family history cannot rule out CMT.<br>(3) Other causes of chronic peripheral neuropathy, such as diabetes, autoimmune diseases or poisoning, are excluded.<br>(4) Neurophysiological examination: ulnar nerve motor nerve conduction velocity > 15 m/s;<br>(5) Genetic diagnosis: *PMP22* gene duplication.
**Inclusion Criteria**	Subjects must meet all of the following inclusion criteria to be included in the study:<br>(1) Male or female, aged 16–65 years (inclusive);<br>(2) Subjects with a proven genetic diagnosis of CMT1A (PMP22 duplication on chromosome 17p11.2);<br>(3) Subjects with CMTNS-V2 scores >2 and ≤ 18;<br>(4) Subjects with muscle weakness in at least foot dorsiflexion (clinical assessment);<br>(5) Subjects with ulnar nerve motor nerve conduction velocity > 15 m/s;<br>(6) Subjects who provide signed informed consent forms to voluntarily participate in the clinical trial, and are able to understand and comply with all study procedures.
**Exclusion Criteria**	Subjects who meet any of the following are not eligible for the trial:<br>(1) Subjects with known hypersensitivity to (RS)-baclofen, naltrexone hydrochloride, D-sorbitol or PXT3003 excipients, or history of other serious allergies;<br>(2) Subjects with contraindications to use of baclofen, naltrexone, or sorbitol, such as porphyria;<br>(3) Subjects with any other associated cause of peripheral neuropathy such as diabetes (including a history of diabetes and glycated hemoglobin > 6.5%);<br>(4) Subjects with another nervous system disorders that affect the evaluation of study treatment;<br>(5) Subjects with ONLS score of 0;<br>(6) Subjects with a clinically significant history of an unstable medical illness (unstable angina pectoris, tumor, hematological diseases, hepatitis or liver failure, renal failure, etc.) in the past 1 year that may jeopardize the participation in the study;<br>(7) Subjects who received limb surgery within six months before randomization or planned before completion of the clinical trial;<br>(8) Subjects with hepatic or renal dysfunction:<br>a. TBIL>1.5×ULN, ALT>3×ULN, AST>3×ULN;<br>b. Cr>1.5×ULN;<br>(9) Subjects with positive syphilis antibody and HIV antibody;<br>(10) Subjects with tumors suggested by chest X-ray or B ultrasound;<br>(11) Subjects with a history of alcohol dependence in the past 3 months;<br>(12) Pregnant or lactating women, and subjects who cannot use appropriate contraceptive measures during the trial;<br>(13) Subjects who used concomitant treatment 4 weeks prior to enrollment, including but not limited to baclofen, naltrexone, sorbitol, opioids, vitamin C, levothyroxine, and potentially neurotoxic drugs (e.g., amiodarone, chloroquine);<br>(14) Subjects unable to complete study follow-up;<br>(15) Subjects who have participated in another investigational drug trial and used the

	investigational drug in the past 30 days;<br>(16) If a subject has already been included in this study, it will not be possible to include another one from the same family and living in the same household to avoid confusion in treatment, and the affection on blind treatment and interpretation of study results;<br>(17) Subjects who are not suitable for participation in this clinical trial due to poor compliance or other factors as judged by the investigator.
**Dropout/Withdrawal Criteria**	(1) Subjects who withdraw from the study of their own accord and withdraw their informed consent forms;<br>(2) Subjects who cannot tolerate adverse events;<br>(3) Females who become pregnant during the study;<br>(4) Other reasons that the investigator considers the subjects are unable to continue the trial;<br>(5) Subjects with poor compliance (failure to take prescribed medications and receive clinical evaluation; other behaviors that seriously affect the trial results).
**Termination Criteria**	Under the premise of fully protecting the rights & interests and safety of subjects, the Sponsor, Ethics Committee or regulatory authorities require the termination of the study.
**Evaluation Indicators**	**Primary Efficacy Indicator:**<br>Improvement in Overall Neuropathy Limitations Scale (ONLS) scores, defined as the difference in ONLS scores from baseline at Month 15.<br>**Secondary Efficacy Indicator:**<br>(1) Treatment response rate for PXT3003;<br>(2) Arm and leg subscale scores in ONLS;<br>(3) Charcot-Marie-Tooth Neuropathy Score second version (CMTNS-V2) total scores and subscale scores;<br>(4) Time of 10-meter walking test (10 MWT);<br>(5) Nine hole peg test (9HPT) with the non-dominant hand;<br>(6) Quantified muscular testing (QMT) (grip strength and foot dorsiflexion);<br>(7) Electrophysiological parameters of sensory and motor nerve conduction of the ulnar and radial nerves, including:<br>- Amplitude of ulnar nerve compound muscle action potential (CMAP)<br>- Amplitude of radial nerve sensory nerve action potential (SNAP)<br>- Ulnar and radial nerve conduction velocity (NCV)<br>- Ulnar and radial nerve distant motor limit (DML)<br>(8) Quality of life evaluation<br>- EuroQol-5 dimension (EQ-5D) evaluation<br>**Safety Evaluation Indicators:**<br>(1) Changes in physical examination, vital signs, laboratory tests and 12-lead ECG;<br>(2) Incidence, relationship, and severity of treatment emergent adverse events (TEAEs);<br>(3) Incidence of TEAEs of Grade ≥ 3;<br>(4) Incidence of adverse events (AEs) leading to investigational drug discontinuation;<br>(5) Incidence of serious adverse events (SAEs).
**Statistical Methods**	**Analysis of Primary Efficacy Indicator:**<br>The primary efficacy endpoint is defined as the difference in the ONLS score at Month 15 relative to baseline. The primary efficacy endpoint is analyzed using an ANCOVA model with covariates including baseline value and fixed effects including treatment group. The main effect of the treatment group will be reported along with its 95% confidence interval and p-value. Descriptive statistics (mean and standard deviation) will also be used to summarize the baseline value, value at each visit and change from baseline of ONLS score in each group.<br>**Analysis of Secondary Efficacy Indicator:**<br>The same analysis method as for the primary efficacy endpoint will be used for the difference in ONLS score at Month 6 and Month 12 relative to baseline.<br>The Clopper-Pearson method is used to calculate the 95% CI of the two response rates, and the chi-square test is used to compare the two groups.

	The statistical analysis method as for the primary efficacy indicator will be applied to the following secondary efficacy indicators: arm and leg subscale scores in ONLS, Charcot-Marie-Tooth Neuropathy Score second version (CMTNS-V2) total score, CMTNS-V2 arm item score, CMTNS-V2 leg item score; time required for 10-meter walking test (10MWT); time required for nine hole peg test (9HPT); quantified muscular testing (QMT) (grip strength and foot dorsiflexion), electrophysiological parameters of sensory and motor nerve conduction of the ulnar and radial nerves.<br>The quality of life of patients will be assessed by the EuroQol-5 Dimension (EQ-5D) and EQ-VAS, and the scores of EQ-5D and EQ-VAS at each visit and their changes from baseline will be statistically described.<br>**Analysis of Safety Indicators:**<br>Adverse events will be coded according to the provisions of the MedDRA and will be graded according to the Common Terminology Criteria for Adverse Events (CTCAE) V5.0. Adverse events that occur during or after the administration of the investigational drug, including treatment emergent adverse events (TEAEs), investigational drug-related TEAEs, TEAEs of grade 3 or above, TEAEs leading to treatment discontinuation, TEAEs leading to death, serious adverse reactions, serious adverse events (SAEs), and investigational drug-related SAEs, will be summarized for the number of patients and percentage by system organ classification (SOC), preferred term (PT) and group. In addition, the severity of TEAEs will also be summarized by SOC, PT and group. The proportion of subjects with at least 1 adverse event (multiple consecutive occurrences of the same event will be counted as 1 event by maximum severity) will be reported.<br>Deaths reported during study treatment and in the follow-up period after treatment completion/discontinuation will be summarized.<br>The test values and changes from the baseline of safety indicators including specific laboratory tests, physical examination, vital signs and ECG will be summarized. Where applicable, the baseline examination results and the examination results at various time points after baseline should be presented in a cross table.<br>**Interim Analysis:**<br>An Independent Data Monitoring Committee (IDMC) will be established in this study for the interim analysis. The composition, responsibilities, and implementation details of the IDMC will be specified in the IDMC Charter.<br>The interim analysis will be conducted when 88 subjects complete the 15-month ONLS assessment for the purpose of a superiority test of ONLS scores and sample size re-estimation. An increase in sample size may be considered if deemed necessary by the IDMC. The Lan-Demets approach is used in the Group Sequential Design, and O'Brien-Fleming α-spending function is used to control the overall type I error rate at 0.05: if the p-value of the interim analysis is ≤ 0.003, the trial will be stopped for significant superiority. If the p-value of the interim analysis is > 0.003, the trial continues. The final analysis will be performed when all subjects complete the assessment of the primary endpoint at a significance level of 0.049.<br>The actual α-spending and significance level for each analysis will be adjusted for the exact sample size actually observed at the time of analysis.
**Trial Schedule**	**May 2021–May 2024**

Trial Flow Chart

Period	Screening/Baseline Period		Trial Period [p)]				
Visit	Screening Visit	Inclusion Visit	Visit 1	Visit 2	Visit 3	Visit 4	Visit 5
Time	D-30~D-2	D-1	Month 3<br>D90±10	Month 6<br>D180±10	Month 9<br>D270±10	Month 12<br>D360±10	Month 15<br>D450±10
Signing of informed consent form	×						
PMP22 gene test [a)]	×						
Demographic data [b)]	×						
Living habits [c)]	×						
Medical history, treatment history, allergy history [d)]	×						
Height and weight	×						
Vital signs [e)]	×	×	×	×	×	×	×
Physical examination [f)]	×	×	×	×	×	×	×
12-lead ECG		×	×	×	×	×	×
Anteroposterior chest X-ray or chest CT plain scan	×						
Abdominal ultrasound [g)]	×						
Viral test [h)]	×						
Blood routine [i)]	×	×[n)]	×	×	×	×	×
Blood chemistry [j)]	×	×[n)]	×	×	×	×	×
Urinalysis [k)]	×	×[n)]	×	×	×	×	×
Female blood pregnancy test	×	×[n)]	×	×	×	×	×
ONLS	×	×[o)]		×		×	×
CMTNS-V2	×	×[o)]		×		×	×
Electrophysiological test (non-dominant side) [l)]	×	×[o)]		×		×	×
10MWT		×		×		×	×
9HPT (non-dominant side)		×		×		×	×
QMT (foot dorsiflexion and grip strength)		×		×		×	×
EQ-5D		×		×		×	×

Period	Screening/Baseline Period		Trial Period p)				
Visit	Screening Visit	Inclusion Visit	Visit 1	Visit 2	Visit 3	Visit 4	Visit 5
Time	D-30~D-2	D-1	Month 3<br>D90±10	Month 6<br>D180±10	Month 9<br>D270±10	Month 12<br>D360±10	Month 15<br>D450±10
Review of inclusion/exclusion criteria	×	×					
Randomization		×					
Dispensing of drugs		×	×	×	×	×	
Recovery of investigational drug			×	×	×	×	×
Issuance of diary cards		×	×	×	×	×	
Recovery of diary cards			×	×	×	×	×
Recording of adverse events	×	×	×	×	×	×	×
Record of concomitant medication m)	×	×	×	×	×	×	×
Concomitant therapy	×	×	×	×	×	×	×

a) A proven genetic test report of CMT1A prior to the study is acceptable.
b) Demographic data include name, gender, date of birth (age), ethnicity, marriage and childbearing status.
c) Living habits include smoking and drinking history.
d) Medical history, treatment history and allergic history include past medical history and treatment history of major diseases, present medical history and treatment history of major diseases, history of allergy to drugs/foods, etc., and history of infectious diseases. Medical history in the past 1 year and the history of drug use 3 months before enrollment should be collected.
e) Vital signs include body temperature (axillary), sitting blood pressure and pulse.
f) Physical examinations include skin and mucosa, lymph nodes, head and neck (eyes, oropharynx, thyroid), chest (heart, respiratory system), abdomen, musculoskeletal system and nervous system.
g) Abdominal ultrasound includes liver, gallbladder, pancreas, spleen, and both kidneys.
h) Viral test includes hepatitis B surface antigen, hepatitis B surface antibody, hepatitis B e antigen, hepatitis B e antibody, hepatitis B core antibody, hepatitis C antibody, treponema pallidum antibody and human immunodeficiency virus antibody.
i) Hematology includes WBC, RBC, hemoglobin, platelet, neutrophil and lymphocyte count.
j) Blood chemistry includes alanine aminotransferase, aspartate aminotransferase, alkaline phosphatase, glutamyl transpeptidase, total protein, albumin, total bilirubin, direct bilirubin, creatinine, urea or urea nitrogen, fasting blood glucose, potassium, sodium, chloride and glycated hemoglobin.
k) Urinalysis includes urine protein, urine glucose, urine WBC and urine RBC.
l) Electrophysiological tests include ulnar nerve compound muscle action potential, radial nerve sensory nerve action potential, ulnar and radial nerve conduction velocity, as well as ulnar and radial nerve distant motor limit.
m) Record of concomitant medication: Concomitant medication from 3 months before enrollment to the last visit.
n) If relevant laboratory results 7 days before the randomization are available, re-assessment is not required on D-1.
o) If ONLS score, CMTNS score and electrophysiological test result 14 days before the randomization are available, re-assessment is not required on D-1.

p) In the trial period, a total of 11 call visits will be conducted on D13 ± 1, D30 ± 7, D60 ± 7, D120 ± 7, D150 ± 7, D210 ± 7, D240 ± 7, D300 ± 7, D330 ± 7, D390 ± 7, and D420 ± 7, respectively, to collect the information on medication, adverse events and concomitant medications of subjects. On D13 ± 1, the investigator will remind the subjects that the dosage is changed from "5 ml/time" to "10 ml/time".

Note: The screening visit and the inclusion visit may be completed on the same day.

List of Abbreviations

Abbreviation	Description
ATC	Anatomical Therapeutic Chemical
AE	Adverse Event
BID	Bis In Die
cAMP	Cyclic Adenosine Monophosphate
CMAP	Compound Muscle Action Potential
CMT	Charcot-Marie-Tooth
CMT1A	Charcot-Marie-Tooth Disease type 1A
CMTNS	Charcot-Marie-Tooth Neuropathy Score
C_{max}	Maximum Concentration
CRF	Case Report Form
CTCAE	Common Terminology Criteria for Adverse Event
DM	Date Manager
DML	Distant Motor Limit
DMP	Date Management Plan
DRG	Dorsal Root Ganglia
DVP	Data Verification plan
eCRF	Electronic Case Report Form
ECG	Electrocardiograph
EDC	Electronic Date Capture
ENMC	The European Neuromuscular Centre
EQ-5D	EuroQol-5 Dimension
FAS	Full Analysis Set
FDA	Food and Drug Administration
GCP	Good Clinical Practice
GABA	΄Y-Aminobutyric Acid
GABA-B	Metabotropic Transmembrane Receptor for GABA
Gαi	Inhibited Type G protein Alpha Subunit
GCP	Good Clinical Practice
GPCRs	G Protein-Coupled Receptors
HMSN	Hereditary Motor and Sensory Neuropathy
9HPT	Nine Hole Peg Test
IB	Investigator’s Brochure
ICH	International Council on Harmonisation of Technical Requirements for Pharmaceuticals for Human Use
IDMC	Independent Data Monitoring Committee
IWRS	Interactive Web Response System
MedDRA	Medical Dictionary for Regulatory Activities
mRNA	Messenger RNA
min	Minutes
10MWT	10 meter Walking Test
NCV	Nerve Conduction Velocity
ONLS	Overall Neuropathy Limitation Scale
PK	Pharmacokinetics
PMP22	Peripheral Myelin Protein 22
PPS	Per Protocol Set
PT	Preferred Term
PXT3003	Fixed Dose Combination of Baclofen, Naltrexone and Sorbitol

Abbreviation	Description
RT-PCR	Reverse Transcription Polymerase Chain Reaction
QD	Quaque Die
QC	Quality Control
QMT	Quantified Muscular Testing
SAE	Serious Adverse Event
SAS	Statistical Analysis System
SD	Standard Deviation
SDV	Source Data Verification
SNAP	Sensory Nerve Action Potential
SOC	System Organ Class
SOP	Standard Operating Procedure
SS	Safety Set
SUSAR	Suspected Unexpected Serious Adverse Reaction
$t_{1/2}$	Terminal Elimination Half-life
T_{max}	Time of Maximum Concentration
TG	Transgenic
TEAE	Treatment Emergent Adverse Event
Vd	Apparent Volume of Distribution
VAS	Visual Analogue Scale
WHO	World of Health Organization

1. Background

Charcot-Marie-Tooth disease (CMT), first reported by Charcot, Marie, and Tooch in 1886, also known as Hereditary Motor and Sensory Neuropathy (HMSN), is the most common monogenic genetic disease of the peripheral nervous system in clinical practice, with high clinical heterogeneity and genetic heterogeneity. With a global incidence of about 1/2500 (0.4‰), CMT is included in the list of rare diseases in Europe and the United States. CMT was included in China's First List of Rare Diseases on May 11, 2018. The meta-analysis results revealed [1] that the incidence of CMT in Europe and the United States was 9.7–82.3/100000 (0.097%–0.823‰), and the incidence in Japan was about 10.8/100000 (0.11‰). Charcot-Marie-Tooth disease type 1A (CMT1A) accounts for 40%–50% of CMT in Europe and the United States [2], 23.3% in Japan [3], and 13.5%–26.1% in China [4,5]. Since there are currently no epidemiological data on CMT in China, 20,000–40,000 CMT1A patients in China are expected based on the incidence of CMT in Japan.

Genetic analysis found that CMT1A was induced by gene duplication of peripheral myelin protein 22 (PMP22) on chromosome 17p11.2 [2]. The main function of PMP22 is to maintain the integrity of myelin structure and regulate the proliferation of nerve cells. PMP22 gene duplication leads to an increase in PMP22, disrupting normal myelination associated with neurons and Schwann cells, slowing down signaling along axons, and affecting the normal function of nerves.

Clinical characteristics of CMT1A [2]: It usually occurs before the age of 20 years, and then it progresses slowly, with mild to moderate limb motor and sensory impairment, manifested as chronic progressive distal limb muscle weakness and muscular atrophy, hypoesthesia and weakened or absent tendon reflexes, accompanied by skeletal deformities such as pes cavus and scoliosis. The symptoms of lower limbs are foot drop, hammer toes, pes cavus, "crane legs", high knee lift gait, and frequent stumbles or falls. Some patients cannot walk without equipment or a wheelchair. The symptoms of the upper limbs are clawed hands, fine movement difficulties, and sensory impairments that are usually distributed in a sock-glove pattern. Other manifestations: mild to severe musculoskeletal pain in the four limbs, and sprains and fractures due to foot deformities and gait disorders. These symptoms seriously affect the daily life, quality of life, and mental health of patients with CMT1A. Symptoms vary considerably between patients and even within the same family.

There are currently no effective therapeutic drugs and treatment guidelines for CMT1A. It is mainly managed by symptomatic and supportive treatments, including the use of orthopaedic shoes, surgical orthopaedic surgery, neurotrophic and pain treatment, and psychological counseling, to maximize the recovery of independent mobility, improve quality of life and reduce disability.

For CMT1A, PHARNEXT company (France) screened the marketed drugs by using the network pharmacology method and developed PXT3003, a fixed-dose combination of three drugs: (RS)-baclofen, naltrexone hydrochloride and D-sorbitol, of which the dose of three drugs was 1/35–1/5 of the approved dose. PXT3003 can act on Schwann cells and neurons in different ways to down-regulate the expression of PMP22 and is intended to treat CMT1A.

The three main ingredients of PXT3003 have been marketed for many years. (RS)-baclofen was approved by FDA in 1977 for the treatment of skeletal muscle spasms induced by different diseases, and was marketed in China in 1998 at a maximum approved dose of 75 mg/d; naltrexone hydrochloride was approved by the FDA in 1984 for the treatment of opioid and alcohol dependence and was marketed in China in 2000 at a maximum approved dose of 50 mg/d; D-sorbitol was approved by the FDA in 1978 for the treatment of constipation (sorbitol irrigant), and is widely used worldwide as a medical excipient and food additive, with an approved dose of 12 g/d. In terms of safety, the three drugs did not show serious adverse reactions when used alone in clinical practice (no boxed warning).

The results of non-clinical studies with PXT3003 revealed that PXT3003 improved neuroelectrophysiological parameters in the CMT1A transgenic rat models; no significant drug-drug interactions were observed with the combination of the three drugs in an in vitro model.

The FDA granted PXT3003 as an orphan drug in 2014 and approved a fast track designation for the treatment of CMT1A in 2019. Due to the good safety and efficacy of the three drugs, PXT3003 is exempted from the phase I clinical trial, and the multi-center phase II clinical study in France and the multi-center phase III clinical study in Europe and the United States have been completed.

The results of phase II clinical study suggested that some efficacy endpoints in patients with mild to moderate CMT1A in the dose (high dose in phase 3II trial) group were improved compared with the placebo group; PXT3003 was safe [6], and 33.8% patients reported adverse reactions possibly or probably related to the investigational drug, most of which were mild. The results of phase III clinical study suggested that patients with mild to moderate CMT1A can benefit from dose group 4 (2 times the high dose in the phase II study) [7], and PXT3003 shows a good safety profile. Adverse reactions include gastrointestinal disorders (nausea, abdominal pain, diarrhea), nervous system disorders (dizziness, headache, and lethargy), musculoskeletal and connective tissue disorders (muscle spasms, myalgia, back pain, neck pain), and systemic symptoms (fatigue, weakness).

In phase III clinical study, due to the crystallization of drug products for dose group 4, a large number of dropouts occurred in dose group 4, and there was a small number of patients included in the per protocol set. Therefore, the FDA requested to carry out another phase III trial to fully verify the efficacy. In order to accelerate the marketing of PXT3003 in China and meet the treatment needs of CMT1A patients in China, the Company plans to independently carry out a multi-center, randomized, double-blind, placebo-controlled phase III clinical study of PXT3003 in CMT1A patients in China.

2. Information on Investigational Drug [8]

(1) Generic Name: PXT3003 oral solution

(2) Chemical Name: (RS)-4-Amino-3-(4-chlorophenyl)butanoic acid, 17-(cyclopropylmethyl)-4,5α-epoxy-3,14-dihydroxymorphin-6-one hydrochloride, (2S,3R,4R,5R)-hexane-1,2,3,4,5,6-Hexol

(3) Molecular Formula: (RS)-baclofen: $C_{10}H_{12}ClNO_2$; naltrexone hydrochloride: $C_{20}H_{23}NO_4 \cdot HCl$; D-sorbitol: $C_6H_{14}O_6$

(4) Molecular Weight: (RS)-baclofen: 213.661 g/mol; naltrexone hydrochloride: 377.86 g/mol; D-sorbitol: 182.17 g/mol

(5) Chemical Structural Formula:

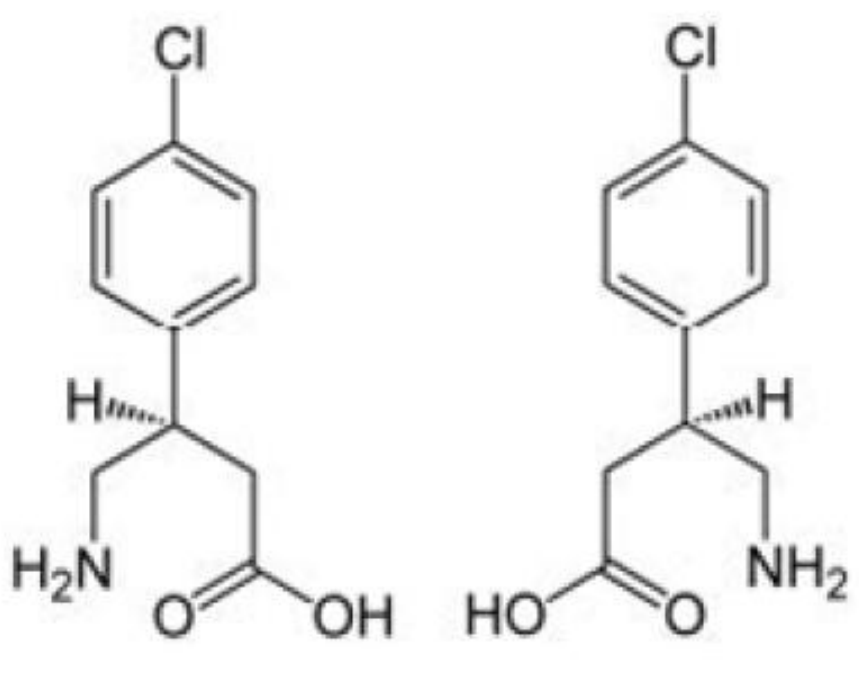

(RS)-baclofen

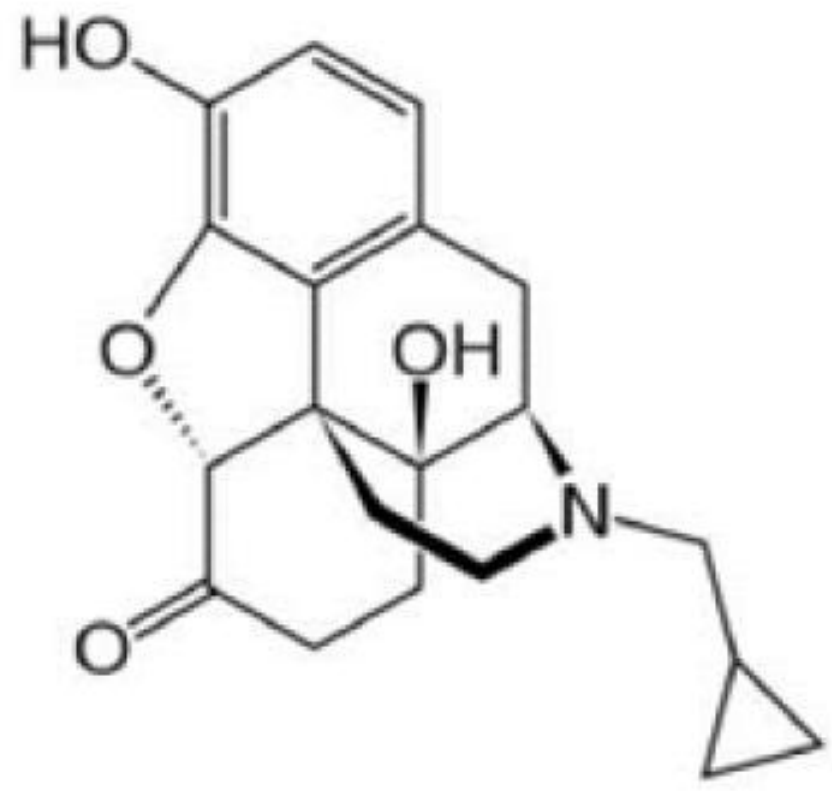

Naltrexone hydrochloride

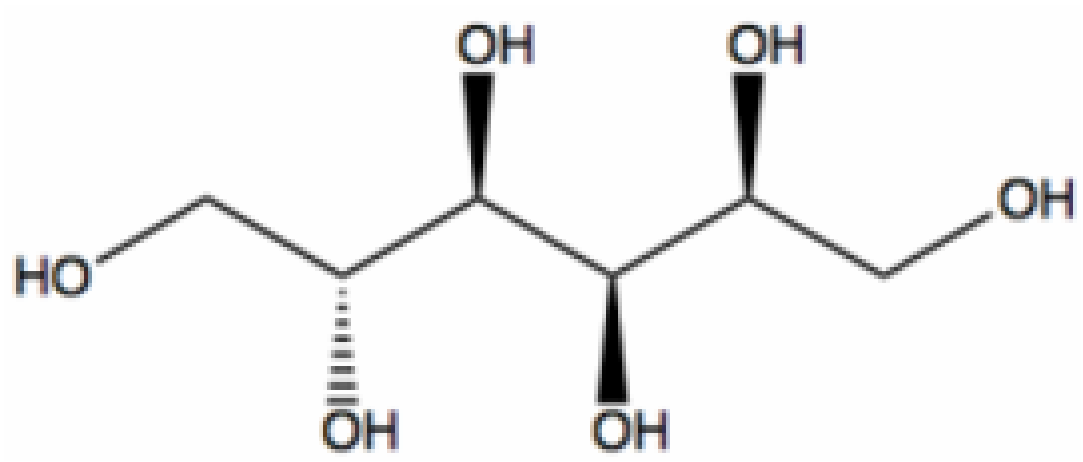

D-sorbitol

(6) Dosage Form and Strength: Oral solution, 100 ml/vial.

(7) Formulation Composition: Main ingredients: (RS)-baclofen 60 mg, naltrexone hydrochloride 7 mg, and D-sorbitol 2100 mg; excipients: acetate buffer, sodium methyl paraben, sodium propyl paraben, and 2-isoamyl acetate (banana flavor).

(8) Storage: Stored in brown glass vials at 2°C–8°C.

2.1 Mechanism of Action

(RS)-baclofen, naltrexone hydrochloride and D-sorbitol are approved, well-established generic drugs.

(RS)-baclofen (Lioresal®, Kemstro®) is a specific GABA-B receptor agonist that can be used to treat muscle rigidity (in multiple sclerosis and spinal disorders), especially for relief of flexor spasms and concomitant pain, clonus, and muscular rigor. In glial cells, (RS)-baclofen reversely modulates adenylate cyclase and reduces intracellular levels of cyclic adenosine monophosphate (cAMP) that promotes PMP22 gene transcription. Therefore, cAMP dependently inhibits PMP22 gene transcription.

Naltrexone hydrochloride (Depade®, Revia®, Nalorex®, or Vivitrol®) is a potent opioid receptor antagonist approved for the treatment of opioid and alcohol dependence. Naltrexone hydrochloride competitively binds to opioid receptors, thereby blocking the effects of opioids. Low doses of naltrexone hydrochloride enhance rather than attenuate the opioid signaling pathway and couple with an inhibited type G protein Alpha Subunit (Gαi) to reduce intracellular cAMP levels.

D-sorbitol (D-sorbitol, D-glucitol, E420) is a polyol that occurs naturally in the stone fruits and berries of many trees of the genus Sorbus. It can also be artificially synthesized by glucose, with a sweetness of about half that of sucrose. It is widely used as food additives, nutritive sweeteners or sugar substitutes, especially for diet foods, mints, cough syrups, and sugar-free chewing gum. D-sorbitol has been used as a diuretic. It treats hyperkalemia when used with potassium-lowering resins. It is used as an irrigant for certain surgical procedures and used to treat constipation in Europe (Sorbitol Delalande®). It is also used as an excipient globally. The target of action of D-sorbitol is unknown. However, studies have shown that it can bind to muscarinic acetylcholine G

protein-coupled receptors (GPCRs), which are present in neurons and glial cells and are associated with intracellular cAMP levels.

2.2 Preclinical Study Information

2.2.1 Pharmacodynamic Study

2.2.1.1 Cellular Level Study

The effects of PXT3003 and its ingredients on myelination were evaluated in vitro using two models: Dorsal Root Ganglia (DRG)/Schwann cell co-culture model of PMP22 transgenic (TG) rat embryos and RT4-D6P2T myelinoma cell line model. Myelination can be quantified by the expression of myelin phospholipid basic protein and *PMP22* gene.

Study PHXCMT0029 evaluated the effect of (RS)-baclofen alone, naltrexone hydrochloride and D-sorbitol and their combination (PXT3003) on myelination in vitro in a Dorsal Root Ganglia/Schwann cell co-culture model of PMP22 transgenic rat embryos. Myelination is quantified by the expression of myelin basic protein. After 10–11 days of drug treatment, (RS)-baclofen, naltrexone hydrochloride and D-sorbitol alone all improved myelination in a dose-dependent manner and PXT3003 promoted myelination more strongly than the ingredients alone. A three-dimensional equivalent line graph was obtained by the dose relationship curve of individual ingredients, and the results showed that the three drugs in PXT3003 play a positive synergistic role, indicating that the drug combination has a synergistic effect.

Studies PHXCMT0066, PHXCMT0067–PHXCMT0069 evaluated the effects of (RS)-baclofen, naltrexone hydrochloride, D-sorbitol and their combination (PXT3003) on the expression of PMP22 in RT4-D6P2T myelinoma cell model. The results showed that 100 nM naltrexone hydrochloride and 10 μM D-sorbitol could significantly down-regulate the expression of *PMP22* mRNA, while 100 nM (RS)-baclofen only had a down-regulation effect. However, PXT3003 at the same dose could significantly down-regulate the expression of *PMP22* mRNA and showed a synergistic effect compared with a single drug.

In studies PHXCMT106 and PHXCMT108, the Dorsal Root Ganglia/Schwann cell co-culture model of PMP22 transgenic (TG) rat embryos was used to evaluate the effects of (RS)-baclofen, naltrexone hydrochloride and D-sorbitol alone and in combination on myelination in vitro through myelin basic protein expression and PMP22 gene expression. The results showed that the three compounds did not promote myelination at the highest and lowest doses when used alone for 28 days. The combination of the three compounds had a significant effect on myelination, but the combination of two drugs at the same dose was ineffective.

The above studies showed that PXT3003 could improve the myelination of transgenic rat nerve cells in vitro, and the effect of improving myelination was related in part to the down-regulation of PMP22 expression, which was validated in myelinoma cells. PXT3003 is more potent and synergistic in the in vitro myelination process compared to (RS)-baclofen, naltrexone hydrochloride, D-sorbitol alone, or combinations of two drugs, supporting further validation of the pharmacodynamics of PXT3003 in animal models.

2.2.1.2 *In Vivo* Studies in Transgenic Rats

PMP22 TG Sprague Dawley (SD) rats are animal models of human CMT1A disease, which can carry three transgenic copies of PMP22 gene of wild-type (WT) rats, resulting in 1.6-fold amplification of PMP22 protein in peripheral nerves. Compared with WT rats, TG rats showed pathological findings of dedifferentiated Schwann cells and decreased cellularity of myelinated axons. Closer to CMT1A disease in humans, this model is characterized by partial demyelination, normal nerve length, and mild neuropathy. Therefore, PHARNEXT selected this model for the preclinical *in vivo* study of PXT3003.

In the transgenic rat CMT1A model, the effects of the drugs screened on muscle strength and neural sensitivity were evaluated through four animal behavioral endpoints, including the rotarod test, inclined plate test, grip strength test, and hot plate test. Bar test and grip strength test are complex behavioral competency tests that can be used to assess motor deficits; an inclined plate test can be used to assess motor function in rats affected by peripheral neuropathy; a hot plate test can be used to assess sensory impairment of CMT1A.

PHARNEXT assumes that the reduced amount of each drug ingredient results in fewer adverse reactions, and also enhances the synergistic effect of each ingredient in PXT3003 and the effect of the combination. The dose selection for in vivo studies was based on the approved dose for each ingredient or the effective dose in clinical studies, with dose reductions for each ingredient, and the ratio of (RS)-baclofen, naltrexone, and D-sorbitol in the rat model was 8.6:1:300.

(RS)-baclofen, naltrexone and D-sorbitol all contribute to the effects of PXT3003 in animal models of CMT1A. To demonstrate the role of each ingredient in PXT3003, 2 trials are designed: A dose-response trial to select the appropriate dose (PHXCMT0109); a trial to compare PXT3003 doses and two-drug combinations (BCL/NTX, BCL/SRB, NTX/SRB) (PHXCMT0119). The dose of PXT3003 is determined from four different doses tested in a preliminary study in CMT1A rats (Table 2-1).

Table 2-1 Doses Selected in Range-Finding Study

Ingredients	Dose of PXT3003 in Animal Study			
	Dose 1	Dose 2	Dose 3	Dose 4
(RS)-baclofen	15μg/kg/day	30μg/kg/day	60μg/kg/day	180μg/kg/day
Naltrexone hydrochloride	1.75μg/kg/day	3.5μg/kg/day	7μg/kg/day	21μg/kg/day
D-sorbitol	0.525μg/kg/day	1.05μg/kg/day	2.1μg/kg/day	6.3μg/kg/day

In the first dose-finding study, 4-week-old CMT1A female rats were stratified by date of birth, source, inclined plate, and grip strength test results (20 animals per dose group). PXT3003 was administered by gavage for 3 months from Week 6 (baseline) (Figure 2-1). Motor behavior was tested by grip strength and bar tests, and the sensory response was tested by hot plate tests. There were no significant differences in phenotype, electrophysiology, or histology between CMT1A females and males. Since the investigational drug in the previous study produced the same therapeutic effect in female and male CMT1A rats, a single-sex model in the preclinical study in CMT1A rats was sufficient for the proof-of-concept for the efficacy of individual drugs or the combination.

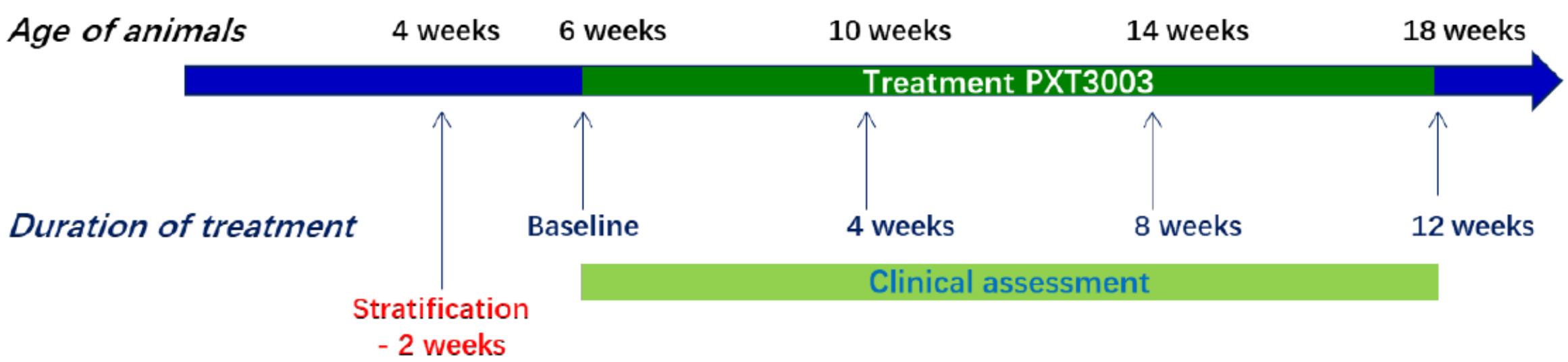

Figure 2-1 Flow Chart for PXT3003 Treatment in CMT1A Rats

After 3 months of treatment, PXT3003 improved motor function in CMT1A rats in the grip strength and bar tests (Figure 2-2). PXT3003 showed significant treatment effects at the end of the study (Week 12). PXT3003 Dose 3 ((RS)-baclofen 60 μg/kg/d, naltrexone hydrochloride 7 μg/kg/d, D-sorbitol 2100 μg/kg/d) is considered to be effective for the treatment of motor deficits.

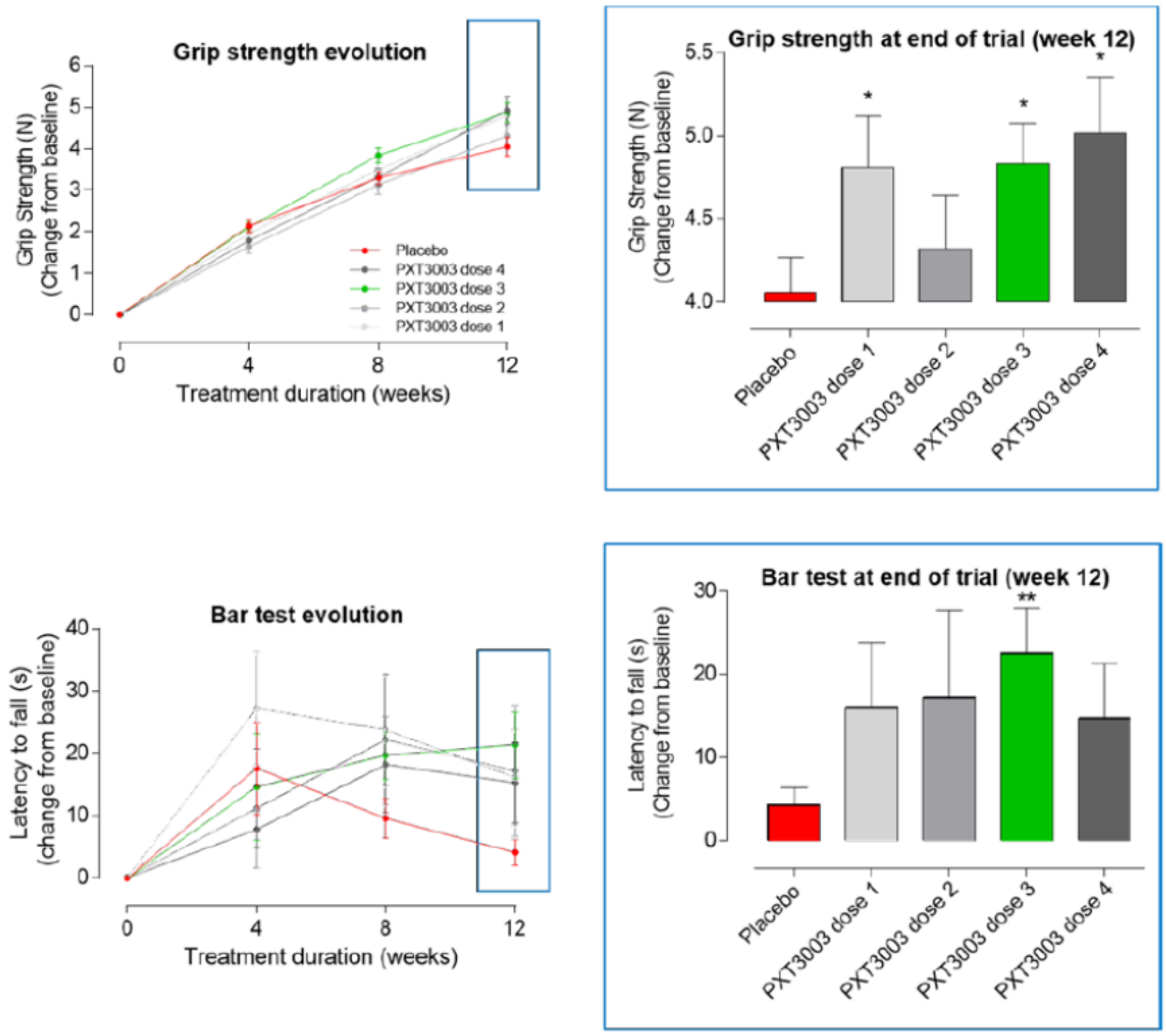

Figure 2-2 Dose-Effect Relationship of PXT3003 in Motor Behavior of CMT1A Rats

(**p* < 0.05, ***p* < 0.001 vs. placebo, t-test)

The hot plate test may reflect a sensory change in CMT1A. PXT3003 changed the sensory loss in a dose-dependent manner, with Dose 3 being the most effective (Figure 2-3). To justify Dose 3 as the effective dose, a comparison of z-scores for motor and sensory endpoints revealed that Dose 3 was the appropriate effective dose (Figure 2-4).

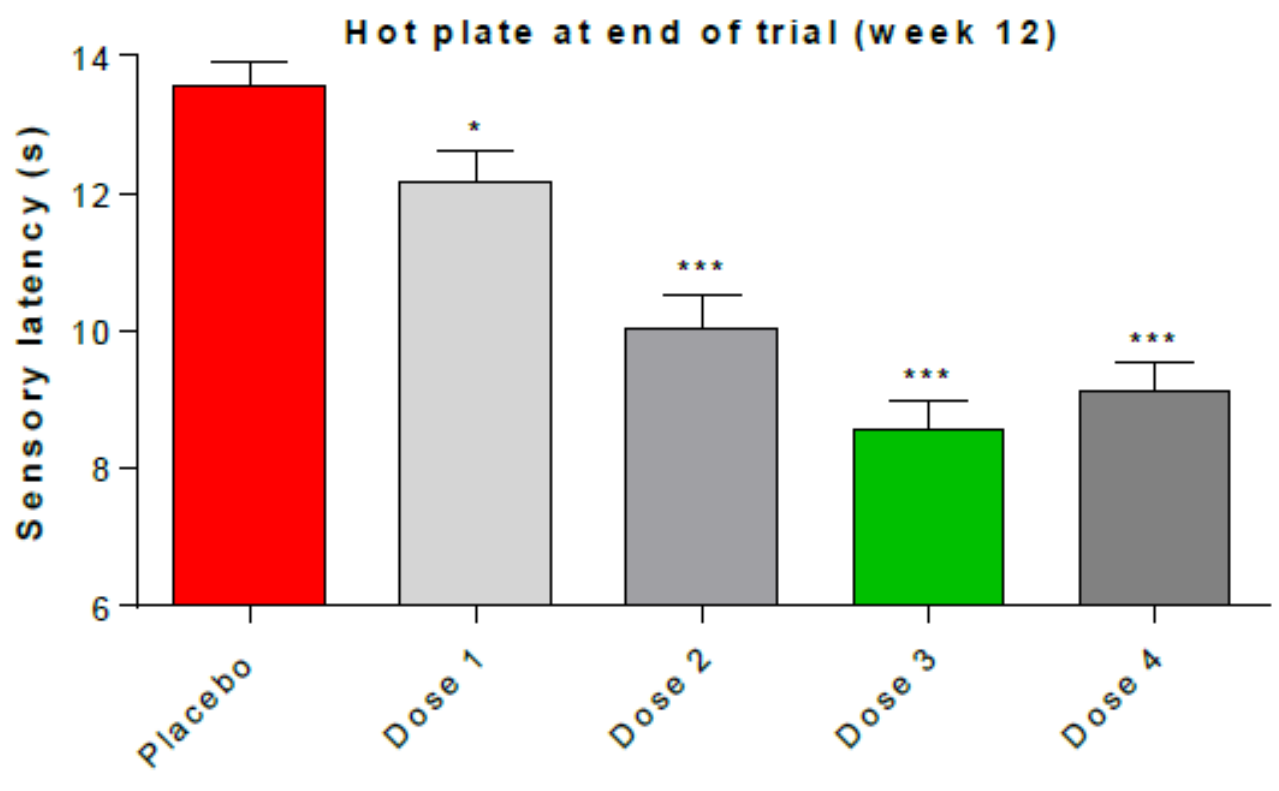

Figure 2-3 Efficacy of PXT3003 to Improve Sensory Behavior in CMT1A Rats

(**p* < 0.05, ***p* < 0.001 vs. placebo, t-test)

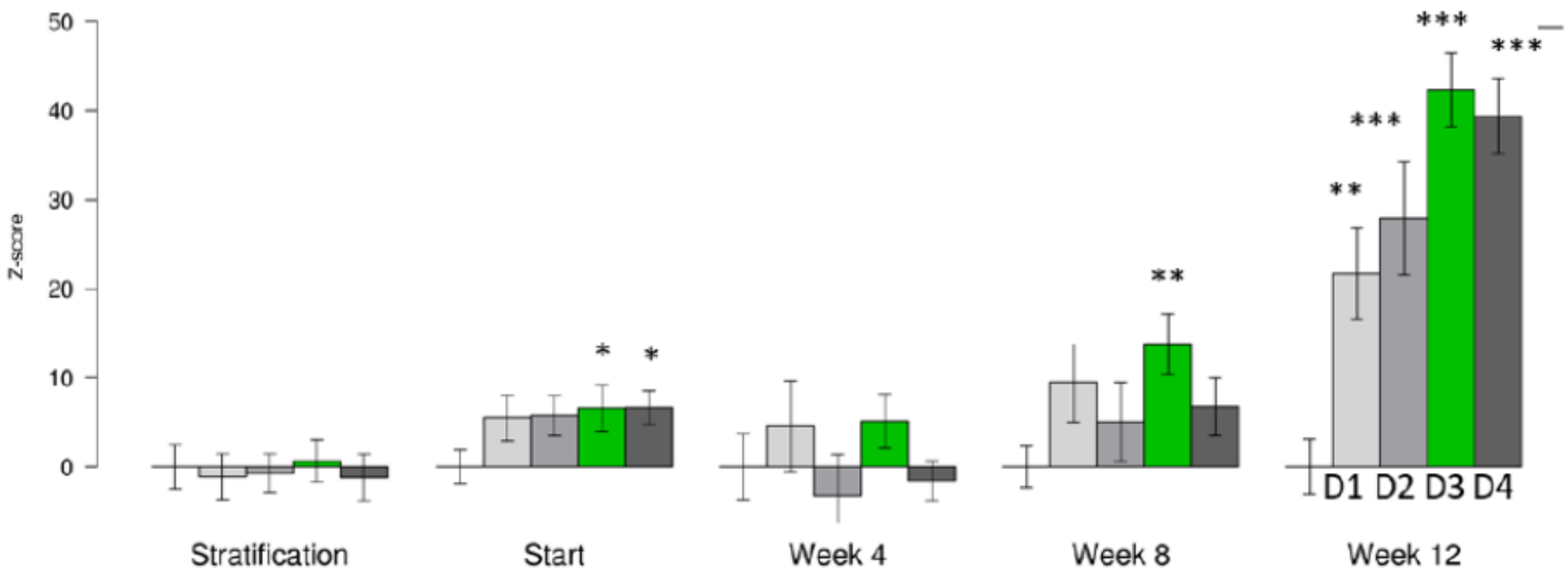

Figure 2-4 Efficacy of PXT3003 Over Time in CMT1A Rats

(**p* < 0.05, ***p* < 0.001, ****p* < 0.001 vs. placebo, t-test)

The motor and sensory endpoints were then compared between PXT3003 dose 3 and two-drug combinations (Table 2-2).

Table 2-2 Comparison of PXT3003 with Two-drug combinations

Grouping	Number of Cases	BCL (μg/Kg/day)	NTX (μg/Kg/day)	SRB (μg/Kg/day)
CMT1A rats + Placebo	20	-	-	-
CMT1A rats + PXT3003	20	60	7	2.1
CMT1A rats + BCL/NTX	20	60	7	
CMT1A rats + BCL/SRB	20	60	-	2.1
CMT1A rat + NTX/SRB	20		7	2.1

PXT3003 could significantly improve the motor function of CMT1A rats in grip strength and bar tests, but no significant effect was achieved with two-drug combinations (Figure 2-5). The left panel shows the effect trend of each treatment group in grip strength and bar tests, and the right panel shows the results of grip strength and bar tests at 12 weeks. PXT3003 had significantly better efficacy than two-drug combinations.

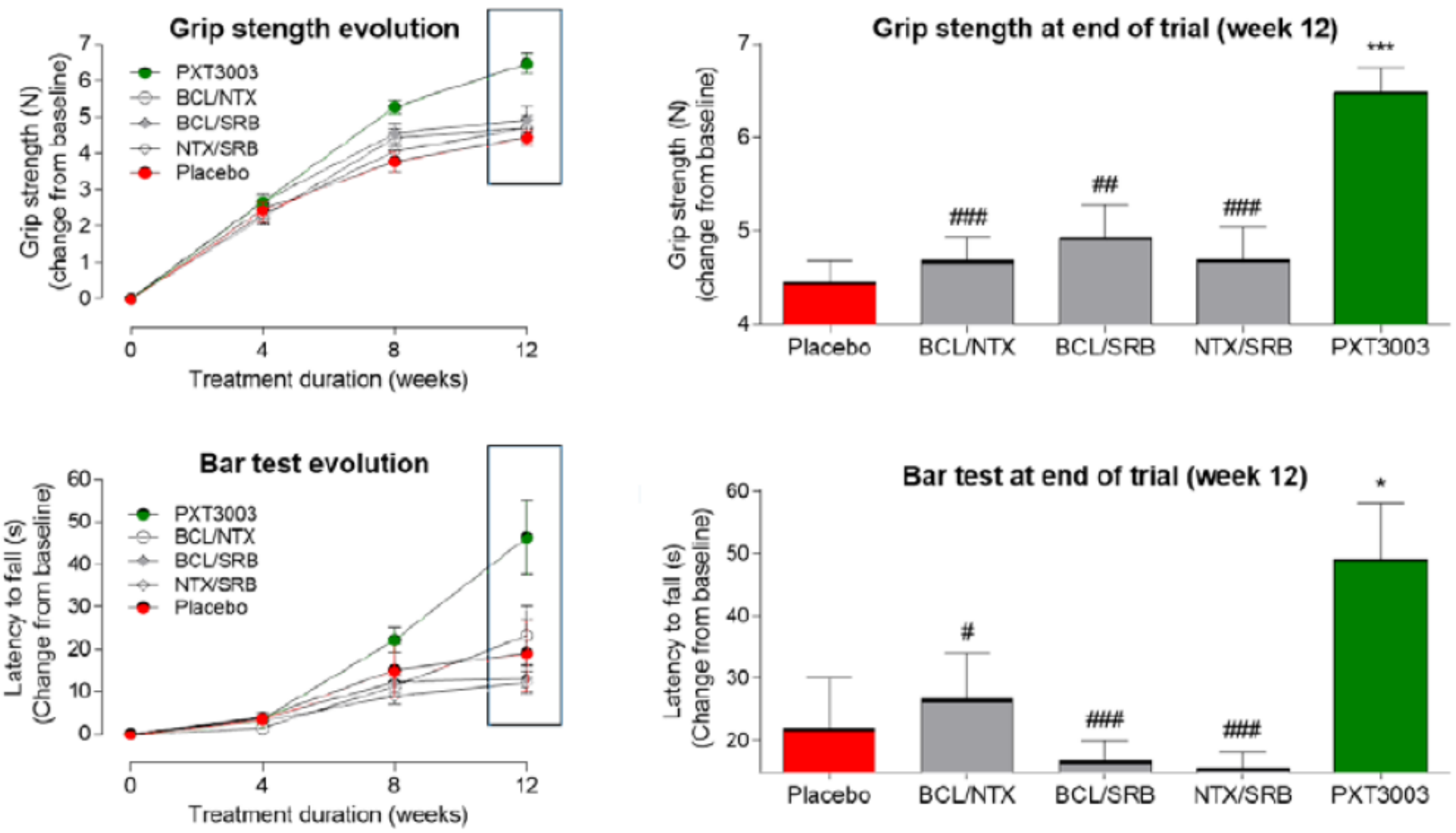

Figure 2-5 Effect of PXT3003 and Two-drug Combinations on Motor Behavior in CMT1A Rats

(*$p < 0.05$, **$p < 0.001$, ***$p < 0.001$ vs. PXT3003, t-test)

The hot plate test is used to evaluate the effect of PXT3003 and two-drug combinations. BCL/NTX significantly improved reaction time, but significantly inferior to PXT3003. The other two-drug combinations did not show statistical differences (Figure 2-6). To further demonstrate the superior efficacy of PXT3003, z-scores for motor and sensory endpoints were assessed and the results supported the superiority of PXT3003 over two-drug combinations (Figure 2-7). PXT3003 significantly and substantially corrected the sensory deficit of CMT1A rats.

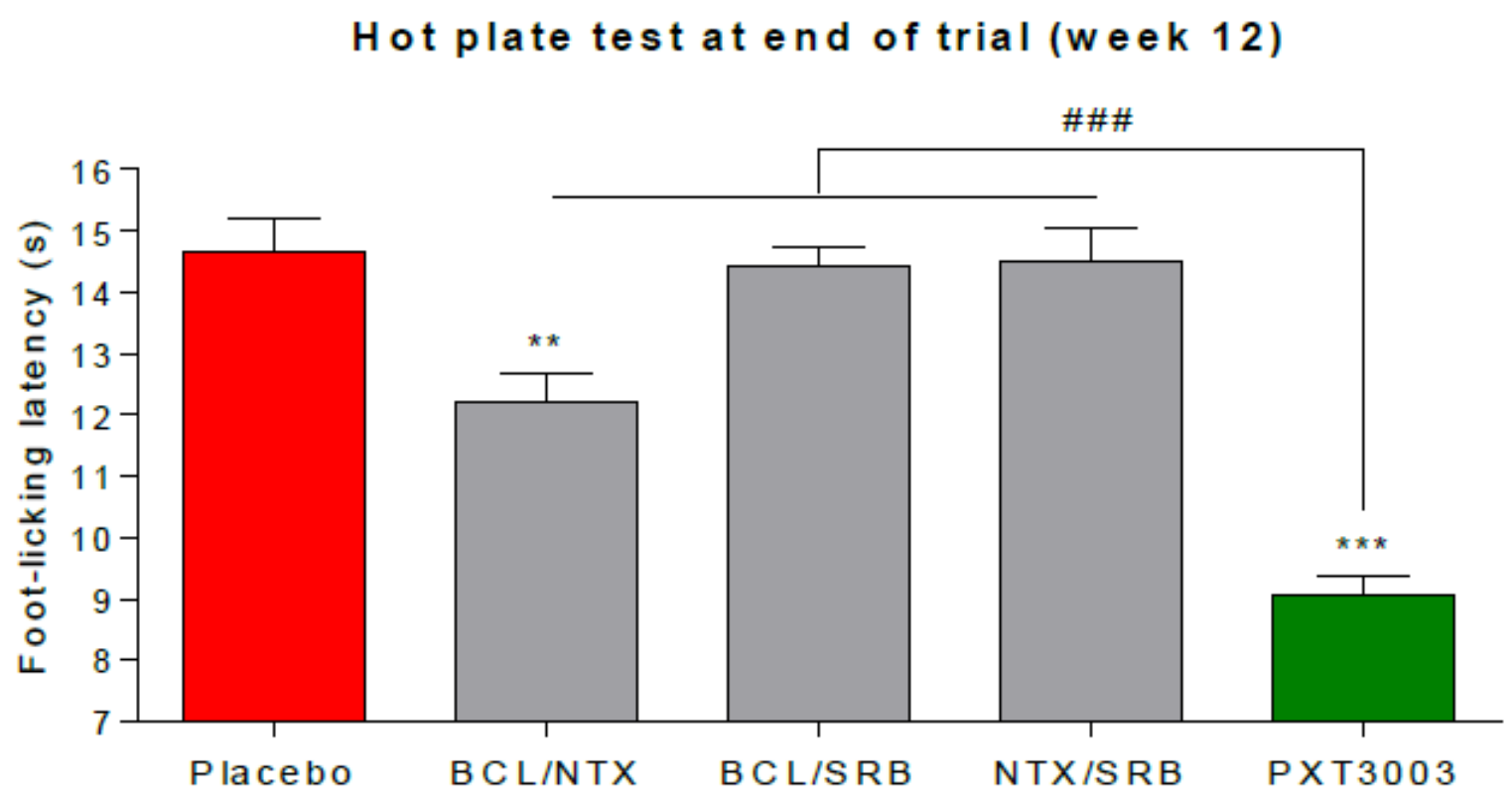

Figure 2-6 Effect of PXT3003 and Two-drug Combinations on Sensory Behavior in CMT1A Rats

(*$p < 0.01$, **$p < 0.001$ vs. placebo, ###$p < 0.001$ vs. PXT3003, t-test)

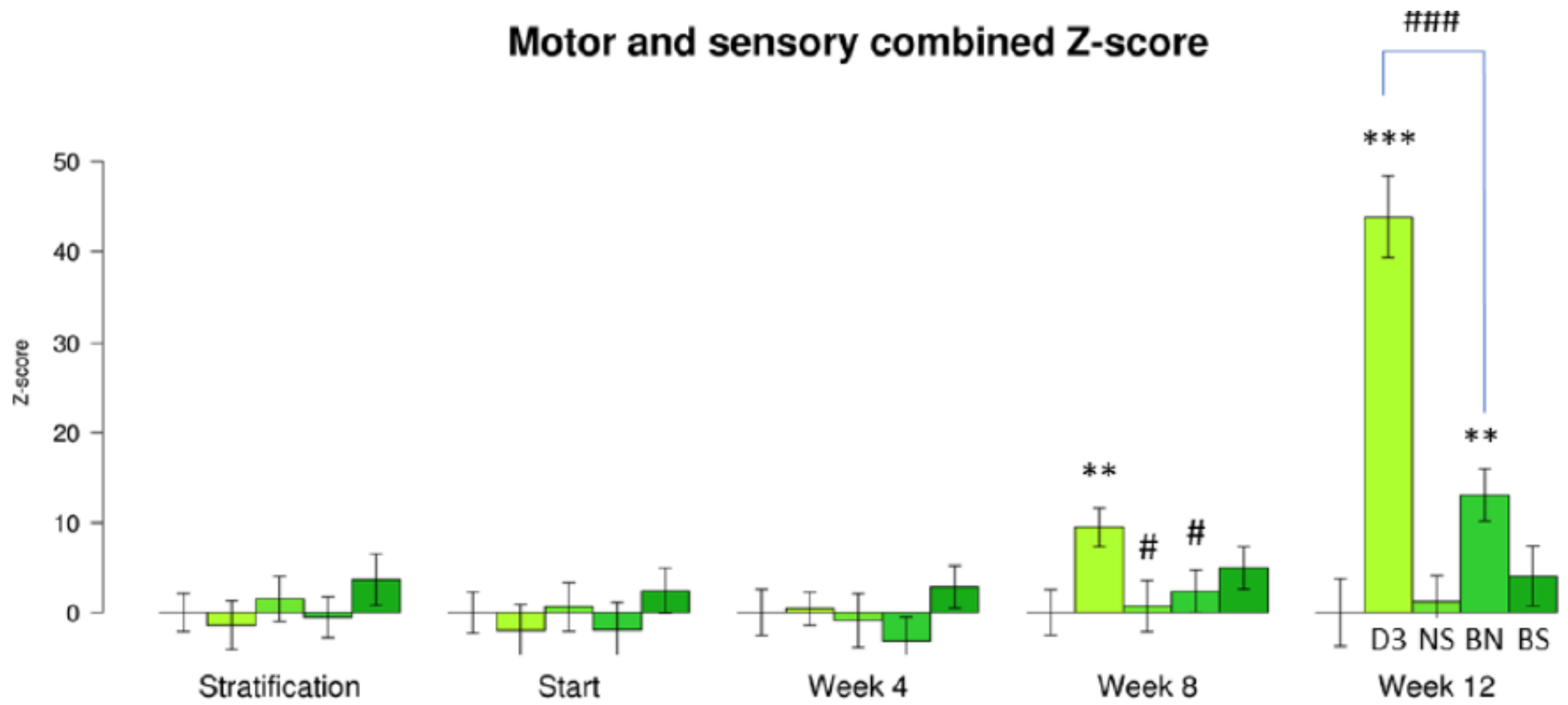

Figure 2-7 Effect of PXT3003 and Two-drug Combinations in CMT1A Rats

(*$p < 0.01$, **$p < 0.001$ vs. placebo, ###$p < 0.001$ vs. PXT3003, t-test)

2.2.1.3 *In Vivo* Study in Mice (Sciatic Nerve Injury Model)

The mouse sciatic nerve injury model shares similar characteristics to inherited demyelinating neuropathy, such as axonal degeneration and glial dedifferentiation. This model can be used to evaluate the ability of early treatment with PXT3003 to rapidly restore peripheral nerve function and myelination. Studies PHXNVT0002 and PHXNVT0004 evaluated the effect of long-term treatment of PXT3003 and its ingredients on sciatic nerve crush injury. Three doses of PXT3003 (Dose 1: 60 μg/kg (RS)-baclofen, 7 μg/kg naltrexone hydrochloride, and 2.1 mg/kg D-sorbitol;

Dose 2: 120 μg/kg (RS)-baclofen, 14 μg/kg naltrexone hydrochloride, and 4.2 mg/kg D-sorbitol; Dose 3: 600 μg/kg (RS)-baclofen, 70 μg/kg naltrexone hydrochloride, and 21 mg/kg D-sorbitol) were studied with this model by oral administration once daily for 42 days to observe the improvement in nerve crush injury. The results showed that PXT3003 Dose 1 significantly improved the compound muscle action potential (CMAP) after 21 and 30 days of dosing, and the axon morphology, myelinated nerve fiber distribution, myelin thickness and axon diameter of regenerative nerve were improved after 42 days of dosing. Neuroethology showed that PXT3003 Dose 3 restored the surface weight bearing of the limbs to normal levels in mice, while the single drug had no significant effect on this.

In this model, PXT3003 demonstrated the potential for nerve regeneration and myelination promotion. The daily dosing of PXT3003 to mice significantly improved CAMP. Axon formation, myelin fiber distribution, and g-ratio distribution of regenerated nerves were significantly improved. The above results suggested that oral PXT3003 could improve the neurophysiology of sciatic nerve injury, axonal morphology, myelin integrity, and functional behavior in mice, while a single drug had no significant effect.

2.2.2 Safety Pharmacology Study

No drug-related serious adverse reactions affecting the cardiovascular system, respiratory system, and nervous system have been found in Phase II and Phase III clinical studies of PXT3003.

The safety pharmacology of PXT3003 has not been evaluated in preclinical studies and the current data are based on a summary of safety pharmacology studies of single drugs.

2.2.2.1 (RS)-baclofen

(RS)-baclofen has depressive and sedative effects on the central nervous system, leading to somnolence, ataxia, and depression of the respiratory and cardiovascular systems. In terms of behavioral effects, maximal effects (approximately 2 hours post-dose) were achieved when rats were dosed with 10 mg/kg (RS)-baclofen, including abnormal posture/gait, stereotypical movements, altered alertness (e.g., hypoactivity, and decreased auditory startle response), decreased muscle tone, significant reductions in tentative autonomic activity, and significant decreases in body temperature. In terms of the respiratory system, Wistar rats had prolonged inspiration and reduced expiratory time after IV (RS)-baclofen. In terms of the cardiovascular system, low-dose (RS)-baclofen ($< 5 \times 10^{-8}$ mol) IV resulted in transient decreases in blood pressure and heart rate in anesthetized rats, while high-dose IV ($> 5 \times 10^{-7}$ mol) resulted in significant increases in blood pressure.

2.2.2.2 Naltrexone hydrochloride

In primates and humans, opioid receptor antagonists have no significant effects on the cardiovascular system (e.g., heart rate and blood pressure). Intravenous administration of naltrexone hydrochloride (5–80 μg/kg) to dogs caused bradycardia at all dose levels but did not affect other parameters (blood pressure, ECG, and blood gas analysis).

2.2.2.3 D-sorbitol

D-sorbitol has been used as a food additive for many years, and several safety pharmacology studies have reported that D-sorbitol has not been found to have any significant effect on physiological function in different species of animals under different routes of administration. D-sorbitol may be used as a non-irritant laxative and may be administered as an oral suspension or enema preparation. The mechanism of action is the absorption of water into the large intestine to stimulate intestinal peristalsis. There is no documented evidence of harmful effects on vital signs with D-sorbitol.

2.2.3 Toxicology Study

Study TOX-PXT3003-01 was a repeat-dose toxicity study of PXT3003 administered by gavage every 12 hours for 7 consecutive days in rats. The trial is divided into 2 phases.

In the stage 1, 4 treatment groups (12 animals per group, half male and half female, approximately 7 weeks of age) were administered orally at dose volumes of 2.5, 5 or 10 ml/kg/time once daily (QD) or twice daily (BID). Single doses of PXT3003 ranged from 0.03 to 171 mg/kg (RS)-baclofen, 0.0035 to 20 mg/kg naltrexone hydrochloride, and 1.05 to 6000 mg/kg D-sorbitol, with (RS)-baclofen, naltrexone hydrochloride and D-sorbitol in a dose ratio of 8.5:1:300. The dose level of PXT3003 is expressed only as the dose level of (RS)-baclofen (mg (RS)-baclofen/kg/time). The results showed that the Maximal Tolerated Dose (MTD) of PXT3003 was not less than 0/85.5, 129/64.3 and 171/42.8 mg (RS)-baclofen/kg/time in male rats, and not less than 129 mg and 171 mg (RS)-baclofen/kg/time in female rats.

In the stage 2, PXT3003 was administered to rats by gavage at 0.03 mg, 3 mg, 10 mg, 30 mg (RS)-baclofen/kg/time (twice daily, 12 hours apart) for 7 consecutive days at a dose volume of 5 ml/kg in 4 treatment groups.

Conclusion: The NOAEL of PXT3003 in male and female rats was 3 mg (RS)-baclofen/0.35 mg naltrexone hydrochloride/105 g D-sorbitol/kg/time, BID.

2.2.4 Pharmacokinetic Study

2.2.4.1 Single-dose Pharmacokinetic (PK) Study

Study PKA-PXT3003-04 evaluated the pharmacokinetics of single intravenous and oral doses of PTX3003 in male SD rats After intravenous and intragastric administration of 60 μg/kg (RS)-baclofen, 7.0 μg/kg naltrexone hydrochloride and 2.1 mg/kg D-sorbitol in rats, naltrexone hydrochloride was only detected in plasma samples at 2 and 10 min after intravenous administration, while the drug concentrations in blood samples at other time points and all blood samples after intragastric administration were all below quantifiable limit. In addition, D-sorbitol was tested similarly to naltrexone hydrochloride and therefore no pharmacokinetic parameters were derived for either compound. The main pharmacokinetic parameters of (RS)-baclofen in PXT3003 following intravenous and oral administration are shown below:

Table 1 Main PK Parameters of (RS)-Baclofen in PXT3003 After Intravenous Administration

C_0 (ng/mL)	ke (h-1)	$t_{1/2}$ (h)	AUC_{0-t} h.ng/mL)	AUC_{0-inf} (h.ng/mL)	$\%AUC_{extra}$	Vd (mL/kg)	Vd (L)	Cl (mL/h/kg)	Cl (L/h)
230	0.329	2.11	138	143	3.5	1279	4.2	420	1.38

Table 2 Main PK Parameters of (RS)-Baclofen After Gavage Administration of PXT3003

T_{max} (h)	C_{max} (ng/mL)	F (%)	AUC_{0-t} (h.ng/mL)	AUC_{0-inf} (h.ng/mL)	$\%AUC_{extra}$
0.99	39.8	110	137	158	12.9

Study PKA-PXT3003-05 was a single oral dose pharmacokinetic study of (RS)-baclofen, naltrexone hydrochloride, D-sorbitol alone and PTX3003 in male SD rats. The dose of PXT3003 is 3 mg/kg (RS)-baclofen, 350 μg/kg naltrexone hydrochloride, and 105 mg/kg D-sorbitol. The dose of the three drugs administered alone is the same as that in PXT3003. Plasma concentrations of naltrexone hydrochloride following oral administration were below quantifiable limit (0.500 ng/ml) and no pharmacokinetic parameters were established. The measured concentrations of D-sorbitol ranged from 150 to 650 ng/ml, which was similar to the endogenous level, and no pharmacokinetic parameters were established.

The results for (RS)-baclofen alone and in combination (PXT3003) are presented in Table 3 and Table 4:

Table 3 (RS)-baclofen (3 mg/kg) Orally Alone

T_{max} (h)	C_{max} (ng/mL)	F (%)	AUC_{0-t} (h.ng/mL)	AUC_{0-inf} (h.ng/mL)	$\%AUC_{extra}$
1.00	1146	69.2	4835	4846	0.2

Table 4 (RS)-baclofen (3 mg/kg) Administered Orally as Combination PXT3003

T_{max} (h)	C_{max} (ng/mL)	F (%)	AUC_{0-t} (h.ng/mL)	AUC_{0-inf} (h.ng/mL)	$\%AUC_{extra}$
1.03	1610	85.1	5844	5864	0.3

Conclusion: Compared with 3 mg/kg in PXT3003, (RS)-baclofen 3 mg/kg alone has similar T_{max}, while C_{max}, AUC_{0-inf} and F are lower, so (RS)-baclofen is more exposed in the combination (PXT3003).

2.2.4.2 Tissue Distribution

The results of the human plasma protein binding study of (RS)-baclofen and naltrexone hydrochloride alone and in combination with D-sorbitol (PXT3003) suggested that the binding of (RS)-baclofen and naltrexone hydrochloride to human plasma proteins was not significant at the five preset plasma concentration levels. There was no significant difference in plasma protein binding results between individual compounds and PXT3003 incubated in plasma, and it was inferred that the drug was present in the human body as free. A pharmacokinetic study of PXT3003 following single intravenous and oral administration in male SD rats (PKA-PXT3003-04) showed that the apparent volume of distribution (Vd) of (RS)-baclofen (60 μg/kg) was 4.2 L after intravenous administration of PXT3003 to rats. It is speculated that it has a wide range of tissue distribution.

The literature on the tissue distribution of (RS)-baclofen, naltrexone hydrochloride and D-sorbitol used alone is summarized below:

(1) (RS)-baclofen: The plasma protein binding rate of (RS)-baclofen is about 30%. It is mainly distributed in liver and kidney tissues and can pass through the blood-brain barrier and placental barrier. It is eliminated at a slower rate in the brain than in blood and other tissues.

(2) Naltrexone hydrochloride: After intravenous injection of naltrexone hydrochloride, it is mainly distributed in the brain, lung, spleen, liver, kidney, muscle, testis, fat, stomach, and small intestine, with a plasma protein binding rate of about 20–26%.

(3) D-Sorbitol: A study investigating the concentrations of D-sorbitol in serum, liver and lens of rats orally administered with ^{14}C-labelled D-sorbitol found that D-sorbitol was more distributed in the eyeball, followed by liver and less distributed in serum after administration of 20.4 μCi [^{14}C] 100 mg in rats.

2.3.4.3 Metabolism

(1) *In Vitro* Metabolism Study of PXT3003

Study PKA-PXT3003-07 analyzed species differences in the metabolism of PXT3003 in SD rat, Beagle dog and human hepatocytes incubated with (RS)-baclofen, naltrexone hydrochloride, and D-sorbitol at concentrations of 8.57 μM, 1 μM, and 300 μM, respectively, without cytotoxicity. The study results showed that (RS)-baclofen did not metabolize significantly in the above three hepatocyte reaction systems, while naltrexone hydrochloride was completely metabolized after incubation for 24 h, but its metabolite 6β-naltrexol was only detected in the human hepatocyte reaction system.

Study PKA-PXT3003-01 evaluated the metabolic profiles of (RS)-baclofen, naltrexone hydrochloride, and PXT3003 by using human liver microsomes and S9 fractions, and the results

showed that (RS)-baclofen did not metabolize in both liver microsomes and S9 fractions. Naltrexone hydrochloride did not metabolize in liver microsomes, but it was metabolized to 6-β-naltrexol by S9 fractions. In addition, there was no significant difference in drug metabolism results between naltrexone hydrochloride incubated alone and incubated in the combination (PXT3003), indicating that there was unlikely to have drug-drug interaction among the 3 drugs in PXT3003.

(2) Drug metabolism of (RS)-baclofen, naltrexone hydrochloride and D-sorbitol alone

(RS)-baclofen: It mainly produces inactive hydroxylated metabolites through deamination and oxidation in the liver. Naltrexone hydrochloride: Studies have shown that after administration of naltrexone hydrochloride to animals and humans, it is mainly excreted in the urine, and its reduced product 6β-naltrexol is its main metabolite, with methylation and dealkylation as its secondary metabolic forms. D-sorbitol: Studies have shown that D-sorbitol can be rapidly transformed into sugar, glucose, and other compounds through the liver in rats.

2.3.4.4 Excretion

After oral administration of (RS)-baclofen, most of the drug (90%) is excreted in the urine in the unchanged form, with a terminal elimination half-life of 1.5–6.0 h (about 5.5 h in the human body). If the dose is too high, the terminal elimination half-life can be prolonged. It has been reported in the literature that naltrexone hydrochloride is mainly excreted in the urine (42%) and feces (55%) as the unchanged drug and metabolite 6β-naltrexol. D-sorbitol was excreted by glomerular filtration in animals and humans, and the renal clearance in dogs and humans was 74–77 ml/min and 83–136 ml/min, respectively.

2.3.4.5 Effects on Metabolic Enzymes

Study PKA-PXT3000-02 investigated the inhibition of cytochrome P450 by (RS)-baclofen and naltrexone hydrochloride *in vitro* by using human hepatocyte microsomes[22]. (RS)-baclofen (range: 0.1–10 μM) did not inhibit the activity of CYP1A2, CYP2A6, CYP2B6, CYP2C8, CYP2C9, CYP2C19, CYP2D6, CYP2E1 and CYP3A4/5 (substrates were nifedipine, midazolam, and testosterone). Naltrexone hydrochloride (range: 0.1–10 μM) did not inhibit CYP1A2, CYP2A6, CYP2B6, CYP2C8, CYP2C9, CYP2C19, CYP2E1, and CYP3A4/5 (substrates were nifedipine, midazolam, and testosterone). Naltrexone hydrochloride only had a slight inhibitory effect on CYP2D6 above therapeutic concentrations (10 and 20 μM).

Study PKA-PXT3003-03 evaluated the induction of cytochrome P450 enzymes (CYP1A2, CYP2B6, and CYP3A4) expressed by HepaRG® by (RS)-baclofen and naltrexone hydrochloride. (RS)-baclofen incubated alone (range: 0.1–10 μM) slightly reduced the activity of CYP1A2 and CYP3A4 but did not show a dose-response relationship; slight induction of CYP2B6 activity (21% of control compound inducers) was also observed without a clear dose-response relationship, which was below the FDA-recommended standard (40% of positive control). Naltrexone hydrochloride incubated alone (range: 0.1–10 μM) reduced CYP2B6 activity (dose-related) and CYP3A4 activity (dose-unrelated) and slightly decreased CYP1A2 activity (apparently dose-related).

3. Study Objectives

Primary objective: To assess the efficacy of PXT3003 on Charcot-Marie-Tooth Disease type 1A (CMT1A) measured by the Overall Neuropathy Limitation Scale (ONLS) scores in patients treated for 15 months.

Secondary objective: To assess the efficacy of PXT3003 on functional tests and quality of life measures, and assess the safety of PXT3003.

4. Trial Design

4.1 Overall Design

A randomized, double-blind, placebo-controlled, and multicenter study

4.2 Justification of Trial Design

The study design is based on the *Good Clinical Practice* [9], the *Provisions for Drug Registration* [10], and the *Requirements for Registration Classification and Application Data of Chemical Drugs* [11].

4.3 Randomization

The Interactive Web Response System (IWRS) will be used in this study, and subjects who participate in the screening of this study will be assigned a screening number. After the screening, the investigator will log in to the electronic data capture system to fill in the screening data, and subjects who meet the enrollment criteria will obtain a random number and corresponding drug number from the IWRS. Subjects will receive system-specified medications for the study.

4.4 Blinding

4.4.1 Blinding and Blind Code Preservation

The subject randomization schedule and drug randomization schedule will be generated in SAS software by professionals who are not involved in the clinical operation and statistical analysis of this study. During drug blinding, the blind coding personnel should prepare a blinding record and keep it as one of the documents of the clinical trial. The schedule includes drug preparation, drug packaging, usage, storage requirements, and blinded quantity.

Blind codes and corresponding SAS programs will be stored at designated locations on the server of the statistical analysis unit and will be only available to the unblinded statistician. Upon completion of drug blinding, blind codes will be sent to the central randomization system unit by encrypted mail and imported into the IWRS for the assignment of random numbers and dispensing of drugs.

4.4.2 Provisions for Unblinding

At the request of the Sponsor or its client, after all study data are entered and the database is locked, blind codes will be exported from the IWRS by the central randomization system unit and then used for analysis after quality control (QC) by the unblinded statistician.

4.4.3 Provisions for Unblinding Individual Cases in Emergencies

The investigational drug should not be unblinded by the investigator unless information concerning the study drug is necessary for the medical treatment of the subject. When a subject experiences a medical emergency that requires immediate identification of the type of drug taken, whenever possible, the Principal Investigator and relevant personnel of the Sponsor must be notified prior to unblinding of the investigational drug. After obtaining approval from the Principal Investigator, the IWRS system can be accessed for emergency unblinding to obtain the specific grouping information of the subject. In case of failure to contact the Sponsor prior to unblinding, contact must be made with the Sponsor within 24 h after unblinding, and the investigator should record the time and reason for unblinding in detail in the eCRF (the unblinded treatment number should not be recorded in the eCRF). Subjects who are emergently unblinded are treated as dropouts.

4.5 Determination of Sample Size

The randomization ratio for this study is 1:1. Assuming that the difference in the improvement in ONLS scores from baseline between the investigational drug group and the placebo group is 0.3 and the standard deviation is 0.6, type I error is controlled at 0.05 (two-sided), and at least 80% power is given to test the superiority of the investigational drug group over the placebo group, 70 evaluable subjects are required for each group. Based on a 20% dropout rate, 88 subjects are required to be enrolled in each group, and thus the total sample size is 176.

The sample size is calculated on PASS (version 15.0).

4.6 Selection of Control Drug

Since there is currently no approved effective drug for CMT1A at home and abroad, a placebo will be used as the control in this phase III clinical trial.

4.7 Design of Dose Group

4.7.1 Analysis of Preliminary Clinical Trial Results

The first human Phase II clinical study of PXT3003 completed in France (CLN-PXT3003-01, NCT01404257) was a multi-center, randomized, double-blind, placebo-controlled study to evaluate the safety, tolerability and efficacy of PXT3003 in patients with CMT1A treated for 12 months [6]. This study enrolled 80 patients, aged 18–65, with mild to moderate CMT1A confirmed by genetic analysis. Patients were randomized into 4 treatment groups in a 1:1:1:1 ratio, including 21 patients in the PXT3003 low-dose (Dose 1) group, 21 patients in the medium-dose (Dose 2) group, 19 patients in the high-dose (Dose 3) group, and 19 patients in the placebo group. PXT3003 low-dose: (RS)-baclofen 0.6 mg, naltrexone hydrochloride 0.07 mg and D-sorbitol 21 mg, BID; medium-dose: (RS)-baclofen 1.2 mg, naltrexone hydrochloride 0.14 mg and D-sorbitol 42 mg, BID; high-dose: (RS)-baclofen 6 mg, naltrexone hydrochloride 0.7 mg and D-sorbitol 210 mg, BID. Compared with the placebo group, the high-dose group showed improvement in primary endpoints, i.e., Charcot-Marie-Tooth Neuropathy Score (CMTNS) and ONLS.

The pivotal Phase III clinical study completed in Europe and the United States (CLN-PXT3003-02, NCT0257975) was a multi-center, randomized, double-blind, placebo-controlled study to evaluate the efficacy and safety of 2 different doses of PXT3003 compared with placebo in patients with mild to moderate CMT1A treated for 15 months [7]. Due to slow disease progression and in consideration of potential improvements in efficacy parameters, the duration of treatment was set at 15 months. Dose 3 is the high dose in the PXT3003 Phase II study: (RS)-baclofen 6 mg, naltrexone hydrochloride 0.7 mg and D-sorbitol 210 mg; Dose 4 is 2 times the high dose in PXT3003 Phase II: (RS)-baclofen 12 mg, naltrexone hydrochloride 1.4 mg and D-sorbitol 420 mg. 323 subjects were actually enrolled, 109 subjects in Dose 3 group, 113 subjects in Dose 4 group, and 101 subjects in placebo group. Due to crystallization of the investigational drug, the dropout rate of the study was high, and a small number of subjects were finally included in the per-protocol set, including 68 subjects in Dose 3 group, 38 subjects in Dose 4 group, and 60 subjects in placebo group.

The results of phase III clinical study suggested that in terms of the primary endpoint ONLS, the Dose 4 group had a statistically significant effect compared with the placebo group, and the Dose 3 group had no statistically significant effect compared with the placebo group. Secondary endpoints and exploratory endpoints: In terms of the 10-meter walking test and CMTNS-V2 sensory symptoms, the Dose 4 group had statistically significant effects compared with the placebo group; in terms of other endpoints, the Dose 4 group had no statistically significant effect compared with the placebo group, and the Dose 3 group had no statistically significant effect compared with the placebo group.

A "dose-effect relationship" was observed in Phase II/III clinical studies of PXT3003, and the results of Phase III clinical studies showed that Dose 4 was the effective dose. In view of the crystallization at Dose 4 in Phase III clinical study, the investigational drug in this study was twice the Dose 3 in Phase III clinical study completed in Europe and the United States.

4.7.2 Design of Dose Group and Sample Size

The study is divided into two groups: the investigational drug group and the placebo group. The specific grouping and sample size are shown below:

Table 5 Grouping and Sample Size

Group	Dose and Frequency	Course of Treatment	Number of Subjects
Investigational drug group	10 ml BID	15 months	88
Placebo group	10 ml BID	15 months	88

5. Study Population

5.1 Diagnostic Criteria

The diagnosis of CMT1A refers to the following criteria:

(1) One of the following clinical manifestations: ① slowly progressive muscle weakness and atrophy of the distal extremities; ② peripheral sensory disturbance, diminished or absent tendon reflexes; ③ deformity: high arched feet, crane leg deformity, and scoliosis.

(2) Family genetic history: ① clear family history is an important basis for the diagnosis of CMT; ② absence of family history cannot rule out CMT;

(3) Other causes of chronic peripheral neuropathy, such as diabetes, autoimmune diseases or poisoning, are excluded.

(4) Neurophysiological examination: ulnar nerve motor nerve conduction velocity > 15 m/s;

(5) Genetic diagnosis: *PMP22* gene duplication.

5.2 Inclusion Criteria

Subjects must meet all of the following inclusion criteria to be included in the study:

(1) Male or female, aged 16–65 years (inclusive);

(2) Subjects with a proven genetic diagnosis of CMT1A (PMP22 duplication on chromosome 17p11.2);

(3) Subjects with CMTNS-V2 scores >2 and ≤ 18;

(4) Subjects with muscle weakness in at least foot dorsiflexion (clinical assessment);

(5) Subjects with ulnar nerve motor nerve conduction velocity > 15 m/s;

(6) Subjects who provide signed informed consent forms to voluntarily participate in the clinical trial, and are able to understand and comply with all study procedures.

5.3 Exclusion Criteria

Subjects who meet any of the following are not eligible for the trial:

(1) Subjects with known hypersensitivity to (RS)-baclofen, naltrexone hydrochloride, D-sorbitol or PXT3003 excipients, or history of other serious allergies;

(2) Subjects with contraindications to use of baclofen, naltrexone, or sorbitol, such as porphyria;

(3) Subjects with any other associated cause of peripheral neuropathy such as diabetes (including a history of diabetes and glycated hemoglobin > 6.5%);

(4) Subjects with another nervous system disorders that affect the evaluation of study treatment;

(5) Subjects with ONLS score of 0;

(6) Subjects with a clinically significant history of an unstable medical illness (unstable angina pectoris, tumor, hematological diseases, hepatitis or liver failure, renal failure, etc.) in the past 1 year that may jeopardize the participation in the study;

(7) Subjects who received limb surgery within six months before randomization or planned before completion of the clinical trial;

(8) Subjects with hepatic or renal dysfunction:
   a. TBIL>1.5×ULN, ALT>3×ULN, AST>3×ULN;
   b. Cr>1.5×ULN;

(9) Subjects with positive syphilis antibody and HIV antibody;

(10) Subjects with tumors suggested by chest X-ray or B ultrasound;

(11) Subjects with a history of alcohol dependence in the past 3 months;

(12) Pregnant or lactating women, and subjects who cannot use appropriate contraceptive measures during the trial;

(13) Subjects who used concomitant treatment 4 weeks prior to enrollment, including but not limited to baclofen, naltrexone, sorbitol, opioids, vitamin C, levothyroxine, and potentially neurotoxic drugs (e.g., amiodarone, chloroquine);

(14) Subjects unable to complete study follow-up;

(15) Subjects who have participated in another investigational drug trial and used the investigational drug in the past 30 days;

(16) If a subject has already been included in this study, it will not be possible to include another one from the same family and living in the same household to avoid confusion in treatment, and the affection on blind treatment and interpretation of study results;

(17) Subjects who are not suitable for participation in this clinical trial due to poor compliance or other factors as judged by the investigator.

5.4 Dropout/Withdrawal Criteria

(1) Subjects who withdraw from the study of their own accord and withdraw their informed consent forms;

(2) Subjects who cannot tolerate adverse events;

(3) Females who become pregnant during the study;

(4) Other reasons that the investigator considers the subjects are unable to continue the trial;

(5) Subjects with poor compliance (failure to take prescribed medications and receive clinical evaluation; other behaviors that seriously affect the trial results);

(6) Lost to follow-up.

When the subject withdraws, the investigator should fill in the reason for withdrawal in the medical record, complete the assessment items that can be completed, and fill in the last visit record in the medical record. The data of withdrawn subjects should be entered into the Electronic Case Report Form (eCRF). Withdrawals due to adverse events should be recorded in the eCRF and the Sponsor should be informed. Subjects who are successfully screened but withdraw from the study without obtaining the subject number will not be considered as withdrawal subjects.

6. Investigational Drug

6.1 Basic Information of Investigational Drug

Investigational drug: generic name: PXT3003 oral solution, dosage form: oral solution, strength: 100 ml/vial, containing (RS)-baclofen 60 mg, naltrexone hydrochloride 7 mg, and D-sorbitol 2100 mg. Excipients: acetate buffer, sodium methyl paraben, sodium propyl paraben, 2-isoamyl acetate (banana flavor). Storage: packed in brown glass vials, stored at 2°C–8°C.

Placebo: PXT3003 oral solution placebo, strength: 100 ml/vial. Storage: packed in brown glass vials, stored at 2°C–8°C.

Dosage and administration: twice daily, oral with meals (breakfast and dinner), 5 ml/dose in the first 2 weeks, and thereafter 10 ml/dose.

6.2 Package and Label of the Drug

Packaging: The investigational drug and placebo are provided by Genenet Pharmaceutical Co., Ltd. PXT3003 is a transparent liquid preparation, 100 ml of oral solution in a 100 ml pharmaceutical-grade transparent brown glass vial. Each vial has a low-density polyethylene (PE) pipette adaptor at the mouth for taking drug solution with a flat-head tube, and a threaded polypropylene bottle cap with tamper-resistant and child-resistant features. Each vial is individually packed in a single box and equipped with a separate 5 ml polyethylene tube.

The investigational drug vial label is detailed in Appendix 1.

6.3 Storage, Management and Distribution of Investigational Drug

The investigational drug will be uniformly delivered by the Sponsor to the study sites. According to the PXT3003 drug stability report, the shelf life of PXT3003 is 24 months under refrigerated conditions (5°C ± 3°C). Considering transportation, it is allowed to hold at ambient temperature (< 30°C) for a total period of not more than 30 days. Once the PXT3003 vial is opened, it is required to be stored in a refrigerator at 2°C–8°C for up to 10 days.

The study site should sign the drug receipt form and drug warehouse-in/warehouse-out record when receiving the investigational drug. The investigational drug is required to be stored at 2°C–8°C, and the temperature monitor is used to continuously monitor the storage refrigerator of the investigational drug and record the temperature value. The investigational drug should be locked in a special cabinet in the study site by a specially designated person. The investigational drug administrator will distribute the investigational drug to the study nurse/study doctor according to the subject number information, and a specially designated person will be responsible for distributing the drug. The distribution and recovery of each portion of drug should be recorded in a special record form in time.

Remaining drugs, packing boxes and labels will be recovered in a timely manner during and after the study. Drug recovery form should be signed and all the drugs for clinical trials should be properly kept. They should be handed over to the Sponsor after the end of the study or retained as required. Drugs for clinical trials should not be transferred to any non-clinical trial participant or any third party.

The study site is obliged to keep the drug list and accept drug monitoring and audit by the monitor, auditor and drug regulatory authorities. The monitor is responsible for monitoring the supply, use and storage of the clinical investigational drug as well as the disposing process of remaining drugs.

6.4 Medication Compliance

In the informed phase, the investigator will introduce in detail the objectives of the study, the basic information of the investigational drug, the study procedures, the dosing regimen (such as dose, route of administration, and period), the frequency and process of clinical observation and biological sample collection, potential risks and benefits of participation in the study, etc. to ensure that the subjects are fully informed and voluntarily participate in the study, so as to improve the compliance of medication.

6.5 Provisions for Concomitant Medication

Supportive treatments allowed during the trial include painkillers (except opioids), physiotherapy, and orthopedic devices. Any drugs and treatments other than the investigational drug

should be recorded in detail in the case report form from signing the informed consent form to the last medication, including generic name, dose, frequency, start and end dates of medication, and indications.

Concomitant medications such as baclofen, naltrexone, sorbitol, vitamin C, levothyroxine, and potentially neurotoxic drugs such as opioids including morphine, fentanyl, oxycodone, buprenorphine, tramadol, and methadone are prohibited 4 weeks prior to enrollment and throughout the study (see Appendix 2).

7. Trial Procedures

The study is divided into 2 periods: Screening visit/inclusion visit period and trial period, as shown in the study flow chart, and the specific schedule is as follows.

7.1 Screening Visit/Inclusion Visit (D-30 to D-1)

After the informed consent form is signed, the investigator will judge whether the subject meets the inclusion/exclusion criteria according to the examination results during the screening period. The screening visit lasts from D-30 to D-2 and the inclusion visit is conducted on D-1; the screening visit and the inclusion visit can be completed on the same day.

Examinations and knowledge required during the screening visit period include:

PMP22 gene test: test PMP22 gene amplification. Other screening examinations will be performed after a confirmed diagnosis of CMT1A.

Demographic data: Name, gender, date of birth (age), ethnicity, marriage and childbearing status, height and weight.

Living habits: Smoking and drinking history.

Past medical history, present medical history, treatment history, allergic history and personal history: Past medical history and treatment history of major diseases, present medical history and treatment history of major diseases, history of allergy to drugs/foods, etc., and history of infectious diseases. The disease history in the past 1 year and the medication history within the 3 months before enrollment should be collected (record the name, dose, frequency, start and end dates, and indications). Treatments for CMT1A include surgery, physiotherapy, and drug therapy. The treatment regimen, duration of treatment, and complications should be collected.

Vital signs examination: body temperature (axillary), blood pressure (sitting), and pulse.

Physical examination: skin and mucosa, lymph nodes, head and neck (eyes, oropharynx, thyroid), chest (heart, respiratory system), abdomen, musculoskeletal system and nervous system.

Auxiliary examinations: chest radiography (AP view) or chest CT plain scan, and abdominal B-ultrasound (liver, gallbladder, pancreas, spleen, and both kidneys).

Laboratory tests: hematology, blood chemistry (hepatic and renal function, electrolytes, fasting blood glucose, and glycated hemoglobin), urinalysis, female blood pregnancy test, virological screening (hepatitis B surface antigen, hepatitis B surface antibody, hepatitis B e antigen, hepatitis B e antibody, hepatitis B core antibody, hepatitis C antibody, treponema pallidum antibody, and human immunodeficiency virus antibody), etc. Clinically relevant examination indicators are presented in Appendix 3.

Scale score: Overall Neuropathy Limitation Scale (ONLS), and Charcot-Marie-Tooth Neuropathy Score second version (CMTNS-V2).

Electrophysiological test: sensory and motor nerve conduction of the ulnar and radial nerves on the non-dominant side, including the amplitude of the Compound Muscle Action Potential (CMAP) of the ulnar nerve, the Sensory Nerve Action Potential of the radial nerve. SNAP), the

Nerve Conduction Velocity (NCV) of the ulnar and radial nerves, and the Distant Motor Limit (DML) of the nerves.

D-1: 10-meter walking test (10MWT), nine hole peg test (9HPT) with the non-dominant hand, quantified muscular testing (QMT), 12-lead ECG, ONLS score, CMTNS-V2 score, electrophysiological tests and quality of life evaluation [EuroQol-5 Dimension (EQ-5D)] and laboratory tests (hematology, blood chemistry, urinalysis, and female blood pregnancy test). If the ONLS score, CMTNS-V2 score, and electrophysiological tests are performed within 14 days before randomization, and laboratory tests (hematology, blood chemistry, urinalysis, and female blood pregnancy test) are performed within 7 days before randomization, re-assessment is not required on D-1 and exclusion criteria should be re-checked. Subjects who meet all the inclusion criteria and do not meet any of the exclusion criteria can participate in the study, and those who pass the screening will be randomized, and provided with drugs and diary cards.

7.2 Trial Period (D1–D450)

Administration will be started on D1.

A total of 11 call visits will be conducted in this study on D13 ± 1, D30 ± 7, D60 ± 7, D120 ± 7, D150 ± 7, D210 ± 7, D240 ± 7, D300 ± 7, D330 ± 7, D390 ± 7 and D420 ± 7, respectively, to collect the information on medication, adverse events and concomitant medications of subjects.

D13±1: The investigator should remind the subjects that the dosage is changed from "5 ml/time" to "10 ml/time", and the frequency of administration (twice daily) and oral administration with meals (breakfast and dinner) remain unchanged.

7.2.1 Visit 1 (V1, D90 ± 10)

(1) Collect remaining drugs and diary cards;

(2) Collect adverse events;

(3) Vital signs, physical examination, 12-lead ECG hematology, blood chemistry, female blood pregnancy test and urinalysis;

(4) Record concomitant medication and treatment;

(5) Distribute drugs and diary cards.

7.2.2 Visit 2 (V2, D180 ± 10)

(1) Collect remaining drugs and diary cards;

(2) Collect adverse events;

(3) Vital signs, physical examination, 12-lead ECG hematology, blood chemistry, female blood pregnancy test and urinalysis;

(4) Record concomitant medication and treatment;

(5) Distribute drugs and diary cards;

(6) ONLS scores, CMTNS-V2 scores, 10MWT, 9HPT, QMT, electrophysiological test and EQ-5D.

7.2.3 Visit 3 (V3, D270 ± 10)

(1) Collect remaining drugs and diary cards;

(2) Collect adverse events;

(3) Vital signs, physical examination, 12-lead ECG hematology, blood chemistry, female blood pregnancy test and urinalysis;

(4) Record concomitant medication and treatment;

(5) Distribute drugs and diary cards.

7.2.4 Visit 4 (V4, D360 ± 10)

(1) Collect remaining drugs and diary cards;

(2) Collect adverse events;

(3) Vital signs, physical examination, 12-lead ECG hematology, blood chemistry, female blood pregnancy test and urinalysis;

(4) Record concomitant medication and treatment;

(5) Distribute drugs and diary cards;

(6) ONLS scores, CMTNS-V2 scores, 10MWT, 9HPT, QMT, electrophysiological test and EQ-5D.

7.2.5 Visit 5 (V5, D450 ± 10)

(1) Collect remaining drugs and diary cards;

(2) Collect adverse events;

(3) Vital signs, physical examination, 12-lead ECG hematology, blood chemistry, female blood pregnancy test and urinalysis;

(4) Record concomitant medication and treatment;

(5) ONLS scores, CMTNS-V2 scores, 10MWT, 9HPT, QMT, electrophysiological test and EQ-5D.

7.3 Early Withdrawal

If a subject voluntarily withdraws or is terminated from the study after taking the investigational drug. For the subject's safety, the investigator should try his/her best to ask the subject to complete the safety examination consistent with the withdrawal visit, and record the reason for the subject's withdrawal from the study, withdrawal date, etc. For subjects who withdraw due to AEs, the AEs and follow-up process should be recorded in detail. The outcome of AEs includes recovered/resolved, recovered/resolved with sequelae, improved, persisting, death, or unknown.

7.4 Unscheduled Visit

The investigator may request additional visits or examinations for the subject's safety. Unscheduled visits or examination results should be recorded in the original medical record and eCRF.

8. Trial Evaluation Indicators

8.1 Efficacy Indicators

8.1.1 Primary Efficacy Indicator

The primary efficacy indicator in this study is the improvement in the ONLS score. It is defined as the difference in the ONLS score at Month 15 relative to baseline.

ONLS is a disability scale derived from the Overall Disability Sum Score developed by Grahan and Hughes [12] to measure limited activities of daily living in the arms and legs (Appendix 4). The total score ranges from 0 (no disability) to 12 (highest degree of disability), with a maximum arm score of 5 and a maximum leg score of 7. Although the function of patients with peripheral neuropathy may be affected by other factors in addition to physical strength, ONLS is a measure of the patient's perception of movement and completion of normal life, so it is expected to be related to the quality of life. In CMT1A, a degenerative nervous system disorder, natural history shows a decline in clinical status over time, i.e., an increase in ONLS score over time.

The 136th European Neuromuscular Centre (ENMC) workshop agreed to recommend ONLS as the core disability scale for the CMT1A trial [13], whose reliability will be further validated in 40 CMT patients. Compared with CMTNS, ONLS was shown to be more sensitive to changes [14]. Therefore, ONLS was considered the optimal disability assessment tool and was a clinically relevant endpoint in CMT1A. It was selected as the primary endpoint.

In phase III clinical study of PXT3003 completed in Europe and the United States, the FDA agreed to use the change from baseline in ONLS as the primary endpoint. A decrease of 0.3 in the ONLS with the investigational drug relative to the placebo was considered clinically significant. For the sample size estimation of this study, a true value of 0.3 was assumed for the change in ONLS from baseline.

8.1.2 Secondary Efficacy Indicator

In rare diseases, if there is no ideal endpoint, multiple endpoints can be assessed and their results observed. The following secondary efficacy indicators were selected based on the endpoints selected in phase II and III studies of PXT3003 overseas.

(1) Treatment response rate for PXT3003;

There were 2 response rates for PXT3003 treatment. Treatment response 1 is defined as the proportion of subjects with improvement in ONLS (at least 1 point) at the end of treatment. Treatment response 2 is defined as the proportion of subjects whose ONLS did not worsen at the end of treatment. The higher the response rate, the better the disease stability and the better the effect of drug therapy.

(2) Arm and leg subscale scores in ONLS

The function of the arm involves washing and combing hair, turning the key in the lock, using a knife and fork at the same time, buttoning or zipping, putting on the jacket (excluding buttoning or zipping), and other purposeful activities, which are graded into 6 levels by function and scored 0–5.

The function of the leg involves running or climbing a ladder, walking, gait, walking with aids, use of a wheelchair, purposeful leg activities, and use of protective devices. It is graded into 8 levels by function and scored 0–7.

The lower the arm and leg subscale scores, the better the function and the lower the limitation of daily activities.

(3) CMTNS-V2 Total Score and Subscale Score

The Charcot-Marie-Tooth neuropathy score (CMTNS) is a rating scale designed for peripheral neurological deficits in patients with Charcot-Marie-Tooth disease (Appendix 5) to assess the severity of CMT. It includes 9 items: Motor Symptoms, Sensory Symptoms, Muscle Strength, Pin Sensibility, Vibration, and Nerve Conduction Velocity, each of which is graded into 5 levels and is scored 0–4, 36 points in total. 5 quantified impairments (Sensory Symptoms, Pin Sensibility, Vibration, Strength Arms and Strength Legs), 2 activity limitations (Motor Symptoms Arms and Motor Symptoms Legs), and 2 electrophysiological measures (ulnar compound muscle action potential [CMAP] amplitude and ulnar sensory nerve action potential [SNAP] amplitude). Increased scores indicate deterioration of function: Disability is graded as mild (0–10), moderate (11–20), and severe (21–36). CMTNS has been widely used in the assessment of the severity grading, disease progression, treatment effect, and experimental study of clinical CMT, with good correlation and reliability.

The CMTNS score is an accepted measure of CMT severity and has been used as the primary efficacy indicator in ascorbic acid clinical trials [15]. However, its potential clinical benefit in assessing the drug may be uncertain because it is based on the patient's combined score of signs,

symptoms, and electrophysiological parameters with different meanings and interpretations, making it difficult for objective analysis. Some CMTNS items are clearly not sensitive enough and floor and ceiling effects may occur.

The second version (CMTNS-V2) published in 2011 aims to reduce floor and ceiling effects and standardize patient assessments but it has not been investigated in longitudinal studies. Although the sensitivity of CMTNS to detect the effect of therapeutic interventions has never been demonstrated, it remains the only measure of CMT-specific outcomes (although not specific for CMT1A).

CMTNS-V2 is selected as a secondary efficacy indicator in view of the sensitivity of the likely detected effects and the uncertainty of clinical significance.

(4) Time of 10-meter walking test (10 MWT)

For CMT patients, limited foot dorsiflexion may lead to gait disturbance at the beginning of walking, so a 10-meter walk test (10MWT) is selected to detect the walking speed at small distances instead of endurance at long distances. The result of 10MWT is recorded as the time to walk 10 meters. 10MWT is a simple and standardized test and has been used to assess nervous system disorders and CMT1A in the deterministic evaluation of motor capacity and gait [13,16]. 10MWT has been demonstrated to be reliable in other conditions, but its reliability and sensitivity in CMT have not been confirmed. 10MWT reflects the capacity to walk 10 meters at a constant speed in a controlled environment and is expected to be associated with the ONLS, CMTNS, and QMT.

(5) Nine hole peg test (9HPT) with the non-dominant hand

9HPT is a simple timing test of fine motor coordination of the upper extremities and measures the lag time required for a patient to insert nine pegs in nine holes and remove them (the normal time required is 18 seconds). Although disability function is not directly measured, 9HPT can be considered an injury measurement method and has been widely used in multiple sclerosis [17] and also in CMT clinical trials [13,16]. Intra- and inter-rater reliability was tested in 40 CMT patients with excellent results. However, sensitivity to change has not been assessed and a longitudinal study is required.

Limiting 9HPT to the non-dominant hand is justified because the dominant hand may often be affected by carpal tunnel syndrome or compensation mechanisms associated with CMT disease [18], so 9HPT on non-dominant hands is considered a more specific measure of intrinsic CMT-related status.

(6) Quantified muscular testing (QMT) (grip strength and foot dorsiflexion)

QMT is used to assess exercise intensity in CMT1A [19]. The QMT to test bilateral ankle dorsiflexion and handgrip strength has been successfully used in clinical trials with ascorbic acid for CMT1A, as well as to assess facioscapulohumeral muscular dystrophy (FSHD). Therefore, in this study, bilateral ankle dorsiflexion and bilateral handgrip strength are tested, and the optimal values of 3 consecutive tests are collected. The analysis is based on the average bilateral grip strength and average bilateral dorsiflexion.

(7) Electrophysiological parameters assessing sensory and motor nerve conduction of the ulnar and radial nerves

Electrophysiological parameters included CMAP amplitude of the ulnar nerve, SNAP amplitude of the radial nerve, and NCV and DML of the ulnar and radial nerves.

CMAP measured nerve impulse conduction performance is better than conduction velocity. In CMT1A, conduction velocity is affected from the early stage of onset, and increased CMAP suggests improved symptoms. However, electrophysiological parameters are measured on damaged

and atrophied distal muscles while improvement in symptoms occurs first at the proximal site less affected by the disease, so CMAP has limited sensitivity.

SNAP and NCV have extremely high sensitivity to electrode positioning and are less reliable than motion measurement. SNAP is affected by the skin impedance of thickness changes in subcutaneous tissues. In CMT1A, the conduction velocity cannot be measured if the amplitude is close to zero.

(8) Quality of life evaluation

Quality of life (QoL) evaluations describe how diseases limit life activities and affect overall patient health. There is currently no QoL scale validated in CMT. In the European and American PXT3003 Phase III studies, EQ-5D and Visual Analogue Scale (VAS) were used.

EQ-5D (see Appendix 6 for details) is widely used worldwide as a health-related QoL measure. EQ-5D is used primarily to describe the health status and quality of life of healthy and affected populations. EQ-5D is simple and convenient to use for self-assessment, and EQ-5D Chinese version (mainland) has passed the reliability and validity test.

EQ-5D consists of questionnaire and utility value conversion table. The questionnaire survey results can be used to describe the health status of populations and obtain the EQ-VAS score. The questionnaire can be divided into EQ-5D health description system and EQ-VAS. The EQ-5D health description system includes 5 dimensions: mobility, self-care, usual activities, pain/discomfort, and anxiety/depression. Each dimension has three levels: no problems, some problems, and extreme problems. The EQ-VAS is a 20 cm long vertical visual scale. The top is 100 points, representing the best health condition you can imagine, while the bottom is 0 points, representing the worst health condition you can imagine. The utility value conversion table is used to calculate the EQ-5D index score, which represents the health status of the respondents.

8.2 Safety Evaluation Indicators

Abnormal results and changes from baseline in vital signs, physical examination, laboratory tests, and 12-lead ECG at each visit. The incidence of adverse events (AEs) includes incidence, correlation, and severity of Treatment Emergent Adverse Events (TEAEs); incidence of TEAEs of Grade ≥ 3; incidence of AEs leading to discontinuation of the investigational drug; incidence of Serious Adverse Events (SAEs).

9. Adverse Events and Serious Adverse Events

9.1 Definition of Adverse Event

An adverse event refers to an untoward medical event that occurs after subjects receive the investigational drug until the end of the trial, regardless of causality with the investigational drug. Symptoms/signs that the subjects show during the screening period of the trial will be recorded as medical history. Any worsening of the symptoms/signs during the trial will be recorded as an adverse event.

Adverse events include, but are not limited to the following:

(1) Abnormal laboratory test results with clinical significance;

(2) Symptoms or signs with clinical significance;

(3) Clinically diagnosed as a disease;

(4) Drug allergy, etc.

Significant adverse events refer to any adverse events other than serious adverse events that result in the use of targeted medical measures (e.g., discontinuation, dose reduction, and symptomatic treatment) and other significant abnormalities in hematology or other laboratory tests.

9.2 Treatment Emergent Adverse Event

A treatment emergent adverse event (TEAE) refers to a medical event in subjects not present prior to the initiation of the treatments or an event already present that worsens following exposure to the treatments.

9.3 Determination Criteria for Severity of Adverse Events

All adverse events that occur during the trial are judged for severity according to CTCAE 5.0 (Common Terminology Criteria for Adverse Events).

(1) Grade 1: Mild; asymptomatic or mild symptoms; clinical or diagnostic observations only; intervention not indicated;

(2) Grade 2: Moderate; minimal, local or non-invasive intervention indicated; limiting age-appropriate instrumental ADL (instrumental ADL refers to cooking, buying clothing, using telephone, wealth management, etc.).

(3) Grade 3: Severe or medically significant but not immediately life-threatening; hospitalization or prolongation of hospitalization indicated; disabling; limiting self-care ADL (self-care ADL refers to bathing, dressing and undressing, feeding self, using the toilet, taking medications, but not bedridden);

(4) Grade 4: Life-threatening; requiring emergency treatment;

(5) Grade 5: Death related to AEs.

9.4 Judgment Criteria for Relationship between Adverse Events and Investigational Drug

The following factors should be considered comprehensively for the analysis of the correlation between the adverse event and the investigational drug:

(1) Whether there is a sequential or causal relationship between the drug administration time and the time of occurrence of adverse events;

(2) Whether the adverse event conforms to the known type of adverse reaction of such drug;

(3) Whether the adverse event can be explained by concomitant medication, the clinical status of the patient or other treatments;

(4) Whether the adverse event disappears or is alleviated after drug withdrawal or dose reduction;

(5) The reaction occurs after the drug is resumed.

According to the judgment criteria of the causality between drugs and adverse events, the relationship between adverse events and the investigational drug is divided into five categories: definitely related, probably related, possibly related, unlikely related, and not related. Adverse events that are definitely related, probably related and possibly related to investigational drugs are listed as adverse drug reactions (ADRs).

Definitely related: The adverse event conforms to the known reaction type of the suspected drug, follows a reasonable temporal sequence from the administration of the drug, is relieved or resolved after dose reduction or discontinuation, and recurs after rechallenge.

Probably related: The adverse event conforms to the known reaction type of the suspected drug, follows a reasonable temporal sequence from the administration of the drug, and is relieved or disappears after dose reduction or discontinuation. However, the clinical status of subjects or other causes may also lead to the adverse event.

Possibly related: The adverse event conforms to the known reaction type of the suspected drug, follows a reasonable temporal sequence from the administration of the drug, and is relieved or not

obvious after dose reduction or discontinuation. However, the clinical status of subjects or other causes can account for the adverse event.

Unlikely related: The adverse event does not conform to the known reaction type of the suspected drug, does not follow a reasonable temporal sequence from the administration of the drug, and the clinical status of subjects or other causes may also lead to the adverse event.

Not related: The adverse event does not conform to the known reaction type of the suspected drug, and does not follow a reasonable temporal sequence from the administration of the drug; the clinical status of subjects or other causes can also account for this reaction. After the elimination of clinical symptoms or other causes, this adverse event is relieved or disappears.

Table 6 Judgment of Causality with Drug

	1	2	3	4	5
Definitely related	+	+	-	+	+
Probably related	+	+	-	+	?
Possibly related	+	+	±	±	?
Unlikely related	+	-	±	±	?
Not related	-	-	+	-	-

Note: + indicates positive, - indicates negative, ± indicates positive or negative; ? indicates an unclear situation.

9.5 Identification and Follow-up of Adverse Events

Adverse events are learned and recorded in the source documents and eCRFs through active reporting by subjects, oral questions by investigators, and review of concomitant medications.

Any adverse events, such as subjective discomfort and laboratory test abnormalities, should be taken seriously and analyzed carefully, and corresponding measures should be taken immediately to ensure the safety of subjects. All adverse events should be followed until the subjects are recovered, returned to baseline, stabilized, reasonably explained, died, or lost to follow-up, and the processing process, follow-up data, and outcome should be fully recorded in the source documents and eCRF.

9.6 Requirements for Reporting Adverse Events

The investigator should evaluate each adverse event to determine whether it meets the criteria for serious adverse events and the causality with the drug. If serious adverse events occur, they should be reported in a timely manner in accordance with national regulations.

All adverse medical events (serious and non-serious) that occur from the time the subject receives the investigational drug until the end of the trial should be recorded in the eCRF and reported in standardized medical terms.

9.7 Definition of Serious Adverse Event

Serious adverse events refer to deaths, life-threatening events, events requiring hospitalization or prolonged hospitalization, permanent or serious disability or loss of function, congenital anomalies or birth defects, and other important medical events after the subject receives the investigational drug. If the above situations may occur without treatment, it needs to be decided based on medical judgment.

9.8 Requirements for Reporting Serious Adverse Events

In case of a serious adverse event occurring during the trial, the investigator should report to the Sponsor as soon as possible within 24 h upon identification or awareness, and the death case should be reported immediately. The investigator should report as per the Sponsor's version of the SAE Report Form. The initial SAE report should include the following contents: source of the report, basic information of the subject, name of investigational drug, name of the serious adverse event, onset time, duration, severity, causality with the investigational drug, actions taken for the

investigational drug, treatment and outcome of the event, etc., and the report form should be signed. Follow-up information should be reported in a timely manner.

9.9 Suspected Unexpected Serious Adverse Reaction

Suspected unexpected serious adverse reaction (SUSAR) refers to any SUSAR in which the nature and severity of clinical manifestations exceed the scope in existing documents such as the Investigator's Brochure of investigational drug, the package inserts or product summary of the drug launched onto the market already.

According to ICH guidelines on pharmacovigilance (E2A, E2B, and M1) and the Standards and Procedures for Rapid Reporting of Safety Data during Drug Clinical Trial issued by the Center for Drug Evaluation, all unexpected and serious adverse reactions definitely related to or suspected with the investigational drug occur during the clinical trial, as well as other circumstances specified in the standards and procedures, should be reported to the national drug evaluation agency in an expedited manner within the prescribed time limit.

The expected evaluation of the SUSAR report refers to the following four aspects: (1) whether the adverse event is listed in the Investigator's Brochure (IB); (2) although the adverse event is listed in the IB, whether its severity is consistent (for example, the severity of the listed adverse event is mild, but the reported adverse event has led to hospitalization); (3) although the adverse event is listed in the IB, it occurs more frequently than that described in the IB; (4) other circumstances.

The relevant evaluation of the SUSAR report should refer to the five principles followed by the National Center for ADR Monitoring, China: (1) Whether there is a reasonable temporal relationship between medication and the occurrence of adverse events; (2) Whether the reaction conforms to the known type of adverse reaction with the drug; (3) Whether the reaction disappears or is reduced after drug withdrawal or dose reduction; (4) Whether the same reaction event occurs again when the suspected drug is resumed; (5) Whether the reaction can be explained by the effect of concomitant medication, the progression of the patient's condition, and the effect of other treatments.

If the sponsor judges the SAE as SUSAR, it should be reported to the Drug Registration Department of the National Medical Products Administration and the Bureau of Medical Administration, National Health Commission of the People's Republic of China through electronic transmission as required by E2B (R3), and reported to the investigator in a common version. The investigator will report SUSAR with reference to the requirements of the site.

For fatal or life-threatening SUSARs, the sponsor should report as soon as possible but not more than 7 days after the first awareness, and report and complete the follow-up information within the next 8 days; for non-fatal or non-life-threatening SUSARs, the sponsor should report as soon as possible but not more than 15 days after the first awareness.

9.10 Reporting of Pregnancy

All subjects (males and females) are required to take strict contraception in this study.

After learning that a female subject or partner of a male subject who has received at least one dose of the investigational drug from the first dose to 1 month after the last dose is pregnant, the investigator should collect the corresponding pregnancy records and report them to the Ethics Committee and the sponsor.

During the trial, female subjects found pregnant should withdraw from the trial immediately while male subjects with a pregnant partner are not required to withdraw from the trial.

After learning that a female subject or partner of a male subject is pregnant, the investigator should report to the sponsor and the Ethics Committee within 24 h (the reporting route is the same

as that of SAE), follow up on the pregnancy outcome (e.g., termination or delivery), and improve the reporting process of pregnancy outcome in clinical trials.

Pregnancy itself is not an AE or SAE, but any complication during pregnancy or termination of pregnancy due to medical reasons will be recorded, reported, and followed up as "AE" or "SAE" as specified in the protocol. If the pregnancy outcome meets the criteria of SAE, such as spontaneous abortion (including threatened abortion, inevitable abortion, incomplete abortion, complete abortion, and missed abortion), stillbirth, neonatal death, or congenital malformation, the investigator should report it according to the reporting procedure of SAE.

All neonatal deaths that occur within one month of birth should be reported as an SAE, regardless of cause. In addition, any infant death after the first month of life should be reported, as long as it is considered by the investigator to be possibly related to the investigational drug.

10. Risk Management and Risk Assessment

10.1 Adverse Reactions of Individual Pharmaceutical Ingredients in PXT300**3**

The three main ingredients of PXT3003 have been marketed for many years. (RS)-baclofen was approved by the FDA in 1977 for the treatment of skeletal muscle spasms induced by different diseases and was marketed in China in 1998; Naltrexone hydrochloride was approved by the FDA in 1984 for the treatment of opioid and alcohol dependence and was marketed in China in 2000; D-sorbitol was approved by the FDA for the treatment of constipation in 1978, and is widely used worldwide as a medical excipient and food additive. The dose of (RS)-baclofen, naltrexone hydrochloride, and D-sorbitol in PXT3003 is 1/35–1/7 of the clinically approved dose of them as a single drug, so the risk of adverse reactions of individual pharmaceutical ingredients in PXT3003 is low.

10.1.1 (RS)-baclofen

Adverse reactions of baclofen mainly occur in patients at the beginning of treatment or after a rapid dose increase and excessive dose and are generally mild transient symptoms. Adverse reactions may be severer in patients with a history of psychosis, patients complicated with cerebrovascular disease, and elderly patients.

(1) Nervous system: Sedation and drowsiness often occur at the beginning of treatment, with occasional dry mouth, respiratory depression, confusion, dizziness, headache, insomnia and fatigue.

(2) Neurology or psychiatry: occasional: euphoria, depression, paresthesia, myalgia, muscle weakness, ataxia, tremor, nystagmus, hallucinations, and nightmares.

(3) Gastrointestinal tract: common: nausea; occasional: mild digestive dysfunction (vomiting, constipation, diarrhea); rare: abdominal pain.

(4) Cardiovascular system: common: hypotension and decreased cardiac output.

(5) Urogenital system: common: dysuria, frequent urination and enuresis; rare: urinary retention and erectile dysfunction.

(6) Skin and subcutaneous tissue: common: hyperhidrosis and rash.

(7) Other side effects: visual impairment, paresthesia, dysgeusia, dysarthria, hepatic impairment, and hypothermia in rare or individual cases.

(8) Special warnings and precautions: Use with caution in patients with gastrointestinal ulcer disease (including medical history), hepatic and renal dysfunction, and cerebrovascular disease. In patients with psychiatric disorders, schizophrenia, depression or mania, Parkinson's disease, and confusion, baclofen should be used with extreme caution as it may cause exacerbations. In patients with epilepsy, baclofen lowers the threshold for convulsions and may cause them.

Baclofen may cause dizziness, sedation, somnolence, and visual disturbances, affecting the patient's ability to react. Elderly patients (aged 65 and above) are prone to experience adverse reactions.

(9) Drug interactions: Baclofen may increase the side effects of levodopa/carbidopa (insanity, hallucinations, headache, nausea, and irritability). Baclofen may enhance sedation when used in combination with CNS depressants, synthetic opioids or alcohol. Tricyclic antidepressants may potentiate the effects of baclofen, causing marked hypotonia. Baclofen combined with lithium can aggravate hyperkinesia. Combination with antihypertensive drugs may enhance the antihypertensive effect, so the dosage of antihypertensive drugs needs to be adjusted.

10.1.2 Naltrexone hydrochloride

After the first dose of 75 mg naltrexone hydrochloride in the normal and healthy population, a few people developed nausea, vomiting, gastrointestinal discomfort, anorexia, and fatigue, and the symptoms gradually disappeared after continuous administration for 2 or 3 days.

Adverse reactions with an incidence of more than 10%: Difficulty sleeping, anxiety, irritability, abdominal pain/spasm, nausea, vomiting, arthralgia, myalgia, and headache.

Adverse reactions with an incidence of less than 10%: Inappetence, diarrhea, constipation, thirst, and dizziness.

Adverse reactions with an incidence of less than 1%:

(1) Respiratory system: Nasal congestion, runny nose, sore throat, hoarse voice, cough, shortness of breath;

(2) Cardiovascular system: Elevated blood pressure, edema, non-specific ECG changes, palpitations, tachycardia, and phlebitis.

(3) Gastrointestinal tract: Excessive gas generation, hematochezia, diarrhea and ulcer.

(4) Musculoskeletal system: Pain and tremors in shoulders, lower limbs and knees.

(5) Skin: Pruritus, acne, and sores.

(6) Genitourinary system: Discomfort in urination and decreased libido

(7) Mental: Depression, paranoia, fatigue, restlessness, insanity, hallucinations, and nightmares.

(8) Contraindications: Those who use opioid analgesics; do not abstain from opioid addiction; suddenly discontinue opioids; fail to stimulate naloxone hydrochloride (positive); are positive for opioids in urine test; suffer from acute hepatitis or hepatic failure and insufficiency; are allergic to naltrexone hydrochloride.

(9) Precautions: Hepatotoxicity. Daily doses up to 300 mg can cause hepatocyte damage, resulting in elevated transaminases. Patients should be free of opioids for at least 7 to 10 days prior to treatment with naltrexone hydrochloride. Opioid analgesics should not be used in patients taking naltrexone hydrochloride when analgesics are required under contraindications. During the administration of naltrexone hydrochloride, high doses of opioids may cause severe toxic symptoms, coma, or death.

10.1.3 D-sorbitol

Oral sorbitol is mainly used for dyspepsia, abdominal distension, inappetence, and constipation. Treatment of dyspepsia, abdominal distension, and inappetence: oral administration of 2–4 g/time, TID; treatment of constipation: 6–10 g each time. This product has minimal side effects and can be taken for a long time, while the daily dose of sorbitol in PXT3003 is much lower than the therapeutic dose for dyspepsia.

10.2 Safety Report during Drug Development

1. PXT3003 Phase II Study

In the PXT3003 Phase II study, PXT3003 was safe and well tolerated. The proportion of drug-related TEAEs was similar in the low-dose group, medium-dose group, and high-dose group. Most TEAEs were mild and moderate. The therapeutic drug had no significant effect on blood pressure, heart rate, weight, ECG, and laboratory tests (Table 7).

Table 7 Drug-related TEAEs in Treatment Group of PXT3003 Phase II Study (reported at ≥ 2%)

	High-Dose Group (Dose 3) (N=19)	Medium-Dose Group (Dose 2) (N=21)	Low-Dose Group (Dose 1) (N=21)
	N (%)	N (%)	N (%)
All AEs	6 (31.6%)	7 (33.3%)	5 (23.8%)
Gastrointestinal disorders	4 (21.1%)	1 (4.8%)	1 (4.8%)
Nausea	2 (10.5%)	1 (4.8%)	0
Abdominal pain	2 (10.5%)	0	0
Diarrhea	1 (5.3%)	0	0
Nervous system disorders	3 (15.8%)	2 (9.5%)	2 (9.5%)
Dizziness	1 (5.3%)	0	2 (9.5%)
Headache	0	2 (9.5%)	0
Lethargy	2 (10.5%)	0	0
Systemic symptoms	0	1 (4.8%)	1 (4.8%)
Fatigue	0	1 (4.8%)	1 (4.8%)
Musculoskeletal and connective tissue disorders	1 (5.3%)	1 (4.8%)	1 (4.8%)
Muscle spasm	1 (5.3%)	1 (4.8%)	0
Renal and urinary disorders	1 (5.3%)	0	0

2. PXT3003 Phase III

(1) TEAEs in Phase III Study

A total of 348 TEAEs were reported by 87 patients in the Dose 4 group, 476 TEAEs by 89 patients in the Dose 3 group, and 359 TEAEs by 83 patients in the placebo group (Table 8).

Table 8 Summary of Adverse Events of PXT3003 Phase III Study

	Dose 4 **(N=113)**	Dose 3 **(N=109)**	**Placebo (N=101)**
All TEAEs	87(77.0%)	89(81.7%)	83(82.2%)
All relevant TEAEs	38(33.6%)	39(35.8%)	34(33.7%)
All moderate to severe or severe-related TEAEs	5(4.4%)	8(7.3%)	10(9.9%)
All TEAEs leading to drug withdrawal	6(5.3%)	6(5.5%)	6(5.9%)
All related TEAEs leading to drug withdrawal	2(1.8%)	3(2.8%)	2(2.0%)
All serious TEAEs	3(2.7%)	10(9.2%)	5(5.0%)
All relevant serious TEAEs	0(0.0%)	0(0.0%)	0(0.0%)
All serious TEAEs leading to drug withdrawal	0(0.0%)	1(0.9%)	0(0.0%)

(2) Drug-related TEAEs by SOC

Eighty-one drug-related TEAEs were reported by 38 patients in the Dose 4 group and 105 drug-related TEAEs were reported by 39 patients in the Dose 3 group (Table 9).

Table 9 Drug-related TEAEs Reported at ≥ 2% of Treatment Groups by SOC and PT

System organ class	Dose 4 (N=113)		Dose 3 (N=109)	
Preferred Term	N (%)	Number of Events	N (%)	Number of Events
Any related TEAE	38 (33.6%)	81	39 (35.8%)	105
Gastrointestinal disorders	22 (19.5%)	25	17 (15.6%)	28
Nausea	6 (5.3%)	6	7 (6.4%)	7
Diarrhea	6 (5.3%)	6	3 (2.8%)	3
Dry mouth	3 (2.7%)	3	2 (1.8%)	2
Constipation	1 (0.9%)	1	3 (2.8%)	3
Nervous system disorders	11 (9.7%)	20	12 (11.0%)	17
Dizziness	3 (2.7%)	3	4 (3.7%)	5
Headache	6 (5.3%)	10	6 (5.5%)	6
Somnolence	1 (0.9%)	1	5 (4.6%)	5
Systemic disorders and administration site symptoms	5 (4.4%)	5	7 (6.4%)	7
Fatigue	1 (0.9%)	1	6 (5.5%)	9
Musculoskeletal and connective tissue disorders	8 (7.1%)	8	5 (4.6%)	7
Muscle spasm	3 (2.7%)	3	0 (0%)	0
Pain joint	1 (0.9%)	1	3 (2.8%)	4
Skin and subcutaneous tissue disorders	5 (4.4%)	5	5 (4.6%)	7
Infection	3 (2.7%)	4	5 (4.6%)	11
Nasopharyngitis	3 (2.7%)	4	4 (3.7%)	6
Psychiatric disorders	3 (2.7%)	3	4 (3.7%)	5
Ear and labyrinth disorders	1 (0.9%)	1	3 (2.8%)	3
Respiratory, thoracic and mediastinal disorders	0 (0%)	0	3 (2.8%)	6

SOC=system organ class; PT = preferred term; N = number of patients in the treatment group; n = number of patients reporting events; percentages are calculated based on N.

(3) Moderate or severe drug-related TEAEs

Eight drug-related moderate or severe TEAEs were reported in the Dose 4 group and 24 drug-related moderate or severe TEAEs were reported in the Dose 3 group (Table 10).

Table 10 Drug-related moderate or severe TEAEs

System organ class	Dose 4 (N=113)		Dose 3 (N=109)	
Preferred Term	N (%)	Number of Events	N (%)	Number of Events
Any moderate or severe related TEAE	5 (4.4%)	8	8 (7.3%)	24
Gastrointestinal disorders	2 (1.8%)	2	4 (3.7%)	6
Anal hemorrhage	0 (0%)	0	1 (0.9%)	1
Constipation	0 (0%)	0	1 (0.9%)	1
Diarrhea	1 (0.9%)	1	2 (1.8%)	2
Dyspepsia	1 (0.9%)	1	0 (0%)	0
Nausea	0 (0%)	0	1 (0.9%)	1
Vomiting	0 (0%)	0	1 (0.9%)	1
Nervous system disorders	1 (0.9%)	1	4 (3.7%)	4
Dizziness	0 (0%)	0	1 (0.9%)	1

System organ class	Dose 4 (N=113)		Dose 3 (N=109)	
Headache	1 (0.9%)	1	2 (1.8%)	2
Lethargy	0 (0%)	0	1 (0.9%)	1
Systemic symptoms	1 (0.9%)	1	0 (0%)	0
Flu-like symptoms	1 (0.9%)	1	0 (0%)	0
Musculoskeletal and connective tissue disorders	2 (1.8%)	2	2 (1.8%)	8
Muscle spasm	1 (0.9%)	1	0 (0%)	0
Pain joint	1 (0.9%)	1	2 (1.8%)	3
Back pain	0 (0%)	0	2 (1.8%)	2
Neck pain	0 (0%)	0	1 (0.9%)	1
Pain in extremity	0 (0%)	0	1 (0.9%)	2
Psychiatric disorders	1 (0.9%)	1	2 (1.8%)	2
Anxiety	0 (0%)	0	1 (0.9%)	1
Mental aberration	0 (0%)	0	1 (0.9%)	1
Sleep disorder	1 (0.9%)	1	0 (0%)	0
Infection	0 (0%)	0	1 (0.9%)	1
Nasopharyngitis	0 (0%)	0	1 (0.9%)	1
Ear and labyrinth disorders	0 (0%)	0	1 (0.9%)	1
Ear ache	0 (0%)	0	1 (0.9%)	1
Respiratory, thoracic and mediastinal disorders	0 (0%)	0	1 (0.9%)	1
Cough	0 (0%)	0	1 (0.9%)	1
Skin and subcutaneous tissue disorders	1 (0.9%)	1	0 (0%)	0
Seborrhoeic dermatitis	1 (0.9%)	1	0 (0%)	0
Metabolism and nutrition disorders	0 (0%)	0	1 (0.9%)	1
Appetite increased	0 (0%)	0	1 (0.9%)	1

(4) Serious adverse event (SAE)

Three patients in the Dose 4 group experienced SAEs: infectious ileitis, proctitis, and right distal ureteral calculus with third-degree hydronephrosis, all of which were unrelated to the investigational drug.

SAEs occurred in 10 patients in the Dose 3 group and were unrelated to the investigational drug. The SAEs are hospitalization for left patella fracture, obstructive sleep apnea, appendicitis, accidental cut of right wrist, right lobe and isthmus resection for thyroid nodules in the right lobe, resection of basal cell epithelioma of the back, cholecystectomy for cholecystolithiasis with cholecystitis, hospitalization for seizures, hysterectomy for right adnexal mass and hysteromyoma + right salpingo-oophorectomy + left salpingectomy and adhesiolysis, and supraventricular tachycardia.

There are no deaths during the study.

10.3 Risk Management Plan

For subject screening, the inclusion and exclusion criteria must be strictly followed, and the Informed Consent Form should clearly inform the subjects of the possible risks of participating in this study. Common safety risks in clinical studies require close attention and treatment if necessary.

(1) Risk of gastrointestinal disorders

The risks include nausea, diarrhea, dry mouth, constipation, dyspepsia, and abdominal pain. Close observation and symptomatic treatment are required during the study.

(2) Risk of nervous system disorders

The risks include dizziness, headache, and somnolence. The subjects should be fully informed before the study to avoid falls and injuries and vital signs during the study should be monitored.

(3) Systemic symptoms

The risks include fatigue and asthenia, which should be closely observed during the study.

(4) Risk of musculoskeletal and connective tissue disorders

The risks include muscle spasms and arthralgia, which should be given symptomatic treatment during the study.

(5) Skin and subcutaneous tissue disorders

Close observation is needed during the study and a visit to the Department of Dermatology will be required if necessary.

(6) Nasopharyngitis

Symptomatic treatment should be given during the study.

(7) Psychiatric disorders

Close observation and timely intervention are required during the study.

(8) Tinnitus

Close observation is required during the study.

(9) Nutritionist can be consulted for metabolism and nutritional disorders.

11. Data Management

The purpose of data management is to ensure the reliability, completeness, and accuracy of data. The goal is to obtain high-quality real-world data for statistical analysis. This study adopts electronic case report forms (eCRFs) for the collection and management of study data.

11.1 Data Collection

Electronic data management and Taimei Medical Management System are adopted in this trial. The data manager designs and establishes the data management according to the trial protocol, sets logic verification according to the data verification plan (DVP), and releases it for use after passing the test and obtaining the approval of the sponsor. The eCRF data are derived from the original records, and the data entry personnel should enter the subject visit data into EDC in a timely manner according to the instructions for eCRF filling.

11.2 Database Management and Quality Control

As a guidance document for data management, the Data Management Plan (DMP) is prepared by the data manager (DM) and approved by the sponsor. The data management is carried out according to the relevant standard operating procedure (SOP) and DMP.

11.3 Data Review and Monitoring of Study Site

(1) Source data verification (SDV): The monitor checks the consistency between the eCRF data and the source data. Questions can be raised if any.

(2) Data-related questions and answers: Questions from systems such as EDC data validation and from persons such as monitor and DM should be answered by investigators in a timely manner. The DM and the monitor should review and approve these answers and pose questions again, if any, until the data are "clean".

(3) Signature by investigators: After data entry is finished and SDV is performed, investigators perform an electronic signature review for confirmation. In case of any data revision after signing, re-signing is required.

(4) Database lock: After the Sponsor, statistical analyst and DM signed on the database lock log, the DM will lock the database.

(5) Database submission: The data manager sends the data to the statistician in the form of an encrypted read-only SAS dataset.

(6) eCRF archiving: The eCRFs for each subject should be archived as PDF electronic documents.

(7) EDC closure: After the completion of statistical analysis, the DM will apply to the system for closing the database.

12. Statistical Analysis

12.1 Statistical Hypothesis

Primary efficacy hypothesis:

H_0: The primary efficacy endpoint of PXT3003 for the treatment of Charcot-Marie-Tooth Disease type 1A for 15 months is not different from that of the control group.

H_1: The primary efficacy endpoint of PXT3003 for the treatment of Charcot-Marie-Tooth Disease type 1A for 15 months is superior to that of the control group.

12.2 Definition of Analysis Population

Full analysis set (FAS): All randomized subjects who provided baseline and at least one post-baseline ONLS score. The evaluation of primary and secondary efficacy is based on FAS and analyzed according to the grouping at randomization.

Per protocol set (PPS): All subjects in FAS (defined during the blinded review period) with no major protocol deviations impacting efficacy from baseline to the Month 15 visit. The PPS will be used for the evaluation of primary and secondary efficacy to support FAS-based results.

Safety set (SS): All randomized subjects who received at least 1 dose of the investigational drug. Subjects are analyzed according to the treatment actually received. All safety analyses are performed on the safety set.

12.3 Statistical Methods

12.3.1 General Principles

Continuous variables will be described using arithmetic means, standard deviation (SD), median, minimum, and maximum. Categorical variables will be described using the number and percentage of cases.

Efficacy analysis will be performed in FAS and PPS, with the results mainly based on FAS. Safety analyses are performed on the safety set.

Except in special circumstances, the table is only for scheduled visits, and the listing lists all visits including unscheduled visits.

The specific statistical methods will be described in the Statistical Analysis Plan.

12.3.2 Analysis of Primary Efficacy Endpoint

The primary efficacy endpoint is defined as the difference in the ONLS score at Month 15 relative to baseline. The primary efficacy endpoint is analyzed using an ANCOVA model with covariates including baseline value and fixed effects including treatment group. The main effect of the treatment group will be reported along with its 95% confidence interval and p-value.

12.3.3 Analysis of Secondary Efficacy Endpoint

The same analysis method as for the primary efficacy endpoint will be used for the difference in ONLS score at Month 6 and Month 12 relative to baseline.

For the secondary efficacy indicators involving proportion, the 95% CI of the two response rates is calculated by the Clopper-Pearson method, and the comparison between the two groups is performed by the chi-square test.

The statistical analysis methods used for the analysis of the following secondary efficacy indicators are the same as those used for the primary efficacy indicators: arm and leg subscale scores in ONLS, CMTNS-V2 total score, CMTNS-V2 arm item score, CMTNS-V2 leg item score at each time point; 10MWT time; 9HPT time; Quantified Muscular Testing (QMT) (grip strength and foot dorsiflexion), electrophysiological parameters of sensory and motor nerve conduction of the ulnar and radial nerves.

The quality of life of patients will be assessed by EQ-5D and EQ-VAS, and the scores of EQ-5D and EQ-VAS at each visit and their changes from baseline will be statistically described.

12.3.4 Safety Analysis

Adverse events will be coded according to the MedDRA and graded according to CTCAEV5.0.

Adverse events that occur during or after the administration of the investigational drug, including TEAEs, investigational drug-related TEAEs, TEAEs of Grade ≥ 3, TEAEs leading to treatment discontinuation, TEAEs leading to death, serious adverse reactions, SAEs, and investigational drug-related SAEs, will be summarized for the number and percentage of patients by system organ classification (SOC), preferred term (PT) and group. In addition, the severity of TEAEs will also be summarized by SOC, PT and group. The proportion of subjects with at least 1 adverse event (multiple consecutive occurrences of the same event will be counted as 1 event by maximum severity) will be reported.

Deaths reported during study treatment and in the follow-up period after treatment completion/discontinuation will be summarized.

The test values and changes from the baseline of safety indicators including specific laboratory tests, physical examination, vital signs and ECG will be summarized. Where applicable, the baseline examination results and the examination results at various time points after baseline should be presented in a cross table.

12.3.5 Procedures for Handling Missing Data

When the primary analysis of efficacy endpoints is based on FAS, missing values are imputed based on predictions from a linear model built on the placebo group data, and extrapolated from the results of multiple imputations. See the statistical analysis plan for specific methods.

12.4 Interim Analysis

An independent data monitoring committee (IDMC) will be established for this study and interim analyses will be performed. The composition, responsibilities, and implementation details of the IDMC will be specified in the IDMC Charter. The interim analysis will be conducted when 88 subjects complete the Month 15 ONLS assessment for the purpose of a superiority test of ONLS scores and sample size re-estimation. An increase in sample size may be considered if deemed necessary by the IDMC. The Lan-Demets approach is used in the Group Sequential Design, and O'Brien-Fleming α-spending function is used to control the overall type I error rate at 0.05: if the p-value of the interim analysis is ≤ 0.003, the trial will be stopped for significant superiority. If the p-value of the interim analysis is > 0.003, the trial continues. The final analysis will be performed when all subjects complete the final assessment at a significance level of 0.049.

The actual α-spending and significance level for each analysis will be adjusted for the exact sample size actually observed at the time of analysis.

12.5 Statistical Software

All statistical analyses will be performed by SAS software (version 9.4 or above).

13. Ethical Norms and Informed Consent

13.1 Ethics Committee

This trial is conducted in accordance with Good Clinical Practice (GCP), the Declaration of Helsinki, relevant laws and regulations, and the review comments of the Ethics Committee. The investigator should ensure that the trial is reviewed and approved by a qualified Ethics Committee in compliance with GCP. Prior to the trial, the investigator should submit a trial protocol, informed consent form and other necessary materials to the Ethics Committee for review and approval. The Sponsor can provide the investigational drug only after receiving the Ethics Committee's approval. Meanwhile, the Ethics Committee must be informed of any subsequent protocol amendments that may affect the safety of the subjects and their continued participation in the trial and of any SAEs that occur during the trial. The investigator should report the progress of the trial to the Ethics Committee. In addition, the investigator must submit a copy of all correspondence with the Ethics Committee to the Sponsor in a timely manner. When reviewing and approving the trial protocol, the Ethics Committee must confirm the protocol title and protocol number, and indicate the reviewed protocol documents and review date. During the trial, if there is any new amendment to the trial protocol, informed consent form, etc., another written approval of relevant management organizations should be obtained according to the regulations.

13.2 Informed Consent

The Investigator must inform the subjects, legal representatives of subjects (if necessary), and guardians of subjects under the age of 18 of the details of the study, both verbally and in writing.

The informed consent form (along with the trial protocol) must be reviewed and approved by the Ethics Committee. It is the responsibility of the investigator to explain the contents of the informed consent form to the subjects in an understandable manner, if necessary. Subjects, legal representatives of subjects, and guardians of subjects under the age of 18 should be given sufficient time to read before formally signing the informed consent form.

The final text of the informed consent form should contain the following: trial objective, process and duration of the trial, examination operations, expected possible benefits and risks, and different groups of the trial to which the subjects may be assigned; treatment and corresponding compensation available to the subjects in case of trial-related damage; confidentiality principle of subject's personal information.

The informed consent form for subjects under the age of 18 must be signed by the subject, the subject's legal guardian or legal representative; the informed consent form for subjects aged 18 years and above must be signed and dated by the subject or the subject's legal representative. The investigator implementing the informed consent process must also sign and date the informed consent form. The informed consent form is also allowed to be signed by an independent witness who can prove that the subject agrees to participate in the trial. The investigator and subject should keep 1 copy of the informed consent form, respectively. If there is important new information involving the investigational drug, the informed consent form must be revised and submitted to the Ethics Committee for approval, and consent of the subject should be obtained again.

13.3 Subject Confidentiality

It is the responsibility of the investigator to maintain the anonymity of the subjects. The CRFs or other documents can identify the subjects only with capital letters, numbers and/or codes, but not

the subject's name. The investigator must keep a subject enrollment log documenting the subject code, name and home address. The investigator must maintain strict confidentiality of documents identifying the subjects.

14. Quality Control and Assurance

The drug clinical trial institution has standard operating procedures and quality control procedures related to the subject's informed consent, screening, drug administration, blood collection, etc.

Acumen Medical Information & Technology Co., Ltd., a data management company, should provide standard operating procedures and quality control procedures related to data entry and verification, data transmission and data quality assurance. There should be dedicated personnel who can act as the project quality controllers to implement quality control according to the internal quality control procedures.

Acumen Medical Information & Technology Co., Ltd. will provide a pharmacovigilance system for the study and assist in reporting possible serious adverse events and suspected unexpected serious adverse reactions during the trial.

Acumen Medical Information & Technology Co., Ltd. should provide standard operating procedures and quality control procedures related to statistical analysis plans and statistical analysis reports. The Head of Statistical Analysis should designate a person as the statistical analysis quality controller of the project to implement quality control according to the internal quality control procedures.

Genenet Pharmaceutical Co., Ltd will formulate an audit plan according to the project progress and conduct the audit as planned.

15. Handling of Special Conditions during the Trial

15.1 Termination Criteria

Under the premise of fully protecting the rights & interests and safety of subjects, the Sponsor, Ethics Committee or regulatory authorities require the termination of the study.

15.2 Protocol Deviations or Violations

All requirements specified in the study protocol should be strictly implemented. Any behavior that intentionally or unintentionally deviates from or violates the study protocol and the principles of GCP should be considered a protocol deviation or protocol violation During monitoring, if the monitor finds a protocol deviation, the investigator or monitor should fill in the Protocol Deviation Record to record the time when the deviation is found, the time and process of the event, the cause and corresponding handling measures. The record should be signed by the investigator and reported to the Ethics Committee and the Sponsor according to the relevant regulations of the study site. In the data statistics and summary report, the investigator and the Sponsor should analyze, assess and report the impact of protocol deviations or violations on the final data and conclusions.

In case of any significant protocol violation, an assessment should be carried out. If necessary, the Sponsor can terminate the study in advance.

16. Protocol Revision

Any amendment related to the study protocol should be determined by the Sponsor and the clinical study site through negotiation, and should come into effect after being approved by the Ethics Committee or filed. All amendments to the protocol must be recorded in the protocol amendment form, which will list the differences between this amendment and the previous version. During the operation of the clinical trial, if it is found that the design of the study protocol poses a hazard to the safety of the subjects, the Sponsor and the Ethics Committee should be notified as

soon as possible, and the protocol revision can take effect first, and then the EC approval or filing procedures can be supplemented.

17. Data Archiving

To ensure the evaluation and supervision of the clinical study by the NMPA and the Sponsor, the investigator should agree to archive all study data, including original records of subject hospitalization, ICFs, CRFs, and detailed records of drug distribution. The investigator should keep the data until 5 years after the end of this clinical trial or 5 years after the investigational drug is marketed (whichever is the longest). All the data of this clinical study are proprietary to Genenet Pharmaceutical Co., Ltd Unless required by the Food and Drug Administration at all levels, the investigator should not provide the data to any third party in any form without the written consent of the Sponsor.

18. References

[1] Barreto LC, Oliveira FS, Nunes PS et al. Epidemiologic Study of Charcot-Marie-Tooth Disease: A Systematic Review. Neuroepidemiology. 2016;46(3):157-65.

[2] Pareyson D, Marchesi C. Diagnosis, natural history, and management of Charcot-Marie-Tooth disease. Lancet Neurol. 2009;8(7):654-67.

[3] Abe A, Numakura C, Kijima K et al. Molecular diagnosis and clinical onset of Charcot-Marie-Tooth disease in Japan. J Hum Genet. 2011;56(5):364-8.

[4] Wang R, He J, Li JJ et al. Clinical and genetic spectra in a series of Chinese patients with Charcot-Marie-Tooth disease. Clin Chim Acta. 2015;451(Pt B):263-70.

[5] Wu R, Lv H, Zhang W et al. Clinical and Pathological Variation of Charcot-Marie-Tooth 1A in a Large Chinese Cohort. Biomed Res Int. 2017;2017:6481367.

[6] Attarian S, Vallat JM et al. An exploratory randomised double-blind and placebo-controlled phase 2 study of a combination of (RS)-baclofen, naltrexone and D-sorbitol (PXT3003) in patients with Charcot-Marie-Tooth disease type 1A.Orphanet J Rare Dis,2014 9:199,1-15.

[7] Study Report CLN-PXT3003-02 CSR. International, multi-center, randomized, double-blind, placebo-controlled phase III study assessing in parallel groups the efficacy and safety of 2 doses of PXT3003 in patients with Charcot-Marie-Tooth Disease type 1A treated 15 months.

[8] PXT3003-Investigator’s Brochure “A low dose, fixed dose combination of (RS)-baclofen, naltrexone hydrochloride and D-sorbitol”. Indication: Charcot-Marie-Tooth Disease Type 1A. Pharnext SA. September 30th, 2019, V7.

[9] *Good Clinical Practice*, National Medical Products Administration, 2020

[10] *Provisions for Drug Registration*, National Medical Products Administration, 2020

[11] *Requirements for Registration Classification and Application Data of Chemical Drugs*, National Medical Products Administration, 2003.

[12] Graham RC, Hughes RAC. A modified peripheral neuropathy scale: the Overall Neuropathy Limitations Scale. *J Neurol Neurosurg Psychiatry.* 2006;77(8):973-976.

[13] Reilly MM, de Jonghe P, Pareyson D. 136th ENMC International Workshop: Charcot- Marie-Tooth disease type 1A (CMT1A)8-10 April 2005, Naarden, The Netherlands. *Neuromuscul Disord.* 2006;16(6):396-402.

[14] Solari A, Laurà M, Salsano E, Radice D, Pareyson D, CMT-TRIAAL Study Group. Reliability of clinical outcome measures in Charcot-Marie-Tooth disease. *Neuromuscul Disord.* 2008;18(1):19-26.

[15] Micallef J, Attarian S, Dubourg O, et al. Effect of ascorbic acid in patients with Charcot-Marie-Tooth disease type 1A: a multicentre, randomised, double-blind, placebo- controlled trial. *Lancet Neurol.* 2009;8(12):1103-1110.

[16] Pareyson D, Reilly MM, Schenone A, et al. Ascorbic acid in Charcot-Marie-Tooth disease type 1A (CMT-TRIAAL and CMT-TRAUK): a double-blind randomised trial. Lancet Neurol. 2011;10(4):320-328.

[17] Karabudak R, Dahdaleh M, Aljumah M, et al. Functional clinical outcomes in multiple sclerosis: Current status and future prospects. Mult Scler Relat Disord. 2015;4(3):192-201.

[18] Panosyan FB, Kirk CA, Marking D, et al. Carpal tunnel syndrome in inherited neuropathies: A retrospective survey. *Muscle Nerve.* 2018;57(3):388-394.

[19] Hogrel J-Y, Payan CA, Ollivier G, et al. Development of a French isometric strength normative database for adults using quantitative muscle testing. Arch Phys Med Rehabil. 2007;88(10):1289-1297.

Appendices

Appendix 1 Drug Label Information

See the Appendix folder for details.

Appendix 2 Contraindicated Medications for CMT1A Subjects During the Study

- ✓ **Baclofen** (Liorésal®), **naltrexone** (Nalorex®, Revia)), **sorbitol** (pharmaceutical dosage forms such as Sorbitol Delleande®, Hépargitol®, Microlax®, Schoum®) and **ascorbic acid** (Vitamin C®, Laroscorbine®) due to a possible effect on the treatment evaluation of the study.
- ✓ **Opioid analgesics** (morphine, fentanyl, hydromorphone, oxycodone, buprenorphine, nalbuphine, Lamaline® and tramadol – including Ixprim®, Topalgic®, Contramal® and Zaldiar®) **and other opioids (such as methadone)** due to their potential interaction with naltrexone.
- ✓ **Codeine** can only be used at ≤ 20 mg per unit dose and painkillers such as **codeine phosphate** are not allowed as they contain 30 mg of codeine per unit dose.
- ✓ **Levodopa** (Sinemet®, Modopar®, Stalevo®, etc.) should not be used in combination with baclofen as the combination may increase adverse effects such as confusion, agitation, and hallucinations.
- ✓ **Centrally acting antihypertensive drugs such as clonidine** (Catapressan®), **methyldopa** (Aldomet®), **moxonidine** (Physiotens®), and **rilmenidine** (Hyperium®) as baclofen may increase the antihypertensive effect (risk of hypotension).
- ✓ Other classes of antihypertensive compounds are allowed, but physicians should be aware that combination therapy with baclofen may potentially increase antihypertensive effects.
- ✓ The use of potent central nervous system (CNS) inhibitors such as **barbiturates, benzodiazepines, and neuroleptics** with baclofen is not permitted due to potentially increased side effects.
- ✓ **Clonazepam** (Rivotril®) may be administered at lower daily doses (≤ 20 drops/day, i.e., 2 mg/day).
- ✓ Treatments that may also cause or aggravate peripheral neuropathy during the trial, such as **antiepileptic drugs phenytoin** (Di-Hydan®) and anticancer drugs such as **vincristine, vinblastine, cisplatin, carboplatin, oxaliplatin, and paclitaxel**.
- ✓ Antiepileptic drugs such as **valproic acid** (Depakine® or Depakote®), **carbamazepine** (Tegretol®), **oxcarbazepine** (Trileptal®), **gabapentin** (Neurontin®), and **pregabalin** (Lyrica®) may be used, but a constant dose should be maintained throughout the trial.
- ✓ **Antidepressants**, including **imipramines**, can be used, but a constant dose should be maintained throughout the trial, as imipramines can potentiate hypotension caused by baclofen, and sedative antidepressants may potentiate CNS inhibition.
- ✓ Drugs that may cause neuropathy:
  - Anti-cancer drugs: Vincristine, vinblastine, cisplatin, carboplatin, paclitaxel;
  - Anti-infective drugs: Metronidazole, nitrofurantoin, and chloramphenicol;
  - Anti-leprosy drugs: Dapsone;
  - Anti-tuberculosis drugs: Isoniazid;
  - Antiretroviral drugs: Didanosine (DDI), Zalcitabine (DDC);

- Antiarrhythmic drugs: Amiodarone;
- Anti-inflammatory drugs (modified therapy): Gold salt;
- Alcohol antidotes: Disulfiram;
- Antiepileptic drugs: Phenytoin sodium;
- Others: Very high doses of alcohol, pyridoxone (Vit B6);

Appendix 3 Laboratory Test

Test Item		Test Indicators
Hematology		White blood cell (WBC)
		Neutrophil count (NEUT)
		Lymphocyte count (LYMPH)
		Red blood cell (RBC)
		Hemoglobin (HGB)
		Platelet count (PLT)
Urinalysis		Protein (PRO)
		Glucose (GLU)
		Leukocyte (LEU)
		Erythrocyte (ERY)
Blood chemistry	Fasting blood glucose	Glucose (GLU)
	Glycated hemoglobin	Glycated hemoglobin (HbA1c)
	Hepatic function	Alanine aminotransferase (ALT)
		Aspartate aminotransferase (AST)
		Gamma glutamyl transpeptidase (GGT)
		Alkaline phosphatase (ALP)
		Total protein (TP)
		Albumin (ALB)
		Total bilirubin (TBIL)
		Direct bilirubin (DBIL)
	Renal function	UREA or BUN
		Creatinine (CREA)
	Electrolytes	Potassium (K)
		Sodium (Na)
		Chloride (Cl)
Female blood pregnancy test		Beta-human chorionic gonadotropin (β-HCG)

Appendix 4 Overall Neuropathy Limitations Scale (ONLS)

Appendix 5 CMTNS-V2

Appendix 6 EQ-5D

Appendix 4, Appendix 5 and Appendix 6 are presented in the Appendix folder.
